# PEtab.jl: advancing the efficiency and utility of dynamic modelling

**DOI:** 10.1093/bioinformatics/btaf497

**Published:** 2025-09-09

**Authors:** Sebastian Persson, Fabian Fröhlich, Stephan Grein, Torkel Loman, Damiano Ognissanti, Viktor Hasselgren, Jan Hasenauer, Marija Cvijovic

**Affiliations:** Department of Mathematical Sciences, Chalmers University of Technology, Gothenburg, SE-412 96, Sweden; Department of Mathematical Sciences, University of Gothenburg, Gothenburg, SE-412 96, Sweden; Dynamics of Livings Systems Laboratory, The Francis Crick Institute, London, NW1 1AT, United Kingdom; Life and Medical Sciences (LIMES) Institute and Bonn Center for Mathematical Life Sciences, University of Bonn, Bonn, 53113, Germany; Computer Science and Artificial Intelligence Laboratory, Massachusetts Institute of Technology, Boston, MA 02139, United States; Department of Mathematical Sciences, Chalmers University of Technology, Gothenburg, SE-412 96, Sweden; Department of Mathematical Sciences, University of Gothenburg, Gothenburg, SE-412 96, Sweden; Department of Mathematical Sciences, Chalmers University of Technology, Gothenburg, SE-412 96, Sweden; Department of Mathematical Sciences, University of Gothenburg, Gothenburg, SE-412 96, Sweden; Life and Medical Sciences (LIMES) Institute and Bonn Center for Mathematical Life Sciences, University of Bonn, Bonn, 53113, Germany; Department of Mathematical Sciences, Chalmers University of Technology, Gothenburg, SE-412 96, Sweden; Department of Mathematical Sciences, University of Gothenburg, Gothenburg, SE-412 96, Sweden

## Abstract

**Summary:**

Dynamic models represent a powerful tool for studying complex biological processes, ranging from cell signalling to cell differentiation. Building such models often requires computationally demanding modelling workflows, such as model exploration and parameter estimation. We developed two Julia-based tools: SBMLImporter.jl, an SBML importer, and PEtab.jl, an importer for parameter estimation problems in the PEtab format, designed to streamline modelling processes. These tools leverage Julia’s high-performance computing capabilities, including symbolic pre-processing and advanced ODE solvers. PEtab.jl aims to be a Julia-accessible toolbox that supports the entire modelling pipeline from parameter estimation to identifiability analysis.

**Availability and implementation:**

SBMLImporter.jl and PEtab.jl are implemented in the Julia programming language. Both packages are available on GitHub (github.com/sebapersson/SBMLImporter.jl and github.com/sebapersson/PEtab.jl) as officially registered Julia packages, installable via the Julia package manager. Each package is continuously tested and supported on Linux, macOS, and Windows.

## 1 Introduction

Understanding the complex nature of dynamic processes such as nutrient signalling, cell division, and apoptosis is one of the central aims in systems biology (Kitano 2002). A powerful tool to help achieve this goal is dynamic modelling, where chemical reactions are modelled stochastically or deterministically (Klipp *et al.* 2016). To date, dynamic models have been used to study a range of processes, from small receptor networks (Becker *et al.* 2010) over bursting gene expression (Zechner *et al.* 2014) to cancer signalling (Fröhlich *et al.* 2018).

Whether a model is based on stochastic rate probability density functions (which describe jump processes where reactions trigger changes in species amount) or on deterministic rate equations [described by Ordinary Differential Equations (ODEs)] (Gillespie 2007), it typically contains unknown parameters, such as reaction rate constants. Thereby, to understand a model’s characteristics, it must be simulated for many parameter sets to validate if it can capture experimental observations. Because most dynamical models in biology typically lack closed-form analytical solutions, numerical simulation methods are necessary. Consequently, efficient modelling requires fast and flexible software.

**Figure 1.**
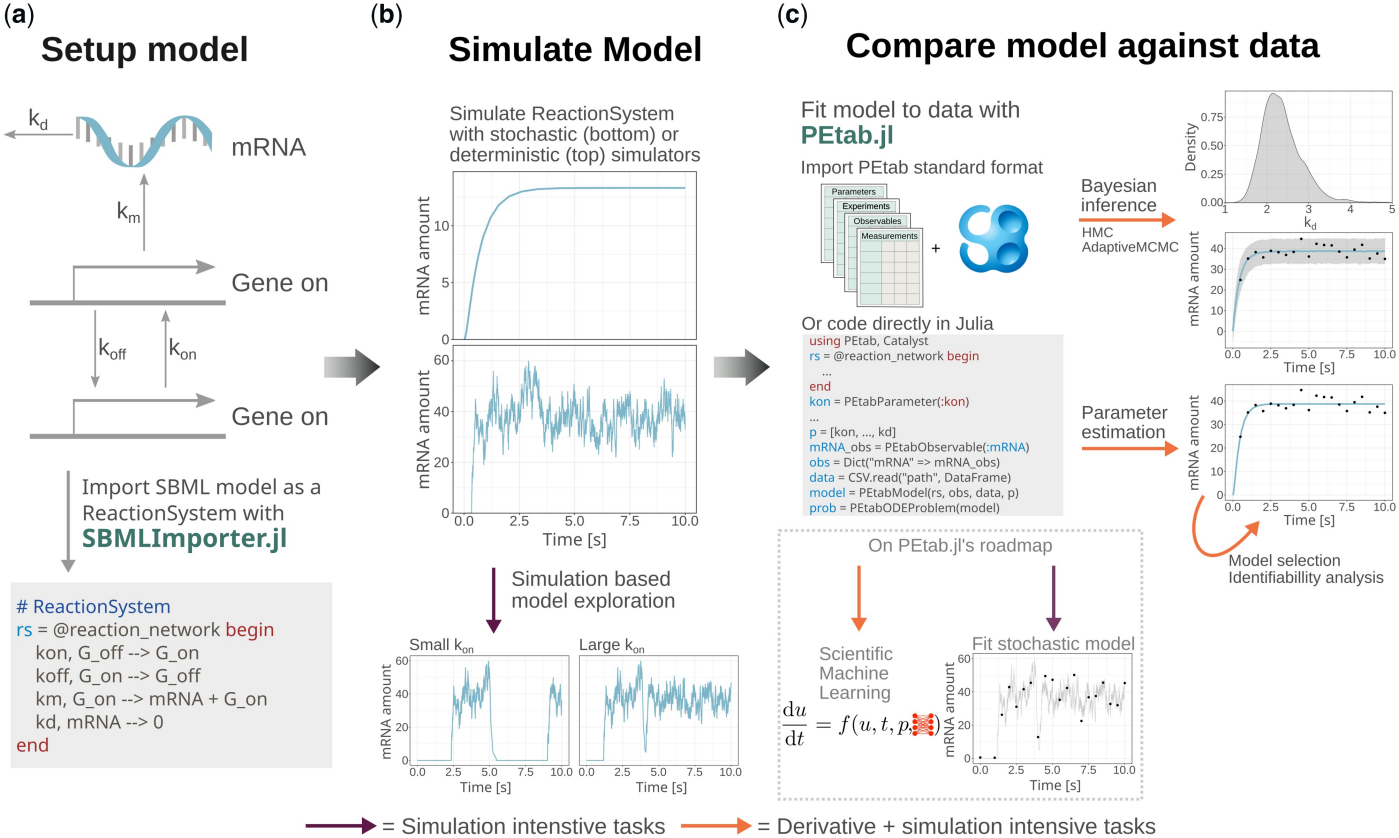
Modelling workflows with PEtab.jl and SBMLImporter.jl. The tools enable an efficient modelling pipeline consisting of three main components. (a) Setting up the model in the SBML format. (b) Simulating the model with stochastic simulators or deterministic ODE solvers. (c) Model fitting with PEtab.jl by linking measurement data to a model via building a likelihood or, given priors, a posterior function.

The Julia programming language has emerged as a promising tool to address the computational challenges encountered in biology (Bezanson *et al.* 2017, Roesch *et al.* 2023). With support for symbolic model pre-processing, automatic differentiation compatibility, adjoint sensitivity analysis, and state-of-the-art ODE solvers (Rackauckas and Nie 2017), Julia’s ecosystem appears ideal for handling tasks like parameter estimation for ODE-based models. To leverage this ecosystem, we developed SBMLImporter.jl, a Julia SBML importer, and PEtab.jl, an importer for parameter estimation problems in the PEtab format (Schmiester *et al.* 2021) (Fig. 1). SBMLImporter enables users to import models with a wide range of features, including events and multiple compartments (e.g. cytosol and nucleus), that are built using SBML exportable tools such as the Copasi graphical interface (Hoops *et al.* 2006). PEtab.jl simplifies model fitting workflows, enabling users to fit models to data across a wide range of scenarios following the PEtab standard (Schmiester *et al.* 2021). Lastly, we performed an extensive benchmarking study to provide guidelines on when and how to use Julia for dynamic modelling.

## 2 Features

SBMLImporter.jl imports SBML models into Catalyst reaction networks (Loman *et al.* 2023) (Fig. 1a). This has several benefits. First, a Catalyst reaction network can be converted into a jump problem (simulated using, e.g. the Gillespie algorithm) or a Langevin SDE problem (Gillespie 2007) for stochastic simulations. Alternatively, for deterministic simulations, it can be converted to an ODE problem (Fig. 1b). For each problem type, all the solvers in the performant DifferentialEquations.jl suite are supported (Rackauckas and Nie 2017). Additionally, Catalyst integrates with other modelling packages, such as Bifurcationkit.jl (Veltz 2020). SBMLImporter.jl does not yet support any SBML extensions, but it provides extensive support for core SBML functionality, comparable to established tools like AMICI (Fröhlich *et al.* 2021) (Table 5, available as supplementary data at *Bioinformatics* online). The most up-to-date list of supported features is available in the online documentation of the package.

PEtab.jl links ODE models to measurement data by creating a likelihood function or given priors, a posterior function (Fig. 1c). Built on the PEtab standard format for parameter estimation (Schmiester *et al.* 2021), the package supports a wide range of scenarios, such as different measurement noise formulas for data collected with different assays, steady-state simulations to model interventions like drug administration in stationary systems, and data gathered under multiple experimental conditions. PEtab problems defined in the standard format can be directly imported. Alternatively, problems can be coded directly in Julia, where the dynamic model is specified as a Catalyst reaction network (Fig. 1c). Extensive tutorials for specifying a problem directly in Julia are available in the PEtab.jl online documentation, while tutorials for creating problems in the standard format are provided in the official PEtab documentation (Schmiester *et al.* 2021). For model likelihood evaluation, PEtab.jl supports both forward and backward gradient computation approaches, suitable for small and large models, respectively (Fröhlich *et al.* 2017). Additionally, the package wraps several numerical optimization methods for parameter estimation and provides Bayesian inference support with state-of-the-art methods such as Hamiltonian Monte-Carlo (Hoffman *et al.* 2014).

## 3 Benchmarking-based guidelines

To provide practitioners with clear guidance on when and how to best utilize our packages, we conducted comprehensive benchmarks (detailed results in Supplementary Text S1, available as supplementary data at *Bioinformatics* online). As many modelling workflows rely on model simulations (Fig. 1), we first evaluated Julia’s stochastic simulators (e.g. Gillespie methods) against PySB and RoadRunner and deterministic simulators (ODE solvers) against the CVODES ODE suite (Hindmarsh *et al.* 2005), using the high-performant Julia CVODE wrapper (Fig. 33, available as supplementary data at *Bioinformatics* online). Next, since ODE model workflows such as Bayesian inference and parameter estimation often benefit from model derivatives (Fig. 1b and c), we evaluated differentiation methods. Lastly, we evaluated parameter estimation performance for ODEs. For these tasks, we compared our results against pyPESTO, which utilizes the AMICI interface to SUNDIALS’ ODE suite (Hindmarsh *et al.* 2005, Fröhlich *et al.* 2021). We considered AMICI because it is more efficient than COPASI (Fröhlich *et al.* 2021), and comparable to the high-performance toolbox RoadRunner with respect to model simulations (Fig. 33, available as supplementary data at *Bioinformatics* online). Further, it supports direct import of problems in the PEtab format, allowing us to perform all evaluations on ODE models using problems with real experimental data from the PEtab benchmark collection (Hass *et al.* 2019, Schmiester *et al.* 2021).

### 3.1 Optimal ODE solver for model simulations is problem dependent

Most ODE models in systems biology are nonlinear and require numerical solvers for simulation. Previous studies have benchmarked CVODES and LSODA (Städter *et al.* 2021) solvers but did not evaluate Julia’s DifferentialEquations.jl suite (Rackauckas and Nie 2017). Using PEtab.jl, we tested 31 solvers from DifferentialEquations.jl and two from CVODES suite (Tab. S3) across 29 published biological models (Table 1, available as supplementary data at *Bioinformatics* online), ranging from 3 to 500 states (ODEs), and representing a broad spectrum of biological processes such as cellular molecular models (e.g. signalling), SIR models (e.g. Covid-19 spread), to phenomenological models (e.g. spiking, cell differentiation).

We divided the ODE solvers into four categories: (i) CVODES stiff solvers, (ii) non-stiff DifferentialEquations.jl solvers, (iii) stiff DifferentialEquations.js solvers, and (iv) DifferentialEquations.jl composite solvers that automatically switch between stiff and non-stiff solvers. Informally, stiffness in ODE models arises when interactions occur on varying time scales, with some being fast (e.g. phosphorylation) and others slow (e.g. translation). Overall, non-stiff solvers failed in 22%–31% of cases, particularly for molecular models, while composite solvers struggled with steady-state simulations, making stiff solvers the most reliable option (Fig. 2, available as supplementary data at *Bioinformatics* online). Julia’s solvers were the fastest for models with up to 16 species, while for medium-sized models (20–75 species), Julia and CVODES were comparable. Stiff solvers performed best in molecular models, while composite solvers worked well for SIR and some phenomenological models. Considering solver families, Rosenbrock methods were most efficient for smaller molecular models, while BDF methods excelled in medium-sized ones. For larger network models, there is no clear best choice (Fig. 3, available as supplementary data at *Bioinformatics* online).

In summary, stiff solvers perform well for molecular models, and composite solvers scored well for SIR models and a subset of phenomenological models (e.g. cell differentiation). The optimal solver choice depends on the size of the ODE model. To aid in selecting an optimal solver, we provide a flowchart (Fig. 12, available as supplementary data at *Bioinformatics* online). For additional details, see Supplementary Text S1.2, available as supplementary data at *Bioinformatics* online.

### 3.2 Automatic differentiation accelerates model derivative computations

Common modelling workflows, such as parameter estimation and sensitivity analysis, rely on accurate gradient computations (Raue *et al.* 2013). However, gradient evaluations can, especially for larger models, dominate runtime. Traditionally, gradients have been computed using forward sensitivities for small ODE-models, whereas for large models, adjoint sensitivity analysis has been used. Unlike these approaches, PEtab.jl can leverage forward-mode automatic differentiation (AD) for small models and reverse-mode AD to efficiently compute vector Jacobian products (VJPs) in the adjoint sensitivity analysis computations. To assess the impact of AD-assisted gradients on performance, we compared PEtab.jl to AMICI, a CVODES-based solver.

Benchmarking 18 biological models, we found that forward-mode AD outperformed traditional forward sensitivity analysis in AMICI across most models. Forward-mode AD also exhibited better scalability, with gradient-to-loss runtime ratios frequently lower than the number of parameters (Fig. 2, available as supplementary data at *Bioinformatics* online).

For large ODE models (>100 states + parameters), forward-mode AD becomes impractical, as its runtime scales with the product of the number of parameters (*p*) and states (*m*); O(p×m). Rather, in this regime, adjoint sensitivity analysis scales better. For five larger benchmark models, AMICI demonstrated the lowest failure rate (9.5% versus 64% for the best PEtab.jl setup), although PEtab.jl achieved higher speedups and greater accuracy using interpolation-based adjoints and Enzyme AD for VJP computation (Fig. 5, available as supplementary data at *Bioinformatics* online).

In summary, for smaller models (≤75 ODEs and parameters), whether molecular, SIR, or phenomenological, forward-mode automatic differentiation is generally faster than traditional forward sensitivity analysis. For larger molecular models, the adjoint algorithms in Julia are fast but currently less reliable than the adjoint sensitivity analysis approach available via AMICI based on CVODES. To help choose a gradient setup, we have compiled a flowchart (Fig. 13, available as supplementary data at *Bioinformatics* online). For additional details, see Supplementary Text S1.3, available as supplementary data at *Bioinformatics* online.

### 3.3 PEtab.jl often facilitates training efficiency of models

ODE-based models must often be fitted to data by estimating unknown parameters, which corresponds to solving a continuous optimization problem. To evaluate PEtab.jl for parameter estimation, we benchmarked it against pyPESTO, which uses AMICI for model simulations, across 19 models. For pyPESTO, we tested the Newton-trust region Fides optimization algorithm (Fröhlich and Sorger 2022), with three Hessian approximations: default, BFGS, and Gauss-Newton. PEtab.jl was tested with Fides (BFGS and Gauss-Newton) and the Interior-point Newton (IPNewton) method from Optim.jl. Additionally, full Hessians were used when the computation time was ≤2 seconds. For each model, we performed 1000 optimizations and used the runtime per converged start to the global minimum as the evaluation criterion. Highlighting the efficiency of PEtab.jl, it outperformed pyPESTO in 15 out of 19 models. pyPESTO primarily performed better for models with pre-equilibrium (steady state) simulations.

The superior efficiency of PEtab.jl was primarily due to being faster. Compared to pyPESTO’s Fides optimizer, PEtab.jl was faster in 16/18 models, with an average speedup of 3.38x. For additional details, see Supplementary Text S1.4, available as supplementary data at *Bioinformatics* online.

## 4 Discussion

Dynamic modelling plays a pivotal role in understanding cellular dynamics, which are essential for our understanding of the functioning of living organisms. Accelerating this process improves both its efficiency and practical utility. To support this, we developed SBMLImporter.jl, and PEtab.jl. Both packages have already been extensively used in several research projects and are continuously being developed. In the future, we aim to include new methods for computationally demanding tasks such as support for scientific machine learning models (Rackauckas *et al.* 2020). However, to fully harness the potential of Julia for modelling in biology, the next step would be to develop tools like pyPESTO and D2D that support the entire modelling pipeline (Raue *et al.* 2015, Schälte *et al.* 2023). This presents an exciting opportunity for the community to collaborate and build upon existing frameworks, paving the way for more efficient and accessible computational biology tools.

## Supplementary Material

btaf497_Supplementary_Data

## Data Availability

SBMLImporter.jl and PEtab.jl are available on GitHub (github.com/sebapersson/SBMLImporter.jl and github.com/sebapersson/PEtab.jl) as officially registered Julia packages, installable via the Julia package manager.
