# Supplementary material for: PEtab.jl: advancing the efficiency and utility of dynamic modelling
Source: Bioinformatics. 2025 Sep 9;41(9):btaf497. doi: 10.1093/bioinformatics/btaf497 (PMC12457741; doi:10.1093/bioinformatics/btaf497)
Supplement: btaf497_Supplementary_Data [file btaf497_supplementary_data.pdf]

PEtab.jl: Advancing the Efficiency and Utility of Dynamic Modelling

Supplementary Data

Sebastian Persson[1,2], Fabian Fröhlich[3], Stephan Grein[4], Torkel Loman[5], Damiano Ognissanti[1,2], Viktor Hasselgren[1,2], Jan Hasenauer[4,6,7], Marija Cvijovic[1,2,*]

1 Department of Mathematical Sciences, Chalmers University of Technology, Sweden.

2 Department of Mathematical Sciences, University of Gothenburg, Sweden.

3 Dynamics of Livings Systems Laboratory, The Francis Crick Institute, London NW1 1AT, United Kingdom

4 Life and Medical Sciences (LIMES) Institute, University of Bonn, Bonn 53113, Germany

5 Computer Science and Artificial Intelligence Laboratory, Massachusetts Institute of Technology, United States of America

6 Computational Health Center, Helmholtz Zentrum München Deutsches Forschungszentrum für Gesundheit und Umwelt (GmbH), Neuherberg 85764, Germany

7 Department of Mathematics, Technische Universität München, Garching 85748, Germany

Contents

1 Benchmarking-based Guidelines for Practitioners **3**

1.1 Julia stochastic simulators are often most efficient . . . 3

1.2 Optimal ODE solver for model simulations is problem dependent . . . 4

1.3 Automatic differentiation accelerates model derivative computations . . . 9

1.4 PEtab.jl often facilitates training efficiency of models . . . 12

1.5 Materials and methods . . . 14

1.5.1 Stochastic model formulation . . . 16

1.5.2 ODE model formulation . . . 16

1.5.3 Parameter estimation for ODE models - problem formulation . . . 16

1.5.4 Gradient computations . . . 17

1.5.5 Computing $\nabla G(\boldsymbol{\theta})$ via automatic differentiation . . . 17

1.5.6 Computing $\nabla G(\boldsymbol{\theta})$ via forward sensitivity equations . . . 17

1.5.7 Computing $\nabla G(\boldsymbol{\theta})$ via adjoint sensitivity analysis . . . 19

1.5.8 Hessian computations . . . 19

1.5.9 Robustness of results . . . 20

1.5.10 Disclosure - usage of AI-assisted tools . . . 20

2 Extended discussion on benchmark results **21**

2.1 Extended discussion on ODE solvers . . . 21

2.2 Extended discussion on parameter estimation results . . . 21

References **23**

Additional tables **26**

Additional figures **32**

1 Benchmarking-based Guidelines for Practitioners

To evaluate the Julia programming language for workflows with dynamic models in systems biology and produce guidelines for practitioners, we used `SBMLImporter.jl` and `PEtab.jl` to perform comprehensive benchmarks. Since many modelling workflows rely on model simulations (main text Fig. 1), we first used these tools to evaluate Julia's stochastic simulators (e.g. Gillespie methods) against `PySB` and RoadRunner and deterministic simulators (ODE solvers) against the Sundials ODE suite [15]. Next, since ODE model workflows such as Bayesian inference, parameter estimation, and scientific machine learning often benefit from model derivatives (main text Fig. 1b-c), we evaluated differentiation methods. Lastly, as a case study, we evaluated parameter estimation performance for ODEs. For the latter two tasks, we compared our results against `pyPESTO`, which utilizes the `AMICI` interface to SUNDIALS' ODE suite [15, 9]. We compared against AMICI is because, with respect to model simulations, it is more efficient than COPASI [9] and comparable to the high-performance RoadRunner toolbox, as confirmed by our benchmarks for five systems biology benchmark problems of varying size (Fig. 33). Moreover, AMICI supports direct import of problems in the PEtab format, allowing us to perform all evaluations on ODE models using problems with real experimental data from the PEtab benchmark collection [14, 36]. For evaluating ODE solvers, we used the Julia interface to the Sundials CVODE solver. This allows for a fairer comparison of the actual ODE solver, as all evaluations of the ODE model right-hand sides are performed in Julia. Furthermore, both ours (Fig. 33) and others benchmarks show that the Julia Sundials wrapper is efficient [22].

1.1 Julia stochastic simulators are often most efficient

Stochastic models in biology can rarely be solved analytically, requiring numerical methods for model simulation. For a given parameter set, stochastic simulators randomly simulate system reactions over time to produce random trajectories [21] (main text Fig. 1b). Therefore, to characterise model behaviour even for a single parameter set, many simulations are needed. A recent study demonstrated that the simulators in Julia's `JumpProcesses.jl` are faster than those in BioNetGen, Matlab SimBiology, Copasi, and GillesPy [22]. However, this study did not consider `RoadRunner` and `PySB` [23, 43]. `RoadRunner` is the simulation engine in several modeling packages such as Tellurium, while `PySB` in particular supports the network-free NFsim method, which was designed for simulating large chemical reaction networks [37]. To benchmark these tools, using `SBMLImporter.jl` we compared four `JumpProcesses.jl` simulators: the direct SSA (Direct) [11], the sorted direct SSA (SortingDirect) [25], Rejection SSA (RSSA), and Composition-Rejection SSA (RSSACR) methods [39, 40, 41], against `RoadRunner`'s Direct SSA method, and `PySB`'s SortingDirect and NFsim [37] methods. We considered the five differently-sized models used for benchmarking in [12] (Tab. 2) that describe processes such as a multistate signalling (multistate, 18 reactions), three-site phosphorylation (multisite3, 288 reactions), epidermal growth factor receptor signalling (egfr net, 3749 reactions), B-cell receptor signalling (BCR, 24 388 reactions), and IgE receptor signalling (FcϵRI $\gamma 2$, 58 276 reactions).

To account for simulation initialization overhead, we simulated the models for several time intervals. `JumpProcesses` was fastest for all models (Fig. 1). For the smallest model (Fig. 1a), the SortingDirect method was noticeably faster than `RoadRunner` (2.6 fold speed up at $t = 10^5$ Fig. 1a). The `PySB` SSA simulator had a large initial overhead, probably due to network generation, which can be sidestepped by directly calling BNGL for model simulations. However, for long simulation intervals where initial overhead has a minor effect, `PySB`'s SortingDirect was comparable for longer simulation times (JumpProcesses had 1.28 fold speed up at $t = 10^5$). For the two medium-sized models (Fig. 1b, f), RSSACR performed best and was more than four

times faster than the best `PySB` method (multisite3 4.1 and egfr net 28 fold speed up compared to `PySB`'s SortingDirect Fig. 1d-e, f). NFsim could not simulate the large BCR model, likely due to disjoint patterns (molecules of the same species not linked by intermediates). For the largest FcϵRI-γ2 model, RSSACR was roughly 4.6 times faster than NFsim (Fig. 1e).

Given that network-free simulators like NFsim often perform well for large models with low molecule counts that have many reactions but few rules (rule-based models), it was unexpected that RSSACR was faster than NFsim for the FcϵRI γ2 model (has 19 rules and at $t = 1000$ around 6000 molecules). To investigate to what extent RSSACR performs better than NFsim for rule-based models, we tested a set of multisite phosphorylation models with an increasing number of phosphorylation sites where NFsim showed good behaviour [37]. At 5 sites (multsite5, 7680 reactions and 20 rules, Fig 1g) NFsim surpassed RSSACR (2.8 times faster).

Regardless, JumpProcesses's SortingDirect and RSSACR solvers (loaded via SBMLImporter) performed best overall. Both simulators weakly depend on the order of reactions in the model; for example, to effectively scan the reaction list, the SortingDirect method sorts reactions based on their probabilities. This can be consequential. When importing models into Julia from BioGenNet files via `ReactionNetworkImporters.jl` [17], due to the file format, high-probability reactions often occur early in the reaction list. Therefore, the runtime was, in particular, affected for the Direct and RSSA methods. For example, the Direct was 1.02, 1.12, and 1.70 times faster for multistate, multisite2, and egfr-net models (when loaded using ReactionNetworkImporters), while with SortingDirect sorted direct, the difference was only 0.99, 1.03, and 1.18 (Fig. 7). For the large FcϵRI-γ2 model, RSSACR's performance was still notably impacted by reaction order (1.56 times faster with net-file import Fig S1).

In summary, Julia simulators are efficient for small and large models, often being at least four times faster than `PySB` and `RoadRunner` for models with > 200 reactions. For large models with low molecule numbers and few rules, network-free simulators can be efficient.

1.2 Optimal ODE solver for model simulations is problem dependent

Most ODE models in systems biology are non-linear and lack analytical solutions requiring numerical ODE solvers to simulate them. The choice of solver algorithm can drastically impact simulation times [30, 38] and even the convergence of the solver [38]. However, given the multitude of available algorithms selecting the best one is non-trivial. To provide guidelines, a recent study extensively evaluated the Sundial's and LSODA's solvers for a wide range of models [38] but did not evaluate the ODE solvers available within the Julia DifferentialEquations library [30]. We used `PEtab.jl` to assess model simulation time, reliability (simulation failure frequency), and accuracy of 31 solver algorithms in the `DifferentialEquations.jl` suite, and 2 algorithms from the Sundials suite (Tab. 3). We tested these on 29 benchmark problems varying in size from 3 to 500 states (ODEs), representing a wide spectrum of biological processes such as cellular molecular models (e.g. signalling), SIR models (e.g. Covid spread), to phenomenological models (e.g. spiking, cell differentiation) (Tab. 1).

We divided the ODE solvers into four categories, i) Sundial stiff solvers, ii) non-stiff DifferentialEquations solvers, iii) stiff DifferentialEquations solvers, and iv) composite solvers that automatically switch between stiff and non-stiff solvers. Informally, stiffness in ODE models arises when interactions occur on varying time scales, with some being fast (e.g., phosphorylation) and others slow (e.g., translation). Such dynamics are thought to be common in biology [38], and for such models, stiff ODE solvers are beneficial. We found that 11/11 non-stiff solvers failed to simulate 22-31% of the models (blue colour Fig. 2a). Failures occurred primarily for molecular models. Notably, non-stiff solvers and composite solvers failed for models with

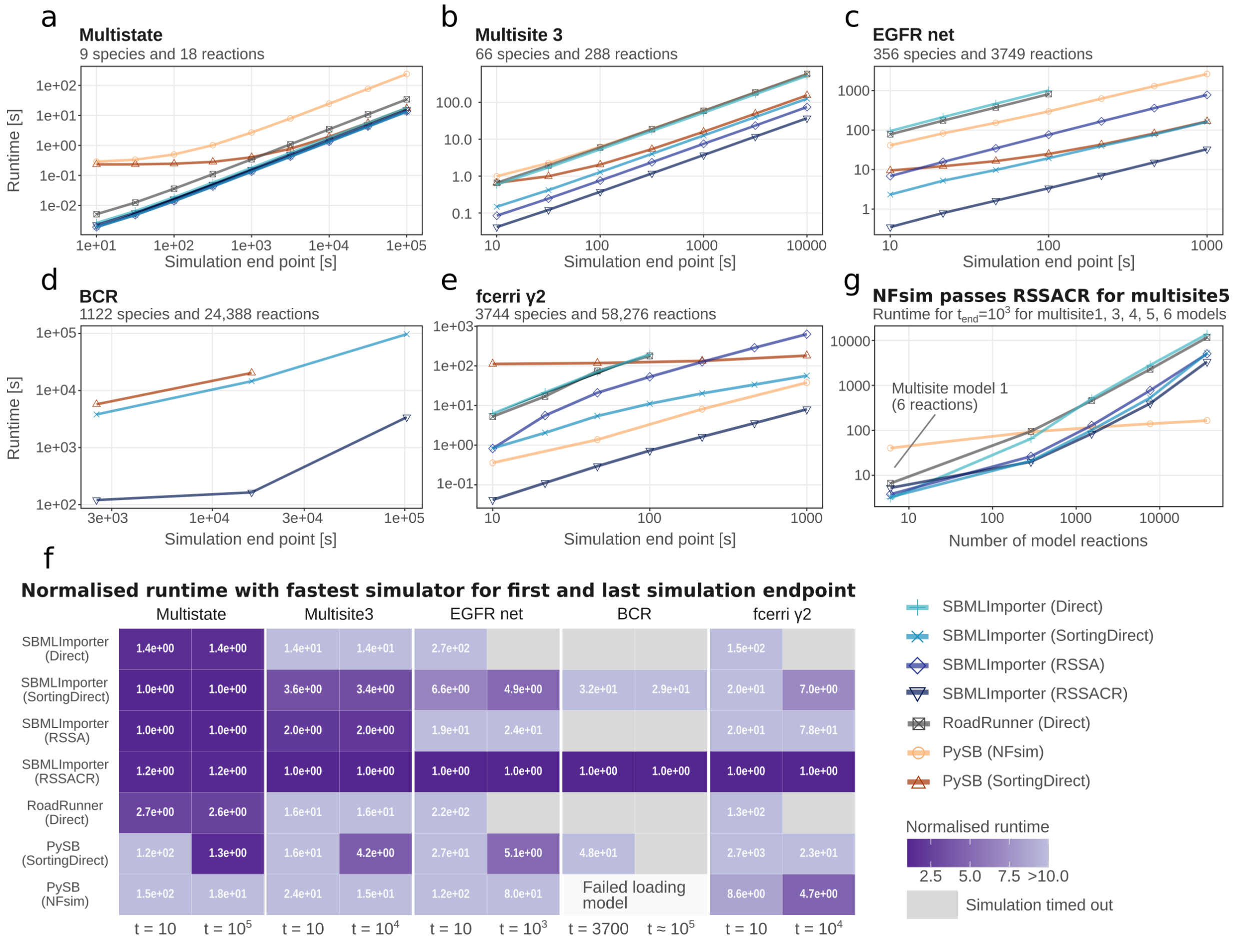

Figure 1: Evaluating stochastic simulators for JumpProcesses (models loaded via SBMLImporter), RoadRunner and PySB. a-f) For `SBMLImporter.jl`, we tested the direct SSA (Direct), sorting direct SSA (SortingDirect), the reaction SSA (RSSA) and composition-reject (RSSACR) methods simulated via `JumaProcesses.jl`. For RoadRunner, the direct SSA method, and for `PySB`, the sorting direct SSA, as well as the network-free NFsim methods. To account for potential initialisation overhead, models were simulated for different endpoints, e.g., the Multistate model was evaluated when simulating to endpoints $t = 10^1, ..., 10^5$. Evaluated models are **a)** a multistate signalling model, **b)** three-site phosphorylation model, **c)** an epidermal growth factor receptor signalling model, **d)** a B-cell receptor signalling model, and **e)** and IgE receptor signalling model. NFsim failed to simulate the BCR model. `RoadRunner` and SMBLImporter (via JumpProcesses's Direct and RSSA methods) could simulate the BCR model, but failed to complete the simulations within our imposed 4-day time limit (and are hence not included for this model). **f)** Runtime normalised with the fastest method at the first and last simulation end-point for the benchmarks in panel a-e. For example, for the Multistate model at $t = 10^5$ SBMLImporter (via JumpProcesses) SortingDirect was around 2.5 faster than RoadRunner. **g)** Runtime across five multisite phosphorylation models (multisite 1, 3, 4, 5, 6 see Tab. 2) of increasing complexity when simulating each method to a final time of 1000s.

steady-state simulations (4/4 and 2/4 models, respectively), which are commonly used to model interventions like drug administration to stationary systems. Thus, a stiff or composite solver should be used to reduce the risk of simulation failures, which matters during model training, as upon failure, most training algorithms reduce step size, causing longer training times or even the discontinuation of training (and convergence problems). Further, if failures happen near the optimal parameter values, the training process may be biased and never converge.

In addition to reliability, simulation time is important as model training often requires up to $10^3 - 10^4$ model simulations [14]. `DifferentialEquations.jl` algorithms were fastest for smaller models with up to 16 states (avg. 4.0 fold speedup of Julia stiff (blue) solvers vs Sundi-

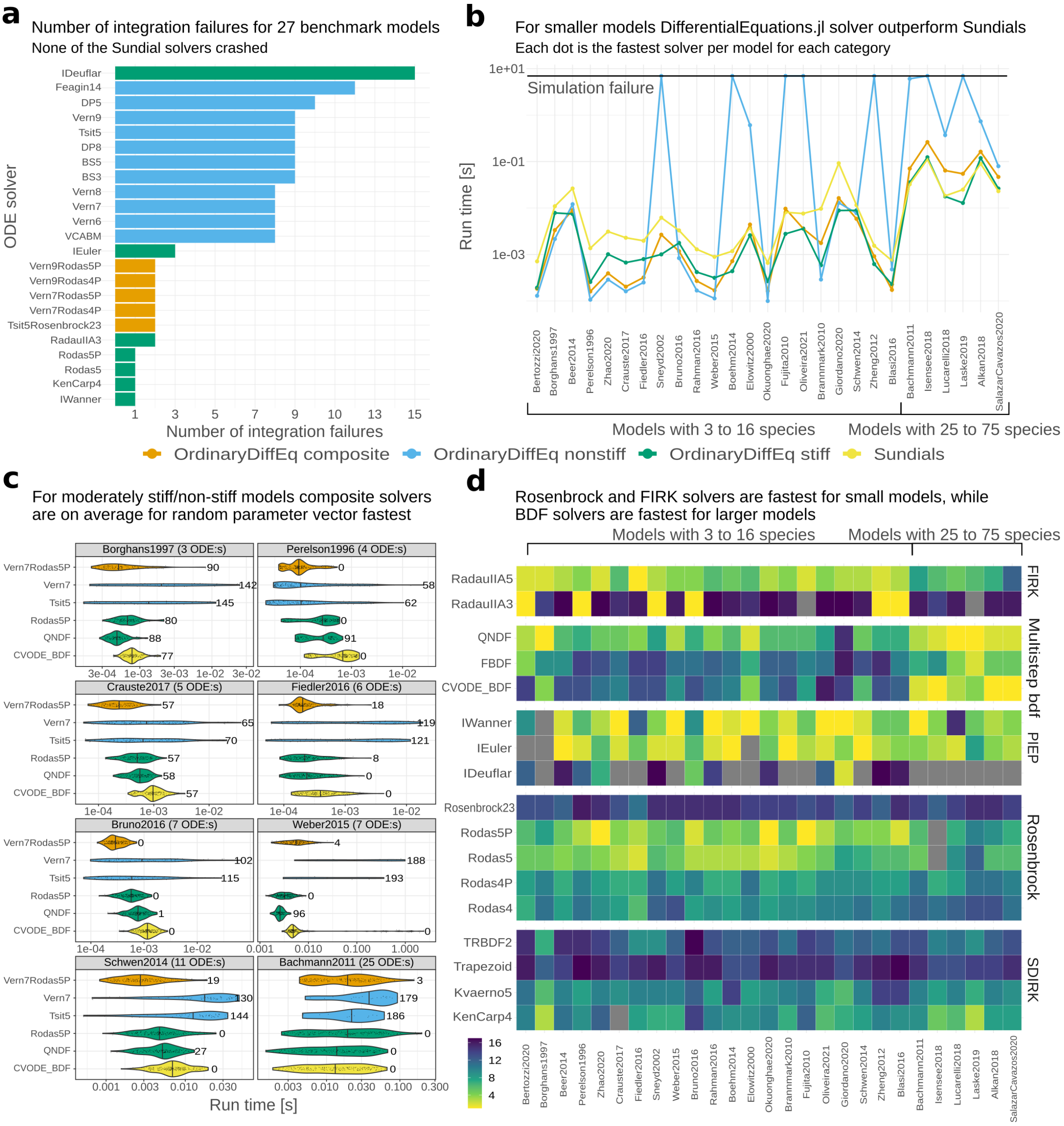

Figure 2: Benchmarking two Sundial and 31 DifferentialEquations ODE solvers for 27 models. Note, that the two bigger models (Chen and Smith) were excluded from this benchmark due to the runtime required to test all solvers. **a)** Total number of integration failures across all models for each solver (Tab. 3). **b)** The best average runtime (averaged over 5 repetitions) for each solver category (Tab. 3). The x-axis is sorted based on the number of states (ODEs) for each model. **c)** Runtime across 200 random parameter vectors for 8 selected models using non-stiff, stiff, and composite solvers that performed well in panel b). The centre line of each violin plot denotes the median, and the number at the end of the violin shows the number of integration failures for that solver, e.g., for the Perelson model, the QNDF failed for 91 out of 200 parameter vectors. **d)** Runtime ranking per model for a large selection of stiff solvers grouped by their respective solver family. A bright yellow colour denotes a good ranking, while a grey colour indicates integration failure.

als (yellow)), and DifferentialEquations and Sundials were mostly tied for most medium-sized models of 20-75 states (Fig. 2b, avg. 1.2 fold speedup of Julia stiff solvers vs Sundials). Consistent results hold for stricter tolerances (Fig. 8d-e). Noticeably, for ten models (2 molecular, 4 SIR, and 4 phenomenological) with ≤ 9 ODEs, non-stiff solvers were the fastest. We suspect

this is because the benchmarks were performed at the reported parameter values for which the ODE solution might be well-behaved.

To simulate a real-world scenario where optimal parameters are unknown, we measured the runtime for 200 random parameter vectors (Fig. 2c, Fig. 8, Fig. 9). For the two molecular models (Fiedler and Weber) stiff solvers performed best (yellow and green violins). For phenomenological models (Borghans, Bruno, Crauste, Perlson), composite or stiff performed best (yellow, green and orange violins) and for SIR models, composite (2/4) and non-stiff (2/4) performed best (Fig. 8c). We further looked at two models (Schwen, Bachmann) where stiff/composite solvers performed best at the reported values, and consistently stiff solvers scored best (yellow and green violins Fig. 2c), that is had fewer simulation failures while being efficient. To further investigate simulation failures, we inspected ODE solver error codes (Fig. 9). Noticeably, for the numerically most challenging models (Crauste and Borghans), composite solvers can get stuck (take $>$ 15 min), and while stiff solvers are better, there is no clear best choice for these challenging models (extended discussion).

Given the strong performance of stiff solvers, we next looked closer at solver families. Albeit solving the same problem, stiff solvers can be divided into families such as multi-step BDF methods and single-step Rosenbrock methods, each tailored to specific problem types. We found, in line with previous DifferentialEquations benchmarks [30], that Rosenbrock solvers are among the fastest and most accurate solvers for small models ($\leq$ 16 states) (Fig. 2d and Fig. 8b), while for medium-sized molecular models (25-75 states) multi-step BDF-solvers are among the fastest. Clear exceptions to this trend are the small Borghans spiking Ca^{2+} model (CVODE-BDF performed best Fig. 2c), and the small SIR Giordinni model (composite solvers performed best Fig. 8c).

For larger models, the performance of stiff solvers depends on the efficiency of the employed linear solver. This is because, at minimum, one linear system must be solved at each time step, and with a dense Jacobian, this scales at least with *(number of ODEs)*$^{2.376}$ [7]. Julia offers different direct (FastLU, RFLUF, Lapack, Dense, KLU) and implicit (GMRES) solvers, some of which can exploit sparsity in the ODE. For up to medium-sized models ($\leq$ 75 states), the default automatically chosen linear solver for both CVODE-BDF and several DifferentialEquations solvers performed well (dark orange Fig. 3a-c).

To study the impact of linear solvers for larger models in a realistic setting for 100 random parameter vectors, we looked at three randomly selected medium-sized models and two newly introduced large models of 113 (Smith) and 500 (Chen) states. For the medium-sized models, BDF solvers (e.g. QNDF and CVODE-BDF) at the default setting performed best (Fig. 3d-h). Meanwhile, for the Smith model, CVODE-BDF combined with a stiff KLU-solver (green violin) was almost twice as fast as the best Julia solvers, while for Chen, the SDIRK solvers (KenCarp4 and Kvaerno5) with a KLU linear solver were more than twice as fast as CVODE-BDF. For both bigger models, Julia BDF-solvers (QNDF and FBDF) frequently failed (Fig. 3i and Fig. 10). The strong performance of the KLU solver might be because it was developed for circuit simulations [6, 7], and circuits might have a topology similar to extensive cellular networks.

In summary, Rosenbrock solvers perform well for smaller molecular models (e.g., signalling) and BDF methods for medium-sized models. For large network models, BDF and SDIRK solvers should be compared. Composite solvers scored well for SIR models and a subset of phenomenological models (e.g., cell differentiation). This result indicates that the choice of the best solver is problem-dependent.

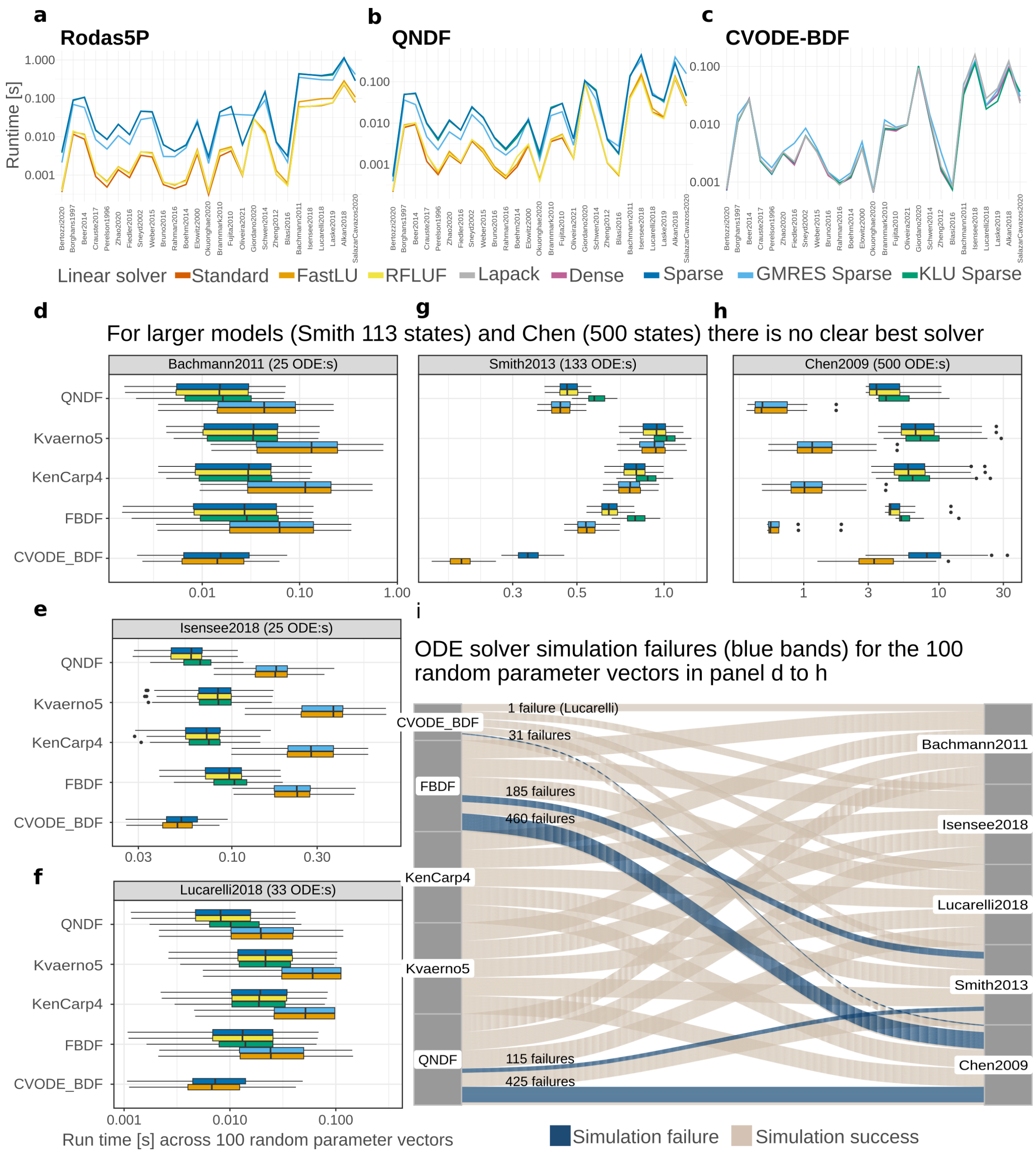

Figure 3: Comparison of different linear solvers for stiff ODE-solvers. a-c) Runtime for the Sundial CVODE-BDF and `DifferentialEquations.jl` Rodas5P and QNDF solvers on 27 benchmark models, sorted by size (3-75 states). The colours indicate the linear solver used in the stiff solver time-stepping, with "standard" being the default option and "sparse" indicating the use of a sparse Jacobian. **d-h)** Runtime across 100 random parameter vectors for three medium-sized ODE models, and the third (Smith) and second largest (Chen) largest ODE models in the benchmark collection. **i)** Integration failures are denoted by the blue bands for the 100 random parameter vectors and ODE-solver options in panel d-h, and the number next to the band is the total number of failures for all linear solver combinations for a solver. For example, regardless of linear solver the Julia KenCarp4 solver failed zero times for the Chen model, while the Julia FBDF solver failed for most cases. Results were consistent for different linear solvers, as example, for the FBDF solver, regardless of linear solver the ODE solver ran into integration failure for 92/100 vectors ($92 = 460/5$).

1.3 Automatic differentiation accelerates model derivative computations

Common modelling tasks such as model training to find optimal model parameters, identifiability analysis to quantify uncertainty in model predictions/parameters, and sensitivity analysis to identify drug targets typically benefit from accurate gradients [31]. However, for these tasks, gradient computations make out a substantial portion of the runtime (e.g. for the Bachmann model, loss and gradient runtime are 0.02 and 1.1 seconds, respectively). Traditionally for small ODE models, the gradient is computed using forward sensitivities by solving an expanded ODE system [32], where the runtime scales with the size of the expanded system; number of original ODEs (n) times the number of parameters (p) $\mathcal{O}(n \times p)$. For larger ODE models, adjoint sensitivity is preferred as the runtime scales approximately as $\mathcal{O}(n + p)$, with a higher initial cost compared to forward approaches [5, 24]. In contrast to traditional approaches, `PEtab.jl` can utilize forward-mode automatic differentiation (AD) for small models and reverse-mode AD to efficiently compute vector Jacobian products (VJPs) in the adjoint sensitivity analysis computations [24]. To assess whether AD-assisted gradients can accelerate derivative computations and subsequently speed up common modelling tasks, we compared `PEtab.jl` against AMICI [9] - an interface to the Sundials suite.

To compare `PEtab.jl` with AMICI for up to medium-sized models, we selected 18 benchmark models that represent a wide range of biological processes (12 cellular molecular, 3 SIR and 3 phenomenological; Tab. 1). We used the CVODE-BDF solver for AMICI, while for `PEtab.jl`, we employed the Rodas5P and QNDF solvers based on previous benchmarks (Fig. 2). First, we focused on the runtime of the loss function. For the 8 smaller models (≤ 20 parameters), there were small differences (AMICI median speedup of 0.9 against both QNDF and Rodas5P), meanwhile, for larger models, AMICI had an advantage (median speedup of 2.1 and 2.6 against QNDF and Rodas5P respectively; Fig. 4a, Tab. 6).

Next, we evaluated gradient runtimes. Forward-mode automatic differentiation (AD) outperformed the forward sensitivity approach in AMICI for the majority of models (Rodas5P 67% and QNDF 72% models with median 1.9 and 1.6 fold speedup, respectively), regardless of whether the model is molecular, SIR, or phenomenological (Fig. 4b). AD gradients also have better scalability, as only for AD gradients the runtime ratio between the loss and gradient was frequently smaller than the number of parameters (28% AMICI, 89% QNDF, and 94% Rodas5P of the models; Fig. 4c, Tab. 6). Similar results hold for a hybrid approach, in which sensitivity equations, rather than the gradient of the loss functions, are computed via forward-mode AD (Fig. 11a). For higher order derivatives, we found that the loss-Hessian runtime ratio was often smaller than the number of parameters squared (72% QNDF and 53% of the models Fig. 4f, Tab 7). As a coarse rule of thumb, we found Hessian computations feasible (≤ 2 seconds) for models with up to 20 parameters.

As the efficiency of the forward-mode AD methods was surprising, we next analysed AD tuneable options to understand why. In a naive AD implementation, a single directional derivative is computed per forward pass, thus as many passes as parameters are needed to compute the gradient [1], resulting in a linear scaling. `ForwardDiff.jl` employs a technique called chunking, which allows for a tunable number of directional derivatives to be computed per forward pass [33]. We tested the runtimes of chunked and un-chunked AD for three models where we successively increased the number of parameters on which to compute the gradient. Chunked gradients scaled better (Fig. 4d and Fig. 11), but it should be noted that chunk size is a tuning parameter, and the default value may not be optimal (Fig. 4e and Fig. 11). Why chunking improves performance is unclear, but at least for simple cases, compiler output suggests that chunked gradient operations frequently use efficient vectorised CPU SIMD instructions. There-

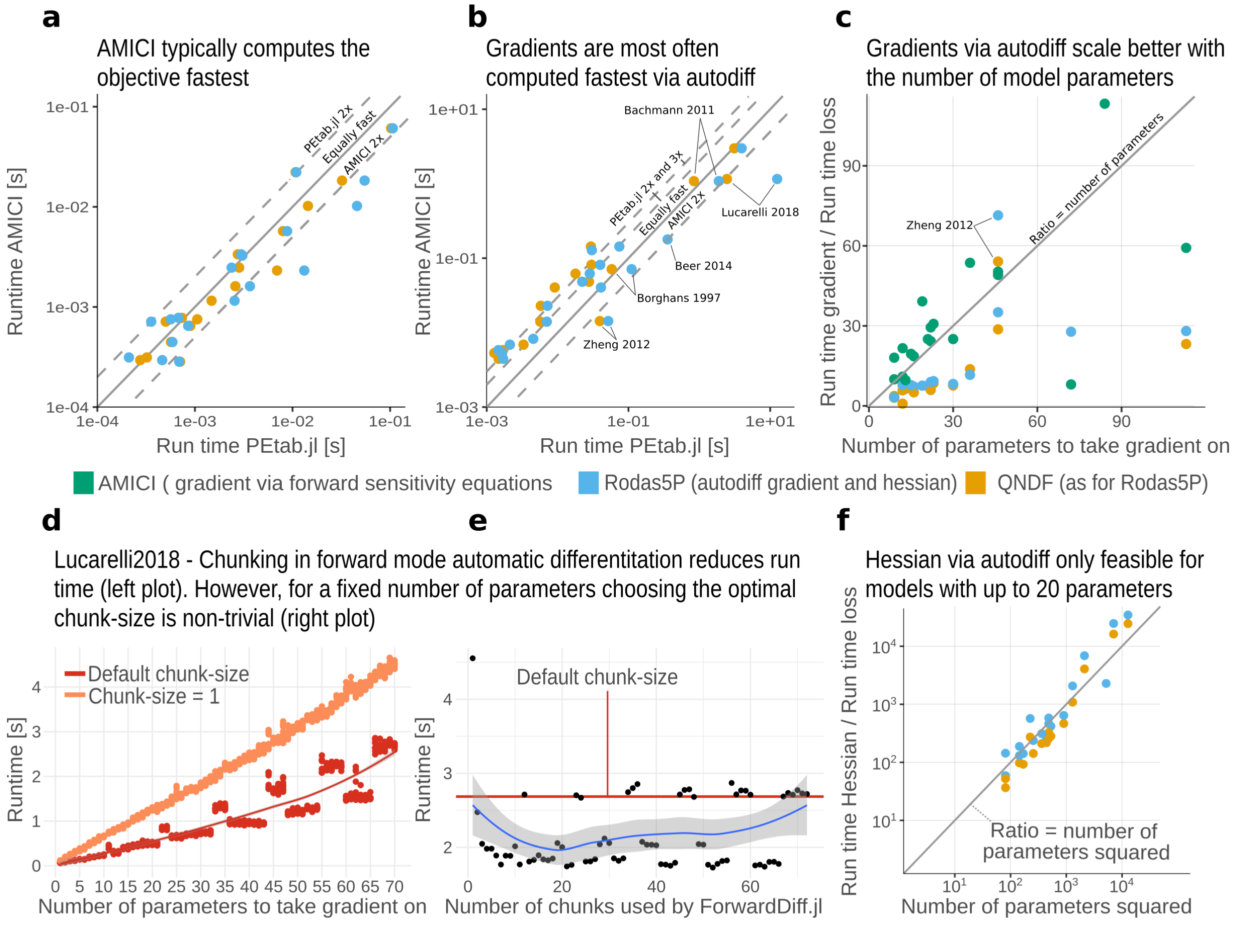

Figure 4: Objective function and gradient benchmark for smaller models. **a)** Runtime evaluation for the loss (objective function) at the reported parameter values. AMICI uses CVODE-BDF, while the Julia PEtab importer uses the Rodas5P and QNDF ODE solvers. Numerical values for panels a-c are in Tab. 6 **b)** Runtime evaluation for the gradient at reported parameter values. AMICI computes the gradient by solving for the sensitivities via an expanded ODE system, while the Julia importer computes the gradient via forward-mode automatic differentiation. **c)** Ratio between the runtime for the gradient in b) and loss in a). The number of model parameters is given on the x-axis. **d)** Gradient runtime for the Lucarelli model when increasing the number of parameters using forward-mode AD without chunking and with the default number of chunks. **e)** Gradient runtime for the Lucarelli model when taking the gradient on all parameters and testing all chunk sizes. **f)** Ratio between runtimes for Hessian calculation. The number of model parameters squared is specified on the x-axis. Absolute values are given in Tab. 7.

fore forward-mode AD might have an advantage over other approaches because it effectively leverages modern CPU architectures.

For large ODE models with more than 100 ODEs + parameters, such as extensive signalling or metabolic networks, forward approaches are impractical (e.g., a single forward-mode AD gradient for the Smith model requires $\approx$ 100 seconds). Here adjoint sensitivity analysis can drastically reduce runtimes [5, 24], but runtimes are still substantial, so selecting the best adjoint algorithm is important. `PEtab.jl` supports both the interpolation and quadrature algorithms from `SciMLSensitivity.jl`, along with reverse-mode AD approaches to compute the vector Jacobian product (VJP) in the adjoint ODE [29, 24]. To compare these algorithms with AMICI, we selected five representative benchmark models ranging from 17 to 655 parameters+states (1 small, 2 medium-sized, and 2 large) and evaluated gradient runtime at 50

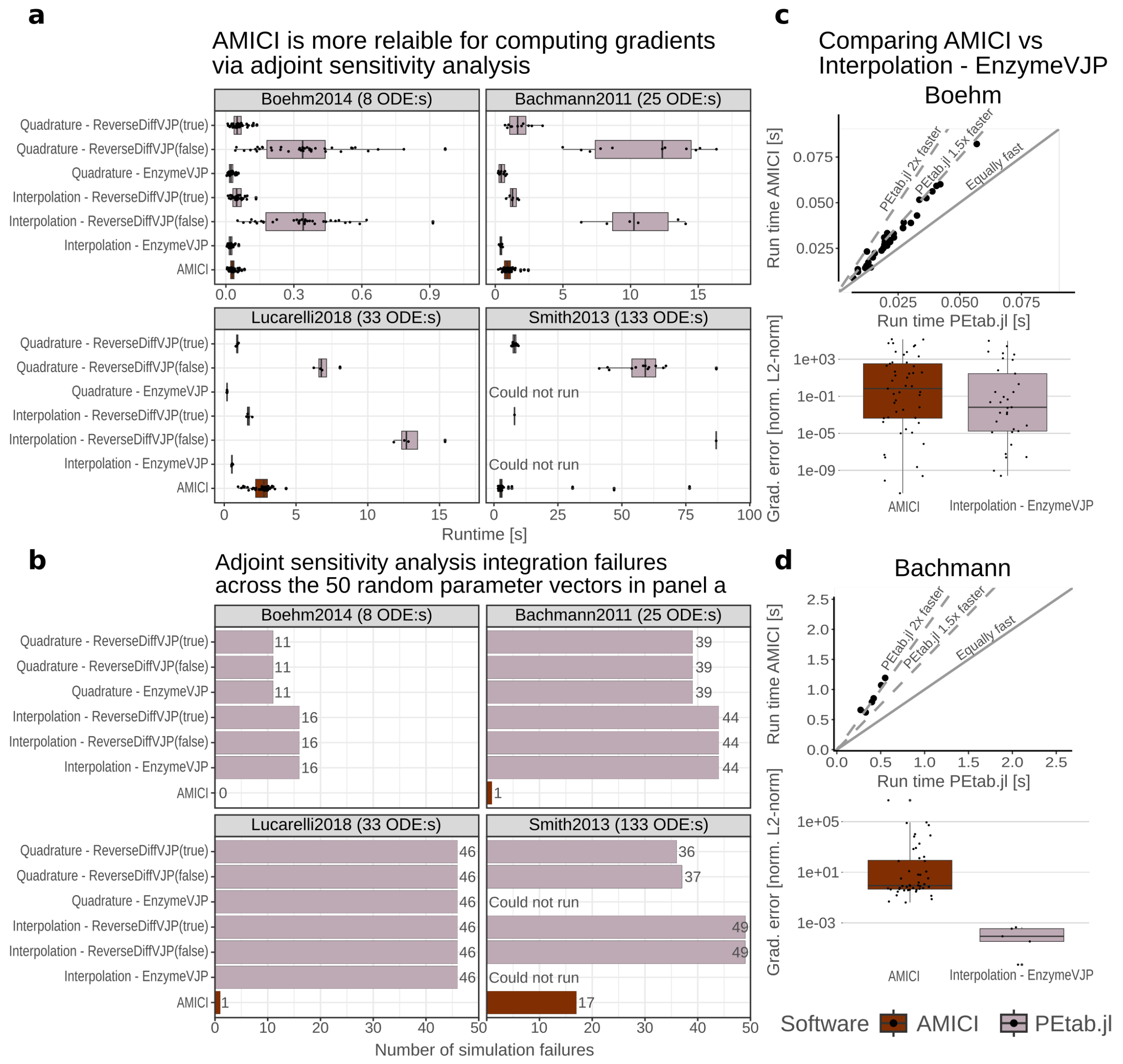

Figure 5: Benchmarking adjoint sensitivity gradient computation methods for five larger ODE models. a) Runtime across 50 random parameter vectors when computing the gradient via adjoint sensitivity analysis. We tested two approaches for `PEtab.jl`: the quadrature and interpolation approach in `SciMLSensitivity.jl`, with different ways of computing the vector-Jacobian-product in the adjoint ODE. For instance, "ReverseDiffVJP(true)" refers to using `ReverseDiff.jl` with a compiled tape (true). For Julia, we used the CVODE-BDF solvers as they performed best. **b)** Integration/gradient failures when solving the adjoint ODE for the 50 random parameter vectors and ODE-solver options in panel a). **c,d)** Runtime (upper) and accuracy (lower) comparison between AMICI and the interpolation approach in SciMLSensitivity using the Enzyme automatic differentiation library to compute the VJP for the Boehm and Bachmann models. When SciMLSensitivity does not fail, it consistently outperforms AMICI. Gradients were computed by $\|\nabla f_{adjoint} - \nabla f_{ref}\|_2$ where the reference gradient ∇f_{ref} was computed using a high-order finite-difference method.

randomly selected parameters. AMICI proved the most reliable (9.5% and 64% average fail rate for AMICI and the best SciMLSensitivity algorithm, respectively; 5b). Furthermore, `PEtab.jl` failed to load the large Chen model (655 parameters + states) as it required ≥30GB of RAM. Excluding the failed runs, `PEtab.jl` was faster (avg. speedup of 1.3 and 2.1 for the Boehm and Bachmann models respectively; Fig. 5a, c-d), and was often more accurate (Fig. 5c-d) when we used the interpolation approach and the Enzyme automatic differentiation library to compute the VJP [27]. This is likely because the Enzyme automatic differentiation approach to com-

pute the VJP in the adjoint ODE is more efficient than computing it directly via sparse-matrix representations as done in AMICI.

In summary, for smaller models ($\leq$ 75 ODE + parameters), whether they are molecular, SIR, or phenomenological, forward-mode automatic differentiation is generally faster than the forward sensitivity analysis approach for gradient computations. For larger molecular models, the adjoint algorithms from SciMLSensitivity are fast but currently less reliable than the adjoint sensitivity analysis approach accessible via AMICI based on CVODES.

1.4 PEtab.jl often facilitates training efficiency of models

ODE-based models in systems biology often have unknown parameters, such as reaction rate constants, that must be estimated from experimental data. This training problem corresponds to a continuous optimization problem, and to accurately evaluate the quality of a model to, for example, compare it against other models (hypotheses), it is crucial to identify the best fit, preferably rapidly. To evaluate `PEtab.jl` we set up a benchmark against `pyPESTO` which leverages AMICI for model simulations using the same benchmark models as for the gradient evaluation (12 cellular molecular, 3 SIR and 3 phenomenological; Tab. 1). For `pyPESTO`, we used the Newton-trust region Fides training algorithm [8] and tested three different approaches to approximate the Hessian: default, BFGS, and Gauss-Newton. For `PEtab.jl`, we tested Fides with the BFGS and Gauss-Newton (GN) Hessian approximation, along with the `Optim.jl` [26] Interior-point Newton (IPNewton) method with GN hessian approximation. Moreover, since `PEtab.jl` can compute the full Hessian for models with a Hessian runtime $\leq$ 2 seconds, we tested Fides and IPNewton with a full Hessian. For each model, we performed 1000 optimization runs using Latin-Hypercube-generated random start guesses. Model-specific convergence plots can be found in Fig. 14-32.

Two important model training evaluation criteria, exemplified by the Fiedler model (Fig. 6a-c), are overall runtime and the number of runs that converged to the best likelihood value. For the Fiedler model, the Interior Point Newton method (IPNewton, yellow) had a higher convergence rate compared to Fides BFGS (grey) (5.8% and 0.2% respectively). However, BFGS was faster (IPNewton: 66.6h; BFGS: 1.51h). A summary of the overall runtime and the number of converged runs is the overall efficiency (OE)-score, which is the number of converged runs divided by the runtime [42]. The OE-score provides the number of converged starts, which can be expected per unit of time, meaning that larger values are better. For the Fiedler mode, the BFGS achieved the highest and thus best score (BFGS 1.32OE and IPNewton 0.87OE).

When considering the OE-score across all models, `PEtab.jl` outperformed `pyPESTO` for 15/19 models (3 SIR, 1 phenomenological and 11 cellular molecular models), with `pyPESTO` performing better for the Borghans, Elowitz, Lucarelli and Zheng models. The Zheng model requires a pre-equilibrium (steady-state simulation), for which AMICI has sophisticated handling. For the Lucarelli model `pyPESTO` had a notably higher number of converged starts when using the Gauss-Newton Hessian approximation, but for the Oliveira and Isensee models `PEtab.jl` had more converged runs. Looking closer at two models with differences in converged runs (Crauste and Isensee), computing the Gauss-Newton approximations proved challenging (even with low 10^{-8} ODE solver tolerances), implying that the impact of using different ODE solvers and gradient methods likely is what affected convergence (extended discussion, Tab. 4). Lastly, for Elowitz, Borghans and Beer, neither software frequently converged, so results should be interpreted carefully. The low convergence rates are likely related to model characteristics, both Elowitz and Borghans are stiff and oscillate which is hard to handle [28]. Beer has many parameters to estimate (72) where a subset corresponds to the activation time of an

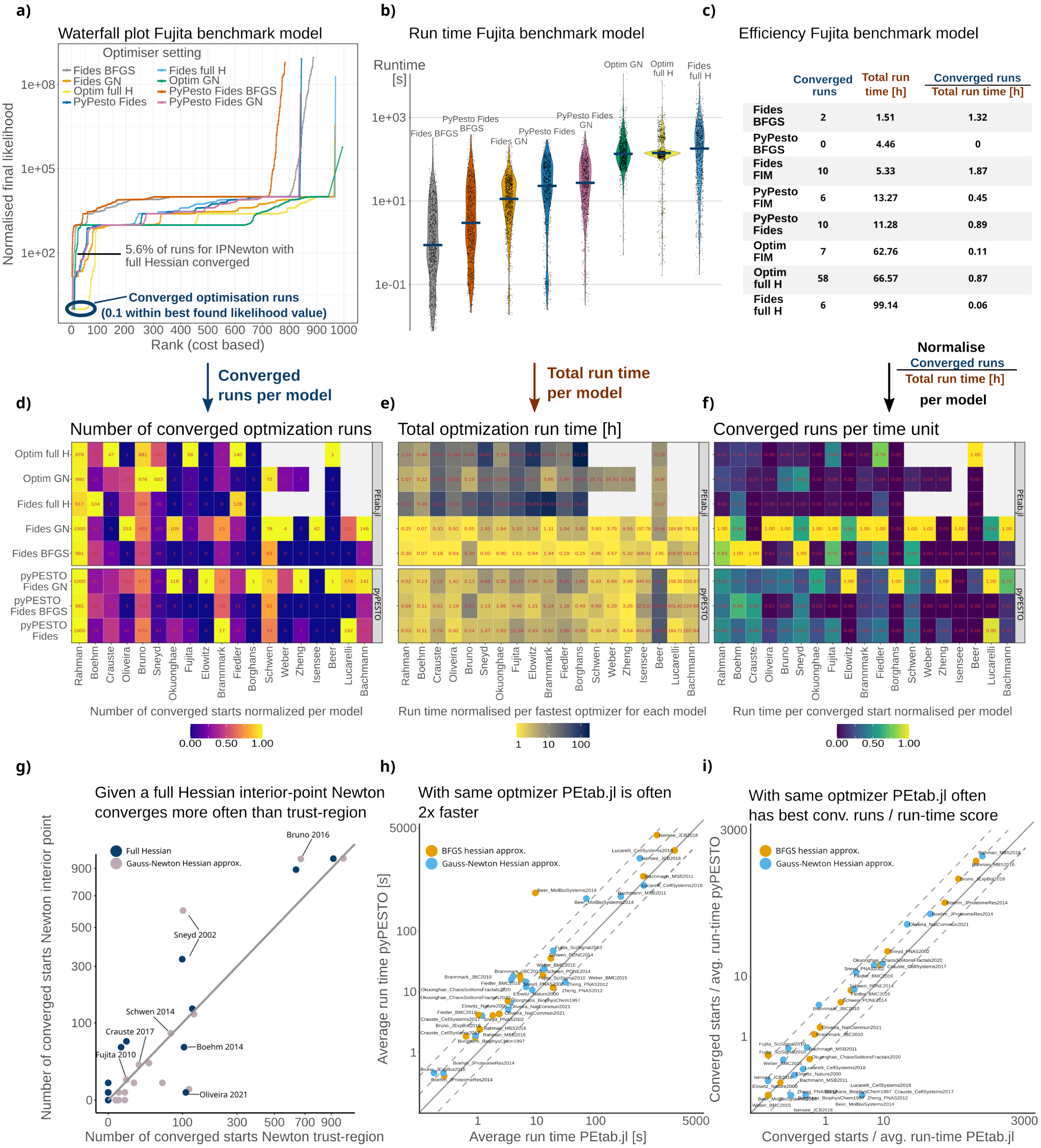

Figure 6: Parameter estimation benchmark for `pyPESTO` against `PEtab.jl`. a) Waterfall plot for the Fujita model from 1000 optimisation runs. The second word indicates the Hessian computation method, such as full Hessian refers to computing the full Hessian via forward mode AD **b)** Runtime for the runs in a) for the Fujita model. **c)** Parameter estimation statistics for the Fujita model. **d)** Number of converged runs from running 1000 optimization runs for each model (x-axis, sorted by the number of parameters) and optimizer option (y-axis) from random starting guesses. The color indicates the number of converged runs normalized by the best method per model. White boxes denote that the method was too expansive to run. **e)** Total wall time in hours to perform the 1000 optimization runs for each model and optimizer. The color indicates runtime normalized with the fastest option per model. **f)** Converged run per time unit (panel a divided by panel b) normalized per model, where matches the values in the heatmap. **g)** Number of converged runs for the interior-point Newton method in `Optim.jl` and the Fides trust region method. **h)** Average runtime for `pyPESTO` and `PEtab.jl` when using the Fides optimizer. **i)** Comparison of the number of converged runs divided by runtime when using the Fides optimizer for `pyPESTO` and `PEtab.jl`, as in h).

event, which require special methods [4]. Regardless, training options where we used a Hessian approximation instead of the full Hessian consistently achieved the best score (18/19 models), as computing the latter is computationally demanding.

`PEtab.jl` often achieved the best OE score due to its faster speed. To quantify the speed difference, we compared the runtime when using the Fides optimizer for both software. For all but two models (Zheng, Lucarelli), `PEtab.jl` was faster (avg. speedup 2.31 fold with respect to Fides GN and 4.44 fold with respect to Fides BFGS Fig. 6h).

While providing a full Hessian did not improve the overall OE score, it did improve convergence for some types of models (for 0/3 SIR, 1/3 phenomenological, 5/7 molecular models). For instance, in the Fiedler model, the best Hessian approximation converged in only 1.0% of runs (Fig. 6a-c), whereas the IPNewton method with full Hessian converged in 5.8% of runs. However, providing the full Hessian does not always yield better results, as seen for the Brannmark, Bruno, Borghans, Elowtiz, Okuonghae, Oliveira and Rahman models.

Lastly, we evaluated the earlier claim that trust-region methods often perform best by comparing results from the Newton interior-point and trust-region Newton methods [14]. When we used the Gauss-Newton Hessian approximation, similar to earlier studies [31, 14], the trust-region method was indeed best (for 12/16 tested models). However, when we computed the full Hessian, the interior-point method outperformed the trust-region method (for 11/13 of tested models Fig. 6e). Further, for 7/13 and 2/13, the complete Hessian interior point and trust-region methods, respectively, performed better than the `pyPESTO` trust-region with Gauss-Newton approximation.

In summary, computing a full Hessian can improve training convergence, especially for molecular models. However, it comes at a computational cost, which questions its applicability for models with 15 or more parameters. When we can compute the full Hessian, interior-point algorithms often perform better, whereas it is feasible only to compute a Gauss-Newton Hessian approximation, trust-region methods tend to perform better.

1.5 Materials and methods

We evaluated `PEtab.jl` against `pyPESTO` and AMICI on previously published models with real experimental data from the PEtab benchmark collection [14, 36]. A summary of key features and benchmarking information for each model is available in Tab. 1. For testing gradients, we selected a subset of models with a wide range of parameters to estimate (9 - 155), covering several biological features (molecular, SIR and phenomenological) and a wide range of computational features such as parameter-dependent initial conditions with different observable function and noise models, preequilibration, and log-transformations. To benchmark parameter estimation performance, we used the same models as for the gradients, except for the larger Smith and Chen models as `PEtab.jl` achieved poor performance when computing gradients for large models via adjoint sensitivity analysis (Fig. 5). We divided the models into three categories based on which process they modelled: (1) cellular molecular models, (2) SIR models and (3) phenomenological models (e.g. spiking, virus growth). The classification is not based on trajectories (e.g. some SIR models might have similar ones to phenomenological models), but rather the process they model, e.g. SIR models all focus on disease progression in a population.

For stochastic model simulators, we used the five models previously evaluated in [12, 22], Tab. 2, and for additional testing of NFsim, we used a series of multi-site phosphorylation models with increasing number of binding sites from [37]. These models cover a range of scenarios, like phosphorylation, receptor dynamics, and signalling. Moreover, as different stochastic simulators

Table 1: PEtab benchmark problems as specified in the GitHub repository. Denoted are the number of states (ODEs), parameters to estimate, and which specific benchmarks the models were used for (computing the ODE solution, computing the gradient, and performing parameter estimation, respectively). The last column categorizes the model types, including cellular molecular models, SIR models and Phenomenological models.

**ID**	**# states**	**# P. est.**	**ODE sol.**	**Grad.**	**P. est.**	Model type
Alkan_SciSignal2018	36	56	×			Cellular molecular (Signalling - DNA repair)[1]
Bachmann_MSB2011	25	113	×	×	×	Cellular molecular (Signalling - JAK2/STAT5)[1]
Beer_MolBioSystems2014	4	72	×	×	×	Phenomenological (Product synthesis - Indigoidine)
Bertozzi_PNAS2020	3	3	×			SIR
Blasi_CellSystems2016	16	9	×			Cellular molecular (histone acetylation)[1,3]
Boehm_JProteomeRes2014	8	9	×	×	×	Cellular molecular (Signalling - STAT5 phosphorylation)[1]
Borghans_BiophysChem1997	3	23	×	×	×	Phenomenological (Spiking - Ca^{2+} oscillations)
Brannmark_JBC2010	9	22	×	×	×	Cellular molecular (Signalling - insulin)[1,3]
Bruno_JExpBot2016	7	13	×	×	×	Phenomenological (Synthesis - abscisic acid)
Chen_MSB2009	500	155	×	×[4]		Cellular molecular (Signalling - MAPK and PI3/Akt)[1]
Crauste_CellSystems2017	5	12	×	×	×	Phenomenological (Cell differentiation - CD8 T)
Elowitz_Nature2000	8	21	×	×	×	Phenomenological (Synthesis - synthetic fluorescent oscillators)
Fiedler_BMC2016	6	22	×	×	×	Cellular molecular (Signalling - Raf/MEK/ERK pathways)[1]
Fujita_SciSignal2010	9	19	×	×	×	Cellular molecular (Signalling - EGFR pathway)[1]
Giordano_Nature2020	13	50	×			SIR
Isensee_JCB2018	25	46	×	×	×	Cellular molecular (Signalling - PKA-II activation)[1,3]
Laske_PLOSComputBiol2019	41	13	×			Cellular molecular (Cell cycle - viral cell-cycle)[1]
Lucarelli_CellSystems2018	33	84	×	×	×	Cellular molecular (Signalling - Smad complex formation)[1]
Okuonghae_Chaos2020	9	16	×	×	×	SIR
Oliveira_NatCommun2021	9	12	×	×	×	SIR
Perelson_Science1996	4	3	×			Phenomenological (Virion production - HIV)[2]
Rahman_MBS2016	7	9	×	×	×	SIR
SalazarCavazos_MBoC2020	75	6	×			Cellular molecular (Receptor dynamics - EGFR phosphorylation)[1]
Schwen_PONE2014	11	30	×	×	×	Cellular molecular (Receptor dynamics - Insulin binding)[1]
Smith_BMCSystBiol2013	133	24	×	×		Cellular molecular (Signalling - Insulin)[1]
Sneyd_PNAS2002	6	15	×	×	×	Cellular molecular (Receptor dynamics - IP_3)[1]
Weber_BMC2015	7	36	×	×	×	Cellular molecular (Cell protein-lipid interactions at Golgi network)[1,3]
Zhao_QuantBiol2020	5	28	×			SIR
Zheng_PNAS2012	15	46	×	×	×	Cellular molecular (DNA modification - methylation)[1,3]

are often suited to a certain model size, these models cover a wide range of sizes (Tab. 2).

1. These models are commonly referred to as cellular molecular models because they share a similar goal, aiming to provide a molecular description of a specific part of the cell.
2. Similar to SIR model. Coarse model of body virion population instead of the infected population as in SIR
3. Models with steady-state simulation
4. Tested for adjoint - but failed to load as it required more than 30GB RAM memory

1.5.1 Stochastic model formulation

Stochastic model simulations in systems biology are often carried out using so-called jump processes. These are continuous-time, discrete-space simulations, simulating the actual reaction events of the system and their impact on its state. How these jump processes are generated from a model is described by stochastic chemical kinetics [21]. In practice, these stochastic chemical kinetics-based jump process simulations are carried out using some algorithm, of which Gillespie's (or the stochastic simulation) algorithm is the most well-known one [10, 11].

Implementation in this study: To simulate stochastic chemical kinetics, we use the `JumpProcesses.jl` Julia package [18, 44]. Specifically, we used Gillespie's direct method, the sorting direct method, and the rejection and composition-rejection SSA methods [11, 25, 39, 40, 41]. We also use the `PySB` and `RoadRunner` simulation packages [23, 43]. We used their implementation of Gillespie's direct method (`RoadRunner`), the sorting direct method (`PySB`), or NFsim (`PySB`) [37]. All trialled simulators are exact methods. This means that they sample the exact jump process described by stochastic chemical kinetics. Hence, their performance can be directly compared without taking tolerance, error, or similar measures into account.

To ensure only the computational cost of the actual simulations was measured, we disabled all saving of intermediary solution states (i.e., we only saved the solution at the initial and final time points). For `JumpProcesses`, we used the `save_positions = (false, false)` option (which disables solution saving before and after jumps). For PySB, we used the `n_runs = 1` (limiting simulation runs to a single simulation) and `gml = 50000000` (a safety which throws an error when molecule numbers are high, increasing it is required to enable simulation for large models) options. We used the RoadRunner's Julia binding (with no additional options).

1.5.2 ODE model formulation

We consider ODE models that can be expressed as:

$$\frac{\mathrm{d}\mathbf{u}}{\mathrm{d}t} = \dot{\mathbf{u}} = f\big(\mathbf{u}, \boldsymbol{\theta}_p, x^{(e)}(t)\big) \quad \text{with } \mathbf{u}(t_0) = \mathbf{u}_0^{(e)}(\boldsymbol{\theta}_p) \tag{1}$$

Here, $\boldsymbol{\theta}_p$ are the parameters that govern the system's dynamics of the system. The solution vector $\mathbf{u} \in \mathbb{R}^m$ has m components and depends on a condition-specific input function $x^{(e)}(t)$ and an initial value function $\mathbf{u}_0^{(e)}$. Note that the initial values can depend on the parameters themselves.

Implementation in this study: To solve the ODE models `PEtab.jl` uses the Julia `DifferentialEquations.jl` or `Sundials.jl` packages [16, 30]. For the gradient and parameter estimation benchmarks, we used the solver tolerances $abstol = reltol = 1 \times 10^8$ for both AMICI and our PEtab importer. For the ODE solver benchmark (Fig. 2), the tested solvers are reported in Tab. 3.

1.5.3 Parameter estimation for ODE models - problem formulation

We define $\boldsymbol{\theta} = (\boldsymbol{\theta}_p, \boldsymbol{\theta}_n)$ as a set of parameters, where $\boldsymbol{\theta}_n$ are parameters not a part of the ODE system and $\boldsymbol{\theta}_p$ are a part of the ODE system (or initial values). The aim is to estimate these parameters by finding the set of parameters $\boldsymbol{\theta}$ that minimizes the function $G(\boldsymbol{\theta})$, which describes the deviation between model output and experimental measurement data y:

$$\min_{\boldsymbol{\theta}} G(\boldsymbol{\theta}) = \sum_{e=1}^{E} \sum_{i=1}^{N_e} g^{(e,i)} \Big(h^{(e,i)}\big(\mathbf{u}(\boldsymbol{\theta}_p, t^{(e,i)}, x^{(e)}(t^{(i)})), \boldsymbol{\theta}_n\big), \sigma^{(e,i)}\big(\mathbf{u}(\boldsymbol{\theta}_p, t^{(e,i)}, x^{(e)}(t^{(i)})), \boldsymbol{\theta}_n\big), y^{(e,i)} \Big), \tag{2}$$

where the observable function h links the ODE system to the observed data, and σ describes the measurement noise formula. The problem is subject to upper and lower bounds $\mathbf{lb} \leq \boldsymbol{\theta} \leq \mathbf{ub}$. Note that the input and initial value functions can modify parameters that are part of the ODE system but not included in $\boldsymbol{\theta}$. Additionally, the dynamic parameter can be condition-specific, specifically $\boldsymbol{\theta}_p = (\boldsymbol{\theta}_p^{(c)}, \boldsymbol{\theta}_p^{(e)})$, where c denotes constant between

Table 2: PEtab benchmark problems for evaluating stochastic model simulators. Listed are the number of model species, reactions, and parameters and since all models are generated from the rule-based BioNetGen language, the number of rules.

ID	# Species	# Reactions	# Parameters	# Rules
Multistate	9	18	9	4
Egfr net	356	3749	43	23
BCR	1122	24 388	128	76
Fceri $\gamma 2$	3744	58 276	26	19
multisite1	6	6	9	4
multisite3[1]	66	288	9	12
multisite4	258	1536	9	16
multisite5	1026	7680	9	20
multisite6	4098	36 864	9	24
multisite7	16 386	172 032	9	28

simulation/experimental conditions. In the PEtab standard, the function $g^{(e,i)}$ and, in extension, G represent negative log-likelihoods. So given a normal measurement noise $g^{(e,i)}$ would, dropping super-scripts and time t for ease of notation, look like;

$$g(\boldsymbol{\theta}) = 0.5\log(2\pi) + 0.5\log\big(\sigma(\mathbf{u}(\boldsymbol{\theta},\mathrm{x}),\boldsymbol{\theta}_n)^2\big) + \frac{0.5}{\big(\sigma(\mathbf{u}(\boldsymbol{\theta},\mathrm{x}),\boldsymbol{\theta}_n)\big)^2}\big(h(\mathbf{u}(\boldsymbol{\theta},\mathrm{x}),\boldsymbol{\theta}_n) - y\big)^2 \tag{3}$$

1. We downloaded multisite models from `http://michaelsneddon.net/nfsim/pages/models/models.html`, where due to a mistake, the code for multisite 2 is actually the one for multisite 3 (hence we did not benchmark multisite 2)

1.5.4 Gradient computations

For small models, the gradient ∇G is traditionally computed using forward sensitivity analysis (with less than 100 parameters) and adjoint sensitivity analysis for larger models [20]. In addition, our PEtab importer can use forward-mode automatic differentiation to compute both the gradient and the Hessian [33, 1].

1.5.5 Computing $\nabla G(\boldsymbol{\theta})$ via automatic differentiation

`PEtab.jl` computes the gradient via forward-mode automatic differentiation by propagating dual numbers, $x + y\epsilon$ with $\epsilon^2 = 0$, through the program. When a differentiable function $g : \mathbb{R}^n \to \mathbb{R}$ is applied to a dual number with dual seed y, the dual part evaluates to the directional derivative $\langle \nabla g(x), y\rangle$. Thus, by running n-forward passes with the unit vectors as seeds, the gradient is obtained. It is further possible to employ forward mode AD for a vector function $g : \mathbb{R}^n \to \mathbb{R}^m$. Here, a single forward pass computes the Jacobian vector product $\mathbf{Jr}$ without building the Jacobian.

Implementation in this study: For computing gradients and Jacobians via forward-mode AD, `PEtab.jl` uses `ForwardDiff.jl` [33]. Note that `ForwardDiff.jl` uses multidimensional dual numbers, which can calculate multiple directional derivatives in a single forward pass. The number of derivatives computed per forward pass is referred to as the chunk size. Additionally, `ForwardDiff.jl` can compute the complete Hessian.

1.5.6 Computing $\nabla G(\boldsymbol{\theta})$ via forward sensitivity equations

For ease of notation, as the gradient is a linear operator, we derive $\nabla_p G$ for the following expression (dropping parameters not part of the ODE system and input function x):

Table 3: ODE solvers evaluated. Each ODE solver is divided into a solver category and solver family. While each solver solves the same problem, each solver family has different properties, e.g. Rosenbrock is single-step implicit, while mult-step BDF solvers are multi-step methods. Families are Rosenbrock [34], multi-step backward differentiation formula methods (BDF) [16], singly-diagonal implicit Runge-Kutta methods (SDIRK) [19], Fully-Implicit Runge-Kutta Methods (FIRK) [13], Parallelized Implicit Extrapolation Methods (PIEP) [3], Adams explicit [16], and explicit Runge Kutta. For the composite solvers, the first name denotes the non-stiff solver, and the second names the stiff solver that the composite solver should switch to upon stiffness detection.

Solver category	Solver family	Solvers
Stiff	Rosenbrock	Rosenbrock23, Rodas5P, Rodas5, Rodas4P, Rodas4
Stiff	Multi-step BDF	CVODE-BDF, QNDF, FBDF
Stiff	SDIRK	TRBDF2, Trapeziod, Kvaerno5, KenCarp4
Stiff	FIRK	RadauIIA5, RadauIIA3
Stiff	PIEP	ImplicitDeuflhardExtrapolation, ImplicitHairerWannerExtrapolation, ImplicitEulerExtrapolation
Non-stiff	Explicit Runge Kutta	Vern6, Vern7, Vern8, Vern9, BS3, BS5, DP5, DP9, Feagin14
Non-stiff	Adams explicit	CVODE-Adams, VCABM
Composite	-	Tsit5(Rosenbrock23), Vern7(Rodas5P), Vern7(Rodas4P), Vern9(Rodas5P), Vern9(Rodas4P)

$$\nabla_p G(\boldsymbol{\theta}) = \sum_{e=1}^{E}\sum_{i=1}^{N_e} \nabla_p g^{(e,i)}\Big(h^{(e,i)}\big(\mathbf{u}(\boldsymbol{\theta}_p, t^{(e,i)}\big), \sigma^{(e,i)}\big(\mathbf{u}(\boldsymbol{\theta}_p, t^{(e,i)}\big), y^{(e,i)}\Big)$$

Via the chain rule

$$\nabla_p g^{(e,i)}\Big(h^{(e,i)}\big(\mathbf{u}(\boldsymbol{\theta}_p, t^{(e,i)}\big), \sigma^{(e,i)}\big(\mathbf{u}(\boldsymbol{\theta}_p, t^{(e,i)}\big), y^{(e,i)}\Big) = \nabla_p g^{(e,i)}(\mathbf{u}, \boldsymbol{\theta}_p) + \nabla_{\mathbf{u}} g^{(e,i)}(\mathbf{u}, \boldsymbol{\theta}_p) J_p\big(\mathbf{u}(\boldsymbol{\theta}_p, t)\big)$$

where $J_p(\mathbf{u}) = \frac{\mathrm{d}\mathbf{u}}{\mathrm{d}\mathbf{p}} = \mathbf{s}$ are the sensitivities, and via the chain-rule $\nabla_p g^{(e,i)}(\mathbf{u}, \boldsymbol{\theta}_p)$ for parameter j equals (similar holds for derivative with respect to $\mathbf{u}$)

$$\nabla_p g^{(e,i)}(\mathbf{u}, \boldsymbol{\theta}_p) = \frac{\partial g^{(e,i)}}{\partial h^{(e,i)}}\frac{\partial h^{(e,i)}}{\partial \theta_{p,j}} + \frac{\partial g^{(e,i)}}{\partial \sigma^{(e,i)}}\frac{\partial \sigma^{(e,i)}}{\partial \theta_{p,j}}. \tag{4}$$

Both $\nabla_p g^{(e,i)}(\mathbf{u}, \theta_p)$ and $\nabla_u g^{(e,i)}(\mathbf{u}, \boldsymbol{\theta}_p)$ can be computed symbolically, while the sensitivities can be computed by solving the expanded ODE system;

$$\frac{\mathrm{d}\mathbf{u}}{\mathrm{d}t} = f\big(\mathbf{u}, \boldsymbol{\theta}_p\big), \qquad \mathbf{u}(t_0) = \mathbf{u}_0\big(\boldsymbol{\theta}_p\big)$$

$$\frac{\mathrm{d}}{\mathrm{d}t}\frac{\mathrm{d}\mathbf{u}}{\mathrm{d}\boldsymbol{\theta}_{p,j}} = J_{\mathbf{u}}(f)\frac{\mathrm{d}\mathbf{u}}{\mathrm{d}\boldsymbol{\theta}_{p,j}} + \frac{\partial f}{\partial \boldsymbol{\theta}_{p,j}}, \qquad \frac{\mathrm{d}\mathbf{u}}{\mathrm{d}\boldsymbol{\theta}_{p,j}}(t_0) = \frac{\mathrm{d}\mathbf{u}_0}{\mathrm{d}\boldsymbol{\theta}_{p,j}}, \qquad j = 1, \ldots, n_p.$$

Implementation in this study: `PEtab.jl` computes the sensitivities $J_p(\mathbf{u})$ via forward-mode AD (the Jacobian of the ODE solution). AMICI solves the expanded ODE system. The remaining derivatives $\nabla_p g$ and $\nabla_{\mathbf{u}} g$ are computed symbolically.

1.5.7 Computing $\nabla G(\boldsymbol{\theta})$ via adjoint sensitivity analysis

Here we derive an expression of the gradient for an experimental condition e (one forward simulation) denoted $\nabla G^{(e)}$, since the gradient is the sum of the gradients across experimental conditions $\nabla G = \sum_{e=1}^{E} G^{(e)}$ Further, for ease of notation we drop parameters not part of the ODE system. First, we introduce a Lagrangian multiplier with the same dimension as the state vector of the ODE, $\mathbf{u}$; $\boldsymbol{\lambda}(t) \in \mathbb{R}^m$, and rearrange the equation for $G^{(e)} = M^{(e)}$

$$M^{(e)} = G^{(e)} - \int_{t_0}^{T} \boldsymbol{\lambda}(t) \cdot \underbrace{\Big(\dot{\mathbf{u}}^{(e)}(\boldsymbol{\theta}_p, t) - f\big(\mathbf{u}, \boldsymbol{\theta}_p, x^{(e)}(t)\big)\Big)}_{=0} \mathrm{d}t.$$

After the rearrangement, the gradient can be expressed as

$$\nabla_p M^{(e)} = \int_{t_0}^{T} \big(\nabla_p g^{(e)} + \boldsymbol{\lambda}^*(t) \cdot J_p(f^{(e)})\big)\mathrm{d}t + \boldsymbol{\lambda}^*(t_0) \cdot J_p(\mathbf{u}(t_0)). \tag{5}$$

where $J_p(f^{(e)}) = J_p\big(f(\mathbf{u}, \boldsymbol{\theta}_p, x^e(t))\big)$ is a Jacobian, and $\boldsymbol{\lambda}$ is the solution to

$$\begin{aligned}\dot{\boldsymbol{\lambda}}(t) &= -\boldsymbol{\lambda}(t) \cdot J_{\mathbf{u}}(f^{(e)}) - \nabla_{\mathbf{u}} g \\ \boldsymbol{\lambda}(T) &= \mathbf{0}\end{aligned}$$

where $J_{\mathbf{u}}(f^{(e)}) = J_{\mathbf{u}}\big(f(\mathbf{u}, \boldsymbol{\theta}_p, x^e(t))\big)$ is the Jacobian of the right-hand side of the ODE model. The sensitivities at time zero, $J_p(\mathbf{u}_{t0}^{(e)})$, can be computed without solving the ODE system, and $\nabla_{\mathbf{u}} g^{(e)}$ and $\nabla_p g^{(e)}$ have closed forms which can be computed symbolically (see above for forward sensitivities). The gradient can be computed by solving the ODE system for $\boldsymbol{\lambda}$, and then by evaluating the integral in Eq. (5). It should be noted that since we only observe data at discrete time-points, $t_1, \ldots, t_{N_e}$ when solving for $\boldsymbol{\lambda}$ the term $-\nabla_{\mathbf{u}} g^{(e)}$ is applied as a jump (discrete event) at the observed time-points. Further, following the PEtab standard g is time-point i dependent; $g^{(e,i)}$.

Implementation in this study: `PEtab.jl` can use either the quadrature and interpolation approach in `SciMLSensitivity.jl` [29, 24]. The vector-Jacobian-product $\boldsymbol{\lambda}^* \cdot J_p(f^{(e)})$ is computed in a Jacobian-free manner either via the Enzyme [27], or `ReverseDiff.jl` automatic differentiation libraries. AMICI employs the Sundial's adjoint implementation [8], which is similar to the interpolation in `SciMLSensitivity.jl` [29]. Specifically, both approaches utilize an interpolation of $\mathbf{u}(t)$ to solve for $\boldsymbol{\lambda}(t)$, and thus do not store the entire trajectory of $\boldsymbol{\lambda}(t)$ over time. Both approaches employ a similar checkpointing strategy where the chosen interval points come from the forward solution, and when the reverse pass enters a new interval, the original ODE is re-solved on the interval $[t_{k-1}, t_k]$. It should also be noted that in AMICI, the Jacobian is computed symbolically, and the product is computed via sparse vector-matrix multiplication.

1.5.8 Hessian computations

- BFGS. A Quasi-Newton method approximating the Hessian with a positive-definite matrix B updated iteratively from previous computation steps assumes $H(x_{k+1}) \approx H(x_k)$. Since the computation doesn't require second-order sensitivities, the computational complexity is substantially reduced for large models. Let f be a twice differentiable (objective) function $f\colon \mathbb{R}^n \to \mathbb{R}$. Note that the update scheme is specific to each Quasi-Newton method, but B needs to fulfil the Quasi-Newton condition in any case: $B_{k+1}[x_{k+1} - x_k] = \nabla f(x_{k+1}) - f(x_k)$. The update for the Broyden-Fletcher-Goldfarb-Shannon (BFGS) algorithm is given by
$$B_{k+1} = B_k + \frac{y_k y_k^T}{y_k^T \Delta x_k} - \frac{B_k \Delta x_k \Delta x_k^T B_k}{\Delta x_k^T B_k \Delta x_k}.$$
- Gauss-Newton. Similarly, the Gauss-Newton method avoids the calculation of second derivatives as well by recasting the Hessian computation as a non-linear least squares problem: $\min_{x \in \mathbf{R}^n} \Big(\frac{1}{2}||f(x)||^2 = \frac{1}{2}||J(x - x^0) + f(x^0)||^2\Big)$. By linearization of the objective function, f through Taylor series expansion of order one and using the Jacobian matrix J, the Hessian (after some calculus) can be approximated by

$J(x) + H(x)\Delta x = 0$ avoiding computing and inverting a potentially large Hessian matrix as a limiting factor. The Gauss-Newton matrix approximating the Hessian H is given by:

$$G_{ij} = \sum_{l=0}^{n} \frac{\partial f_l(x)}{\partial x_j} \sum_{k=0}^{n} \frac{\partial f_k(x)}{\partial x_i} \frac{\partial \sigma^2}{\partial f_l(x) f_k(x)} (f(x)).$$

1.5.9 Robustness of results

All Julia benchmarks were performed using Julia version 1.8.5, except the stochastic ones, which (due to being carried out at a later stage) were performed on version 1.10.2 [2]. The `pyPESTO` and AMICI benchmarks were performed using Python version 3.10.6, Amici version 0.17 to address bugs for Smith and Schwen models [8] and version 0.15 for remaining benchmarks, and `pyPESTO` version 0.2.15 [35]. The code used to perform all benchmarks can be found at `https://github.com/cvijoviclab/PEtab_benchmark`, and on Zenodo at `https://doi.org/10.5281/zenodo.16895370`. This repository also provides a comprehensive guide on how to set up the necessary environment for running the benchmarks. It should be noted that for the benchmarks, we used a predecessor version of `PEtab.jl`, which was later migrated to a new repository to create the formal Julia package repository. This holds for all models, except Bruno, where due to a bug in the `PEtab.jl` predecessor version found during revision, we ran the benchmark with `PEtab.jl` version 2.14. Complementary, identical benchmarks have been run on a second compute infrastructure to demonstrate the robustness and reproducibility of results corresponding to the Julia benchmarks with Julia version 1.9.0 respectively, `pyPESTO` and AMICI benchmarks with Python 3.10.4, AMICI version 0.15 and `pyPESTO` 0.3.0.

1.5.10 Disclosure - usage of AI-assisted tools

We used ChatGTP4 solely for text editing to improve the readability of the manuscript. Specifically, once the first draft, including references, had been completed, for each paragraph, we ran the ChatGTP4 with the prompt: *Please improve the readability of this paragraph.* Subsequently, the text produced by ChatGTP4 was edited to avoid any potential inaccuracies and misinterpretations or to avoid unclear formulations. To further minimise any chance for potential inaccuracies from the language model, ChatGTP4 was applied prior to circulating the manuscript between all the authors. Taken together, the authors reviewed and edited the content as needed and took full responsibility for the content of the publication.

2 Extended discussion on benchmark results

This section contains extended discussion on a subset of benchmark results.

2.1 Extended discussion on ODE solvers

In the benchmark evaluating a selection of ODE solvers on smaller models with randomly chosen parameters (Fig. 2 and S1), for a subset of models and parameters, the ODE solvers encountered simulation failure. To investigate why, we investigated the ODE solver return codes (Fig. 9a). The non-stiff, explicit ODE solvers (Vern7 and Tsit5) primarily failed with code *Maxiters*, which is common when using a non-stiff solver on a stiff problem.

Regarding the multi-step BDF solvers QNDF and CVODE_BDF, the former failed more frequently (Fig. 2). The most common error code for QNDF was *DtLessThanMin* which means it fails to meet integration tolerances, and as this happened for times close to zero the results suggests it fails during the startup phase where it relies on lower-order methods. For a subset of SIR models (Bertozzi and Giordano) both solvers had code *Maxiters*, which can be fixed by reducing solver tolerances (Fig. 9b), and probably by setting larger maximum iteration number. Overall, given that CVODE_BDF and QNDF have comparable runtimes but CVODE_BDF is more reliable (Fig. 2), and there are models (e.g. Schwen) where lowering tolerances for QNDF do not make it able to simulate for all vectors that CVODE_BDF can, for simulation-based tasks with BDF solvers we recommend CVODE_BDF. Nevertheless, in cases where QNDF performs well (Bachmann Fig. 2 and Fig. 3), as it is compatible with efficient automatic differentiation (Fig. 4), for gradient-based tasks, QNDF can be a good choice.

The composite solver Vern7Rodas5P failed more frequently for stiffer models (Schwen, Bachmann, Weber, and Fiedler) than CVODE_BDF, often with error code *Unstable*. This suggests the composite solver does not switch to the stiff solver in time for these models, and in addition, for these models (except Weber), lowering the tolerance did not make it able to simulate the model. Conversely, for models with less apparent stiffness, like the SIR models (Bertozzi, Zhao, Okunghae, and Giordano), Vern7Rodas5P was the fastest solver.

The Rosenbrock solver Rodas5P was reliable, and it only had more simulation failures than CVODE_BDF for two models (Borghans 80 vs 77, Fiedler 8 vs 0). It had the *unstable* error code for both the Fiedler and Giordano models, but for the latter, it could simulate the model with lower tolerances (Fig. 9). Overall, as Rodas5P generally is more accurate than BDF methods (Fig. 8a) and often is faster for models with less than 25 ODEs (Fig. 2), we recommend Rosenbrock solvers like Rodas5P for smaller models. Moreover, the Fiedler model was the only case where Rodas5P had problems compared to other solvers, but the results suggest that in cases where Rosenbrock solvers often have recode *Unstable*, switching to a BDF-solver can help.

Lastly, Borghans (a spiking model) and Crauste (a virus growth model) had many simulation failures. Error codes indicate unstable dynamics for a subset of parameters, and reducing tolerances often failed to resolve the issue. Importantly, the composite solver had a few error-codes of *Longtime* (solver was aborted as it took $>$ 15min), potentially because it diverged so badly it ended in a part of model phase-space where solving linear systems takes considerable time. Regardless, this suggests that for numerically challenging models avoid composite solvers as they can put workflows to a halt with long simulation times, and even though stiff solvers performed better here, there is no clear best choice.

In a flow-chart (Fig. 6), we summarise the result and make the following recommendations: for less stiff models, such as many SIR models, composite solvers, for smaller, stiffer models, like molecular models, Rosenbrock solvers like Rodas5P. Should Rosenbrock solvers have problems (e.g. Fiedler model), switching to a BDF method can help. For larger, stiffer models, BDF solvers are suitable. If issues occur with QNDF, CVODE_BDF is reliable. For certain large models like those with many events, SDIRK solvers like KenCarp4 should also be considered (Fig. 3).

2.2 Extended discussion on parameter estimation results

In the parameter estimation benchmark results for the Fides optimiser with the Gauss-Newton Hessian approximation for a subset of models (e.g. Crauste and Isensee) `pyPESTO` and `PEtab.jl` have a noticeable difference in the number of converged runs (Fig. 7). As both pyPESTO and PEtab.jl are thoroughly tested (further confirmed by comparing derivatives between software), the variance in converged runs is likely because of it it being hard to accurately compute the Gauss-Newton Hessian approximation for a subset of numerically challenging models. This is further supported by looking closer at the Isensee and Crauste models where number

of converged runs differ, compared to the Boehm model where both software have similar number of converged runs (Tab. 4).

The Isensee model is large, stiff, and has a pre-equilibration condition (steady-state simulations). Just like for the other pre-equilibration models (Zheng, Weber and Brannmark), neither software had many converged runs. Moreover, for the Isensee model AMICI's sophisticated approach for steady-state handling, which for example was used used for the Zheng model, cannot be employed as the model has a singular Jacobian. Overall, the stiffness, size and pre-equilibration makes it challenging to compute derivatives. For example, the difference in ℓ_2-norm between a high accuracy gradient and Gauss-Newton Hessian (obtained low solver tolerances), is larger than for the Boehm model (Tab. 4). Further, the differences are larger for the Gauss-Newton Hessian than the gradient (Tab. 4). Naturally, one action to obtain more accurate gradients in practice could be to lower ODE solver tolerances, however, this also increases the number of simulation failures [38].

The Crauste model is even more numerically challenging than the Isensee model. Lowering ODE solver tolerances results in drastic differences for both the gradient and Guass-Newton Hessian approximation compared to the high accuracy gradient reference. The difference is larger for the Gauss-Newton Hessian (Tab. 4).

In summary, the variance in converged when running parameter estimation using Fides with the Gauss-Newton Hessian approximation for a subset of models (e.g. Crauste and Isensee), is likely because computing gradients, and particular a Hessian approximation is challenging for a subset of models. Hence, using different gradient methods and ODE solvers likely affects the results. Given that neither `PEtab.jl` nor `pyPESTO` consistently shows higher convergence rates across models, neither software with respect to this aspect cannot really be recommended over the other, and identifying exactly why for example `PEtab.jl` performed better for Iseense is an interesting future research direction. Lastly, the difference in converged runs is smaller when running Fides with BFGS Hessian approximation, likely because BFGS uses gradients across the history for a robust estimate Hessian estimate, and it only uses the gradient from PEtab.jl and pyPESTO.

References

[1] Atilim Gunes Baydin, Barak A Pearlmutter, Alexey Andreyevich Radul, and Jeffrey Mark Siskind. Automatic differentiation in machine learning: a survey. Journal of Marchine Learning Research, 18:1–43, 2018.

[2] Jeff Bezanson, Alan Edelman, Stefan Karpinski, and Viral B Shah. Julia: A fresh approach to numerical computing. SIAM review, 59(1):65–98, 2017.

[3] Chris Elrod, Yingbo Ma, Konstantin Althaus, Christopher Rackauckas, et al. Parallelizing explicit and implicit extrapolation methods for ordinary differential equations. In 2022 IEEE High Performance Extreme Computing Conference (HPEC), pages 1–9. IEEE, 2022.

[4] F. Fröhlich, F. J. Theis, J. O. Rädler, and J. Hasenauer. Parameter estimation for dynamical systems with discrete events and logical operations. Bioinformatics, 33(7):1049–1056, 2017.

[5] Fabian Fröhlich, Barbara Kaltenbacher, Fabian J Theis, and Jan Hasenauer. Scalable parameter estimation for genome-scale biochemical reaction networks. PLoS computational biology, 13(1):e1005331, 2017.

[6] Fabian Fröhlich, Thomas Kessler, Daniel Weindl, Alexey Shadrin, Leonard Schmiester, Hendrik Hache, Artur Muradyan, Moritz Schütte, Ji-Hyun Lim, Matthias Heinig, et al. Efficient parameter estimation enables the prediction of drug response using a mechanistic pan-cancer pathway model. Cell systems, 7(6):567–579, 2018.

[7] Fabian Fröhlich, Carolin Loos, and Jan Hasenauer. Scalable inference of ordinary differential equation models of biochemical processes. Gene Regulatory Networks: Methods and Protocols, pages 385–422, 2019.

[8] Fabian Fröhlich and Peter K Sorger. Fides: Reliable trust-region optimization for parameter estimation of ordinary differential equation models. PLOS Computational Biology, 18(7):e1010322, 2022.

[9] Fabian Fröhlich, Daniel Weindl, Yannik Schälte, Dilan Pathirana, Łukasz Paszkowski, Glenn Terje Lines, Paul Stapor, and Jan Hasenauer. Amici: high-performance sensitivity analysis for large ordinary differential equation models. Bioinformatics, 37(20):3676–3677, 2021.

[10] Daniel T Gillespie. A general method for numerically simulating the stochastic time evolution of coupled chemical reactions. Journal of Computational Physics, 22(4):403–434, December 1976.

[11] Daniel T. Gillespie. Exact stochastic simulation of coupled chemical reactions. The Journal of Physical Chemistry, 81(25):2340–2361, December 1977. Publisher: American Chemical Society.

[12] Abhishekh Gupta and Pedro Mendes. An Overview of Network-Based and -Free Approaches for Stochastic Simulation of Biochemical Systems. Computation (Basel), 6(1), 2018.

[13] Ernst Hairer and Gerhard Wanner. Stiff differential equations solved by radau methods. Journal of Computational and Applied Mathematics, 111(1-2):93–111, 1999.

[14] Helge Hass, Carolin Loos, Elba Raimúndez-Álvarez, Jens Timmer, Jan Hasenauer, and Clemens Kreutz. Benchmark problems for dynamic modeling of intracellular processes. Bioinformatics, 35(17):3073–3082, 2019.

[15] Alan C Hindmarsh, Peter N Brown, Keith E Grant, Steven L Lee, Radu Serban, Dan E Shumaker, and Carol S Woodward. Sundials: Suite of nonlinear and differential/algebraic equation solvers. ACM Transactions on Mathematical Software (TOMS), 31(3):363–396, 2005.

[16] Alan C Hindmarsh, Peter N Brown, Keith E Grant, Steven L Lee, Radu Serban, Dan E Shumaker, and Carol S Woodward. SUNDIALS: Suite of nonlinear and differential/algebraic equation solvers. ACM Transactions on Mathematical Software (TOMS), 31(3):363–396, 2005.

[17] S. A. Isaacson. ReactionNetworkImporters.jl. `https://github.com/SciML/ReactionNetworkImporters.jl`, 2022.

[18] S. A. Isaacson, V. Ilin, and C. V. Rackauckas. JumpProcesses.jl. `https://github.com/SciML/JumpProcesses.jl/`, 2022.

[19] Anne Kværnø. Singly diagonally implicit runge–kutta methods with an explicit first stage. BIT Numerical Mathematics, 44:489–502, 2004.

[20] Polina Lakrisenko, Paul Stapor, Stephan Grein, Łukasz Paszkowski, Dilan Pathirana, Fabian Fröhlich, Glenn Terje Lines, Daniel Weindl, and Jan Hasenauer. Efficient computation of adjoint sensitivities at steady-state in ode models of biochemical reaction networks. PLOS Computational Biology, 19(1):e1010783, 2023.

[21] Paola Lecca. Stochastic chemical kinetics. Biophysical Reviews, 5(4):323–345, December 2013.

[22] Torkel E Loman, Yingbo Ma, Vasily Ilin, Shashi Gowda, Niklas Korsbo, Nikhil Yewale, Chris Rackauckas, and Samuel A Isaacson. Catalyst: Fast and flexible modeling of reaction networks. PLOS Computational Biology, 19(10):e1011530, 2023.

[23] Carlos F Lopez, Jeremy L Muhlich, John A Bachman, and Peter K Sorger. Programming biological models in python using pysb. Molecular systems biology, 9(1):646, 2013.

[24] Yingbo Ma, Vaibhav Dixit, Michael J Innes, Xingjian Guo, and Chris Rackauckas. A comparison of automatic differentiation and continuous sensitivity analysis for derivatives of differential equation solutions. In 2021 IEEE High Performance Extreme Computing Conference (HPEC), pages 1–9. IEEE, 2021.

[25] J. M. McCollum, G. D. Peterson, C. D. Cox, M. L. Simpson, and N. F. Samatova. The sorting direct method for stochastic simulation of biochemical systems with varying reaction execution behavior. Computational Biology and Chemistry, 30(1), 2006.

[26] Patrick Kofod Mogensen and Asbjørn Nilsen Riseth. Optim: A mathematical optimization package for Julia. Journal of Open Source Software, 3(24):615, 2018.

[27] William Moses and Valentin Churavy. Instead of rewriting foreign code for machine learning, automatically synthesize fast gradients. In H. Larochelle, M. Ranzato, R. Hadsell, M. F. Balcan, and H. Lin, editors, Advances in Neural Information Processing Systems, volume 33, pages 12472–12485. Curran Associates, Inc., 2020.

[28] Jake Alan Pitt and Julio R Banga. Parameter estimation in models of biological oscillators: an automated regularised estimation approach. BMC bioinformatics, 20:1–17, 2019.

[29] Christopher Rackauckas, Yingbo Ma, Julius Martensen, Collin Warner, Kirill Zubov, Rohit Supekar, Dominic Skinner, Ali Ramadhan, and Alan Edelman. Universal differential equations for scientific machine learning. arXiv preprint arXiv:2001.04385, 2020.

[30] Christopher Rackauckas and Qing Nie. DifferentialEquations.jl–a performant and feature-rich ecosystem for solving differential equations in Julia. Journal of Open Research Software, 5(1), 2017.

[31] Andreas Raue, Marcel Schilling, Julie Bachmann, Andrew Matteson, Max Schelke, Daniel Kaschek, Sabine Hug, Clemens Kreutz, Brian D Harms, Fabian J Theis, et al. Lessons learned from quantitative dynamical modeling in systems biology. PloS one, 8(9):e74335, 2013.

[32] Andreas Raue, Bernhard Steiert, Max Schelker, Clemens Kreutz, Tim Maiwald, Helge Hass, Joep Vanlier, Christian Tönsing, Lorenz Adlung, Raphael Engesser, et al. Data2dynamics: a modeling environment tailored to parameter estimation in dynamical systems. Bioinformatics, 31(21):3558–3560, 2015.

[33] Jarrett Revels, Miles Lubin, and Theodore Papamarkou. Forward-mode automatic differentiation in julia. arXiv preprint arXiv:1607.07892, 2016.

[34] HH Rosenbrock. Some general implicit processes for the numerical solution of differential equations. The Computer Journal, 5(4):329–330, 1963.

[35] Yannik Schälte, Fabian Fröhlich, Paul J Jost, Jakob Vanhoefer, Dilan Pathirana, Paul Stapor, Polina Lakrisenko, Dantong Wang, Elba Raimúndez, Simon Merkt, et al. pypesto: A modular and scalable tool for parameter estimation for dynamic models. arXiv preprint arXiv:2305.01821, 2023.

[36] Leonard Schmiester, Yannik Schälte, Frank T Bergmann, Tacio Camba, Erika Dudkin, Janine Egert, Fabian Fröhlich, Lara Fuhrmann, Adrian L Hauber, Svenja Kemmer, et al. Petab—interoperable specification of parameter estimation problems in systems biology. PLoS computational biology, 17(1):e1008646, 2021.

[37] Michael W Sneddon, James R Faeder, and Thierry Emonet. Efficient modeling, simulation and coarse-graining of biological complexity with nfsim. Nature methods, 8(2):177–183, 2011.

[38] Philipp Städter, Yannik Schälte, Leonard Schmiester, Jan Hasenauer, and Paul L Stapor. Benchmarking of numerical integration methods for ode models of biological systems. Scientific Reports, 11(1):1–11, 2021.

[39] Vo Hong Thanh, Corrado Priami, and Roberto Zunino. Efficient rejection-based simulation of biochemical reactions with stochastic noise and delays. The Journal of Chemical Physics, 141(13):134116–134113, 2014.

[40] Vo Hong Thanh, Roberto Zunino, and Corrado Priami. On the rejection-based algorithm for simulation and analysis of large-scale reaction networks. The Journal of Chemical Physics, 142(24):244106–244114, 2015.

[41] Vo Hong Thanh, Roberto Zunino, and Corrado Priami. Efficient constant-time complexity algorithm for stochastic simulation of large reaction networks. IEEE/ACM Transactions on Computational Biology and Bioinformatics, 14(3):657–667, 2017.

[42] Alejandro F Villaverde, Fabian Fröhlich, Daniel Weindl, Jan Hasenauer, and Julio R Banga. Benchmarking optimization methods for parameter estimation in large kinetic models. Bioinformatics, 35(5):830–838, 2019.

[43] Ciaran Welsh, Jin Xu, Lucian Smith, Matthias König, Kiri Choi, and Herbert M Sauro. libroadrunner 2.0: a high performance sbml simulation and analysis library. Bioinformatics, 39(1):btac770, 2023.

[44] Guilherme Augusto Zagatti, Samuel A. Isaacson, Christopher Rackauckas, Vasily Ilin, See-Kiong Ng, and Stéphane Bressan. Extending JumpProcess.jl for fast point process simulation with time-varying intensities, July 2023. arXiv:2306.06992 [cs, stat].

Additional tables

Table 4: Effect of changing ODE solver tolerance on gradient and Gauss-Newton Hessian (H) accuracy. For Crauste and Boehm the reference gradient and Hessian were computed using tolerance = 10^{-12}, while due to simulation failure the Isensee the tolerance 10^{-11} was used. The ℓ_2 columns are the difference in ℓ_2 norm compared to the reference, and the max-diff columns are the maximum difference in an element compared to the reference.

Model	Solver tolerance	ℓ_2-norm grad.	ℓ_2-norm H.	Max. grad. diff	Max. H diff
Crauste	10^{-6}	5.9×10^{4}	4.8×10^{11}	4.2×10^{4}	2.5×10^{11}
Crauste	10^{-8}	492	3.9×10^{9}	350	2×10^{9}
Crauste	10^{-10}	5.6	4.6×10^{7}	4	2.3×10^{7}
Boehm	10^{-6}	4.6×10^{-5}	1.9×10^{-4}	3.7×10^{-5}	7.9×10^{-5}
Boehm	10^{-8}	1.1×10^{-6}	4.4×10^{-6}	8.7×10^{-7}	1.8×10^{-8}
Boehm	10^{-10}	1.7×10^{-8}	1.4×10^{-8}	1.7×10^{-8}	2.2×10^{-8}
Isensee	10^{-6}	1.5	426	1.18	173
Isensee	10^{-8}	0.008	2.0	0.006	0.81
Isensee	10^{-10}	7.5×10^{-5}	0.01	4.4×10^{-5}	0.004

Table 5: SBML component and test tags supported by SBMLImporter.jl. For comparison, AMICI is also included. Tags apply to the semantic test suite. Only tags where either tool passes one test with the tag are reported in the table. To date, AMICI passes 1247 and SBMLImporter.jl 1257 of the 1821 test cases. The main differences boil down to AMICI supporting hierarchical models, while SBMLImporter.jl supports StoichiometryMath.

Tag	Tag type	Supported AMICI	Supported SBMLImporter.jl
AlgebraicRule	Component tag	×	×
AssignmentRule	Component tag	×	×
comp	Component tag	×	
Compartment	Component tag	×	×
CSymbolAvogadro	Component tag	×	×
CSymbolRateOf	Component tag	×	×
CSymbolTime	Component tag	×	×
Deletion	Component tag	×	×
EventNoDelay	Component tag	×	×
ExternalModelDefinition	Component tag	×	
FunctionDefinition	Component tag	×	×
InitialAssignment	Component tag	×	×
ModelDefinition	Component tag	×	
Parameter	Component tag	×	×
Port	Component tag	×	
RateRule	Component tag	×	×
Reaction	Component tag	×	×
ReplacedBy	Component tag	×	
ReplacedElement	Component tag	×	
SBaseRef	Component tag	×	
Species	Component tag	×	×
StoichiometryMath	Component tag		×
Submodel	Component tag	×	
0D-Compartment	Test tag	×	×
Amount	Test tag	×	×
AssignedConstantStoichiometry	Test tag	×	×
AssignedVariableStoichiometry	Test tag	×	×
BoolNumericSwap	Test tag	×	×
BoundaryCondition	Test tag	×	×
comp	Test tag	×	
Concentration	Test tag	×	×
ConstantSpecies	Test tag	×	×
ConversionFactors	Test tag	×	×
DefaultValue	Test tag	×	×
EventT0Firing	Test tag	×	×
ExtentConversionFactor	Test tag	×	
HasOnlySubstanceUnits	Test tag	×	×
InitialValueReassigned	Test tag	×	×
L3v2MathML	Test tag	×	×
LocalParameters	Test tag	×	×
MultiCompartment	Test tag	×	×
NoMathML	Test tag	×	×
NonConstantCompartment	Test tag	×	×
NonConstantParameter	Test tag	×	×
NonUnityCompartment	Test tag	×	×
NonUnityStoichiometry	Test tag	×	×
ReversibleReaction	Test tag	×	×
SpeciesReferenceInMath	Test tag	×	×
SubmodelOutput	Test tag	×	
TimeConversionFactor	Test tag	×	
UncommonMathML	Test tag	×	×
VolumeConcentrationRates	Test tag	×	×

Table 6: Runtime evaluation for the cost (objective) and gradient. Benchmark is performed at reported values. AMICI uses CVODE-BDF, while the Julia PEtab importer uses the Rodas5P and QNDF ODE solvers. Ratio represents the ratio between the runtime for the gradient and cost, and Inf denotes gradient computational failure. The number of model parameters the gradient is taken to is given in the rightmost column.

Solver	Time cost [s]	Time gradient [s]	Ratio	Number of parameters
Zheng_PNAS2012				
QNDF	7.05e-04	3.82e-02	5.41e+01	46
Rodas5P	6.90e-04	4.93e-02	7.14e+01	46
AMICI	2.84e-04	1.43e-02	5.03e+01	46
Weber_BMC2015				
QNDF	1.48e-03	2.04e-02	1.38e+01	36
Rodas5P	2.55e-03	2.97e-02	1.16e+01	36
AMICI	1.15e-03	6.19e-02	5.36e+01	36
Sneyd_PNAS2002				
QNDF	2.84e-03	1.83e-02	6.47e+00	15
Rodas5P	2.38e-03	1.85e-02	7.77e+00	15
AMICI	2.47e-03	4.82e-02	1.95e+01	15
Schwen_PONE2014				
QNDF	7.98e-03	6.11e-02	7.65e+00	30
Rodas5P	8.81e-03	7.25e-02	8.23e+00	30
AMICI	5.72e-03	1.43e-01	2.51e+01	30
Rahman_MBS2016				
QNDF	2.75e-04	1.02e-03	3.71e+00	9
Rodas5P	4.62e-04	1.48e-03	3.21e+00	9
AMICI	2.95e-04	5.33e-03	1.81e+01	9
Oliveira_NatCommun2021				
QNDF	1.06e-03	Inf	Inf	12
Rodas5P	5.65e-04	4.42e-03	7.82e+00	12
AMICI	7.53e-04	8.25e-03	1.10e+01	12
Okuonghae_ChaosSolitonsFractals2020				
QNDF	3.22e-04	1.64e-03	5.10e+00	16
Rodas5P	2.11e-04	1.52e-03	7.23e+00	16
AMICI	3.13e-04	5.83e-03	1.86e+01	16
Lucarelli_CellSystems2018				
QNDF	1.44e-02	2.28e+00	1.59e+02	84
Rodas5P	4.58e-02	1.04e+01	2.26e+02	84
AMICI	1.02e-02	1.16e+00	1.13e+02	84
Isensee_JCB2018				
QNDF	1.02e-01	2.94e+00	2.87e+01	46
Rodas5P	1.07e-01	3.74e+00	3.51e+01	46
AMICI	6.09e-02	2.99e+00	4.91e+01	46
Fujita_SciSignal2010				
Rodas5P	3.06e-03	2.32e-02	7.59e+00	19
AMICI	3.26e-03	1.28e-01	3.92e+01	19
Fiedler_BMC2016				
QNDF	7.36e-04	4.40e-03	5.98e+00	22
Rodas5P	6.77e-04	6.07e-03	8.97e+00	22

AMICI	7.79e-04	2.29e-02	2.94e+01	22
Elowitz_Nature2000				
QNDF	2.59e-03	1.91e-02	7.38e+00	21
Rodas5P	3.66e-03	2.73e-02	7.46e+00	21
AMICI	1.61e-03	4.04e-02	2.50e+01	21
Crauste_CellSystems2017				
QNDF	8.97e-04	5.26e-03	5.86e+00	12
Rodas5P	8.53e-04	6.87e-03	8.06e+00	12
AMICI	6.48e-04	1.40e-02	2.16e+01	12
Bruno_JExpBot2016				
QNDF	5.02e-04	3.22e-03	6.41e+00	13
Rodas5P	3.56e-04	2.32e-03	6.50e+00	13
AMICI	7.13e-04	6.89e-03	9.67e+00	13
Brannmark_JBC2010				
QNDF	2.75e-03	2.23e-02	8.11e+00	22
Rodas5P	2.98e-03	2.48e-02	8.33e+00	22
AMICI	3.35e-03	8.14e-02	2.43e+01	22
Borghans_BiophysChem1997				
QNDF	6.94e-03	5.88e-02	8.47e+00	23
Rodas5P	1.32e-02	1.23e-01	9.29e+00	23
AMICI	2.31e-03	7.11e-02	3.08e+01	23
Boehm_JProteomeRes2014				
QNDF	5.69e-04	1.74e-03	3.06e+00	9
Rodas5P	5.88e-04	1.94e-03	3.30e+00	9
AMICI	4.46e-04	4.45e-03	9.98e+00	9
Beer_MolBioSystems2014				
QNDF	1.07e-02	8.95e-04	8.33e-02	72
Rodas5P	1.09e-02	3.04e-01	2.78e+01	72
AMICI	2.22e-02	1.79e-01	8.05e+00	72
Bachmann_MSB2011				
QNDF	3.22e-02	7.46e-01	2.32e+01	113
Rodas5P	5.48e-02	1.54e+00	2.81e+01	113
AMICI	1.83e-02	1.08e+00	5.92e+01	113

Table 7: Runtime evaluation for the cost (objective) and Hessian. Benchmark is performed at reported values. Julia PEtab importer uses the Rodas5P and QNDF ODE solvers. The ratio represents the ratio between the runtime for the Hessian and cost, and Inf denotes Hessian computation failure. The number of model parameters the Hessian is taken to is given in the rightmost column. Note the Hessian is computed with a forward-over-forward approach and thus should theoretically have a quadratic scaling with the number of parameters.

Solver	Time Hessian [s]	Time cost [s]	Ratio	Number of parameters
Zheng_PNAS2012				
QNDF	3.63e+00	8.92e-04	4.07e+03	46
Rodas5P	5.90e+00	8.60e-04	6.87e+03	46
Weber_BMC2015				
QNDF	1.98e+00	1.82e-03	1.09e+03	36
Rodas5P	5.89e+00	2.85e-03	2.07e+03	36
Sneyd_PNAS2002				
QNDF	9.48e-01	3.48e-03	2.73e+02	15
Rodas5P	1.45e+00	2.54e-03	5.69e+02	15
Schwen_PONE2014				
QNDF	3.70e+00	7.93e-03	4.66e+02	30
Rodas5P	5.55e+00	8.64e-03	6.42e+02	30
Rahman_MBS2016				
QNDF	1.42e-02	2.71e-04	5.22e+01	9
Rodas5P	4.85e-02	3.38e-04	1.44e+02	9
Oliveira_NatCommun2021				
QNDF	1.40e-03	Inf	Inf	12
Rodas5P	7.42e-02	5.68e-04	1.31e+02	12
Okuonghae_ChaosSolitonsFractals2020				
QNDF	2.78e-02	1.96e-04	1.42e+02	16
Rodas5P	3.89e-02	1.64e-04	2.37e+02	16
Lucarelli_CellSystems2018				
QNDF	3.30e+02	2.04e-02	1.62e+04	84
Rodas5P	1.50e+03	6.08e-02	2.47e+04	84
Isensee_JCB2018				
Rodas5P	Inf	1.37e-01	Inf	46
Fujita_SciSignal2010				
QNDF	9.62e-01	4.58e-03	2.10e+02	19
Rodas5P	1.05e+00	3.37e-03	3.13e+02	19
Fiedler_BMC2016				
QNDF	2.35e-01	9.11e-04	2.58e+02	22
Rodas5P	4.11e-01	8.86e-04	4.64e+02	22
Elowitz_Nature2000				
QNDF	5.92e-01	2.72e-03	2.18e+02	21
Rodas5P	1.00e+00	3.91e-03	2.57e+02	21
Crauste_CellSystems2017				
QNDF	1.11e-01	1.14e-03	9.79e+01	12
Rodas5P	1.84e-01	9.79e-04	1.88e+02	12

Bruno_JExpBot2016				
QNDF	5.69e-02	6.14e-04	9.26e+01	13
Rodas5P	5.34e-02	3.77e-04	1.41e+02	13
Brannmark_JBC2010				
QNDF	1.08e+00	3.33e-03	3.25e+02	22
Rodas5P	1.88e+00	3.23e-03	5.80e+02	22
Borghans_BiophysChem1997				
QNDF	1.92e+00	6.79e-03	2.82e+02	23
Rodas5P	5.42e+00	1.30e-02	4.19e+02	23
Boehm_JProteomeRes2014				
QNDF	2.45e-02	6.69e-04	3.66e+01	9
Rodas5P	4.09e-02	6.93e-04	5.90e+01	9
Beer_MolBioSystems2014				
Rodas5P	3.31e+01	1.45e-02	2.29e+03	72
Bachmann_MSB2011				
QNDF	9.46e+02	3.86e-02	2.45e+04	113
Rodas5P	2.44e+03	7.05e-02	3.46e+04	113

Additional figures

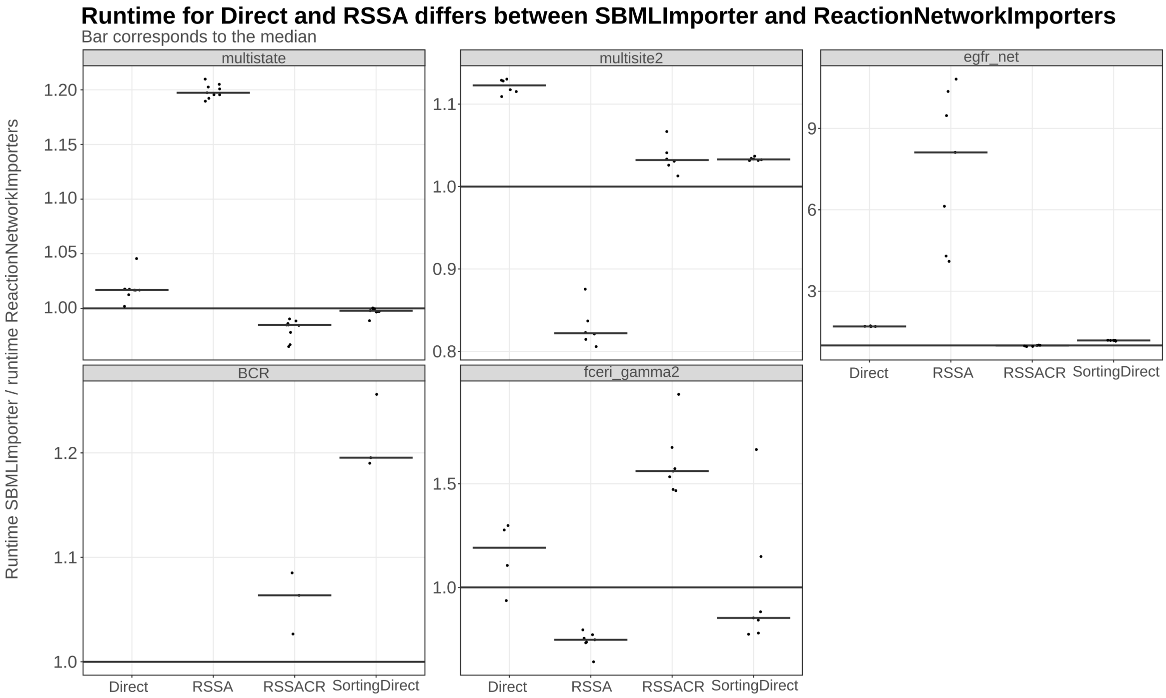

Figure 7: Benchmark of stochastic simulators: Simulation runtime for models imported as SBML files using `SBMLImporter.jl` divided by simulation runtime when the model is imported as .net files via `ReactionNetworkImporters.jl`, for the same simulation intervals as in Fig. 2. The order of reactions differs in the parsed Catalyst reaction network differs between the importers, which notably impacted runtime for Direct and RSSA methods; for instance, RSSA was about 1.2 times faster when using .net file imports for the multistate model. Before benchmarking, it was verified that the reaction and model parameters were identical between the importers.

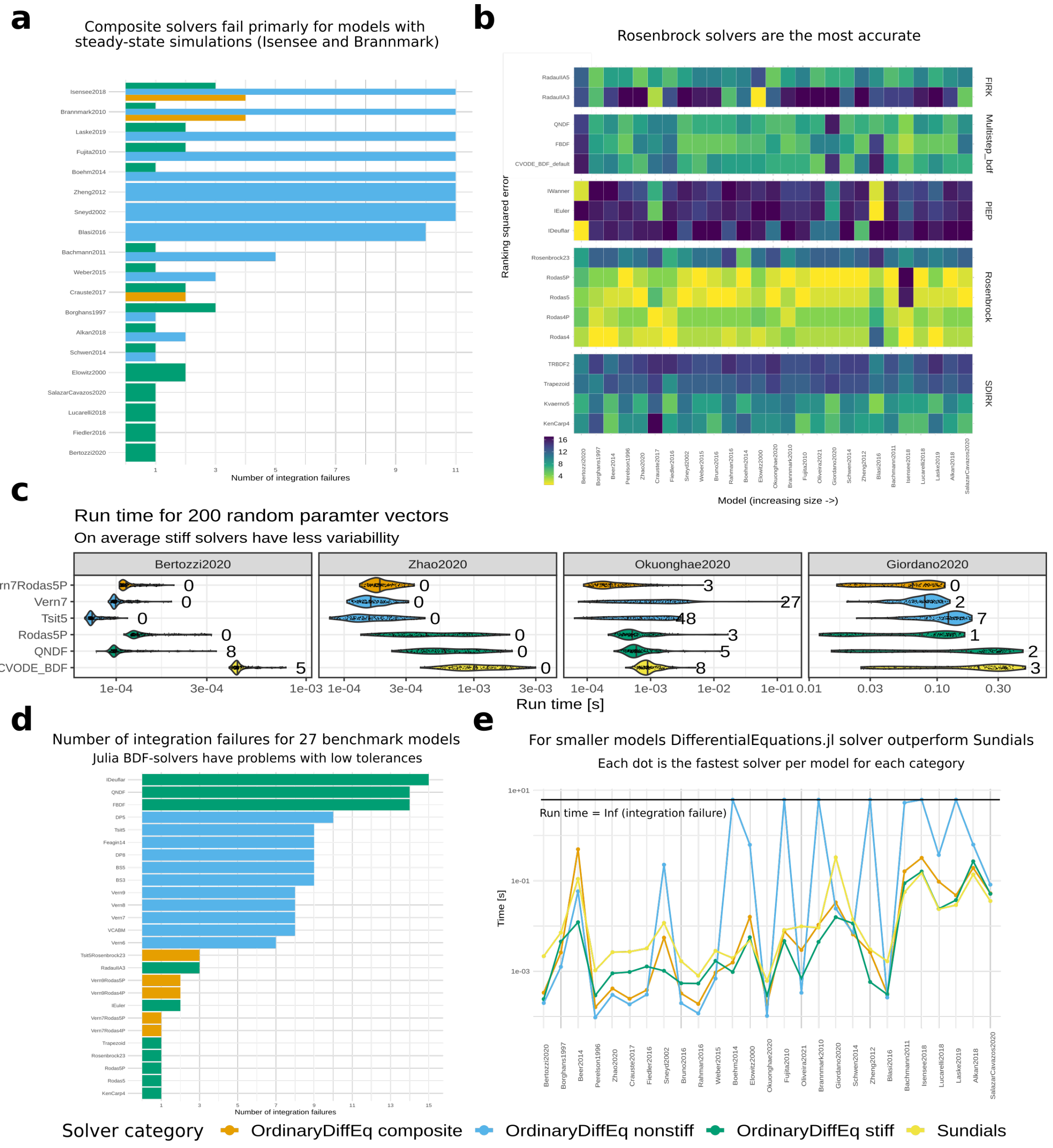

Figure 8: Benchmark of Sundial and DifferentialEquations ODE solvers for 27 models. a) Integration failures per model for a large selection of stiff solvers when using $abstol = reltol = 1 \times 10^{-8}$ **b)** Accuracy ranking per model for a large selection of stiff solvers grouped into their respective solver family when using the tolerances in a). A bright yellow color denotes a good ranking. Accuracy was computed by taking the L_2-norm for all time points with observed data for the relevant solver against a high accuracy solution computed with the Rodas5P with tolerances $abstol = reltol = 1 \times 10^{-15}$. **b)** Run-time across 100 random parameter vectors for 4 SIR models using non-stiff, stiff, and composite solvers that performed well. The centre line of each violin plot denotes the median, and the number at the end of the violin shows the number of integration failures for that solver. **d)** Total number of integration failures across all models with the tolerances when using the tolerances $abstol = 1 \times 10^{-16}$ and $reltol = 1 \times 10^{-8}$ b). **e)** Best average run-time (5 repetitions) for each solver category for all benchmark models using the tolerances in d).

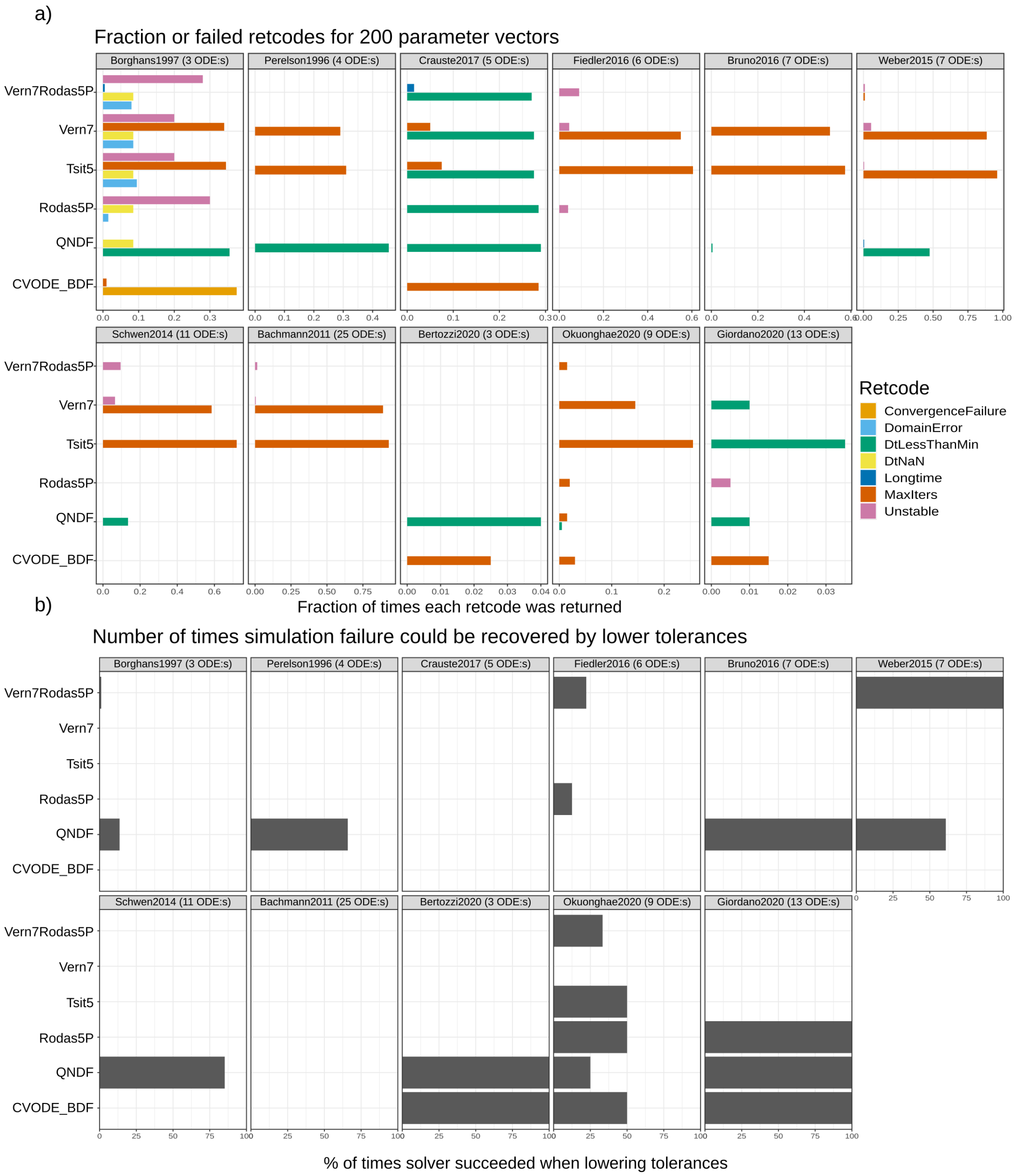

Figure 9: ODE solver error codes when testing solvers for random parameters. **a)** Fraction number of times each error appeared when simulating $n = 200$ random parameter vectors in Fig. 2 and Fig. 8. For example, for the Borghans model, CVODE_BDF failed for around $0.35 * 200 = 70$ cases with error code ConvergenceFailure. Only models where simulation failures occurred are included, so the Zhao model is excluded from the figure. **b)** Percentage of times simulation could be recovered by reducing ODE solver tolerances from $abstol = reltol = 1 \times 10^{-8}$ in steps of 10 to $abstol = reltol = 1 \times 10^{-4}$. For example, for the Bertozzi model, all simulation failures for CVODE_BDF could be resolved by increasing solver tolerances.

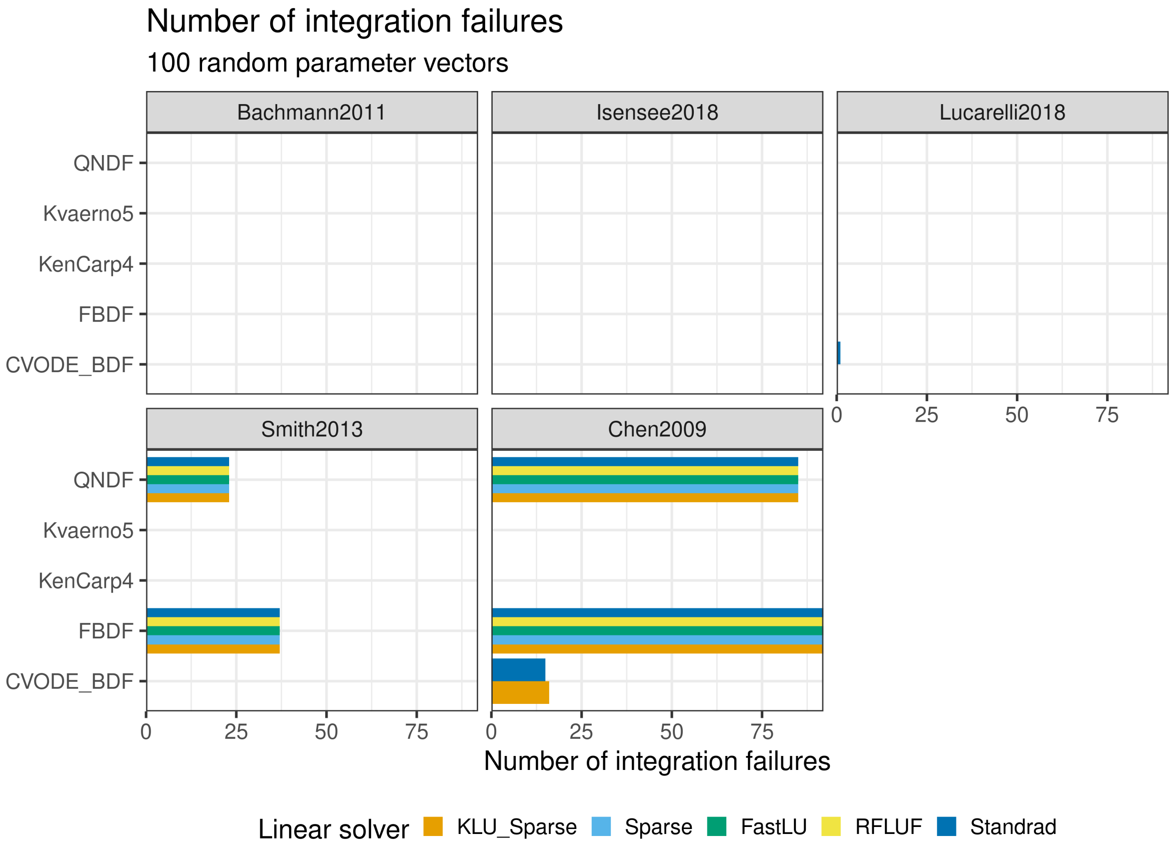

Figure 10: Integration failures when trying ODE solvers for large models. Number of integration failures for the 100 random parameter vectors in Fig. 3. Noticeably, the DifferentialEquations BDF solvers (QNDF and FBDF) frequently fail for the two bigger Smith and Chen models.

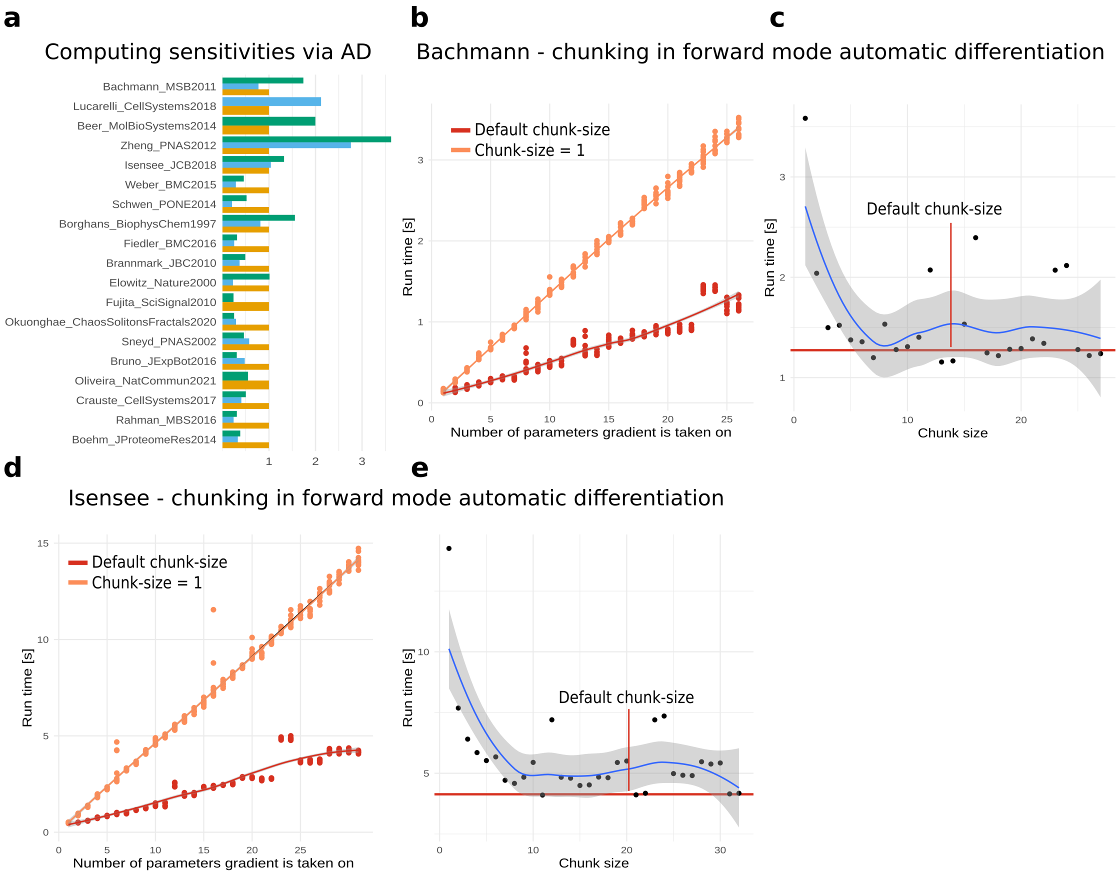

Figure 11: Gradient benchmark for smaller models. a) Run-time evaluation for the gradient at reported parameter values. AMICI computes the gradient by solving for the sensitivities via an expanded ODE system, while the Julia importer solves for the sensitives via Forward-mode AD. Run times are normalized with respect to AMICI. **b)** Gradient run-time for the Bachmann model when increasing the number of parameters using forward-mode AD without chunking and with the default number of chunks. **c)** Gradient run-time for the Bachmann model when taking the gradient on all parameters and testing all chunk sizes. **d-e** As in b-c) but for the Isensee model

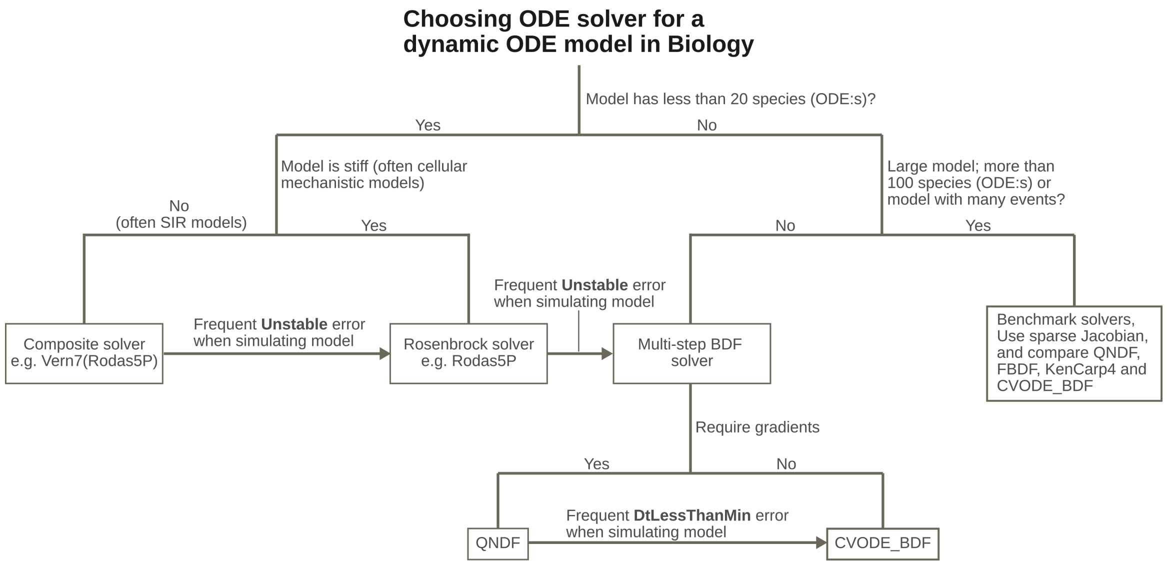

Figure 12: Flowchart for ODE solver choice for dynamic models in biology. This is a general recommendation based on the benchmarks (Fig. 2, 3, 8, 9, 10), but it should be kept in mind that every problem is unique, and even though the settings here generally work well, they might not be optimal. For larger models (right part), we recommend benchmarking different solvers, as the runtime is substantial in this regime, so getting the best performance is extra important.

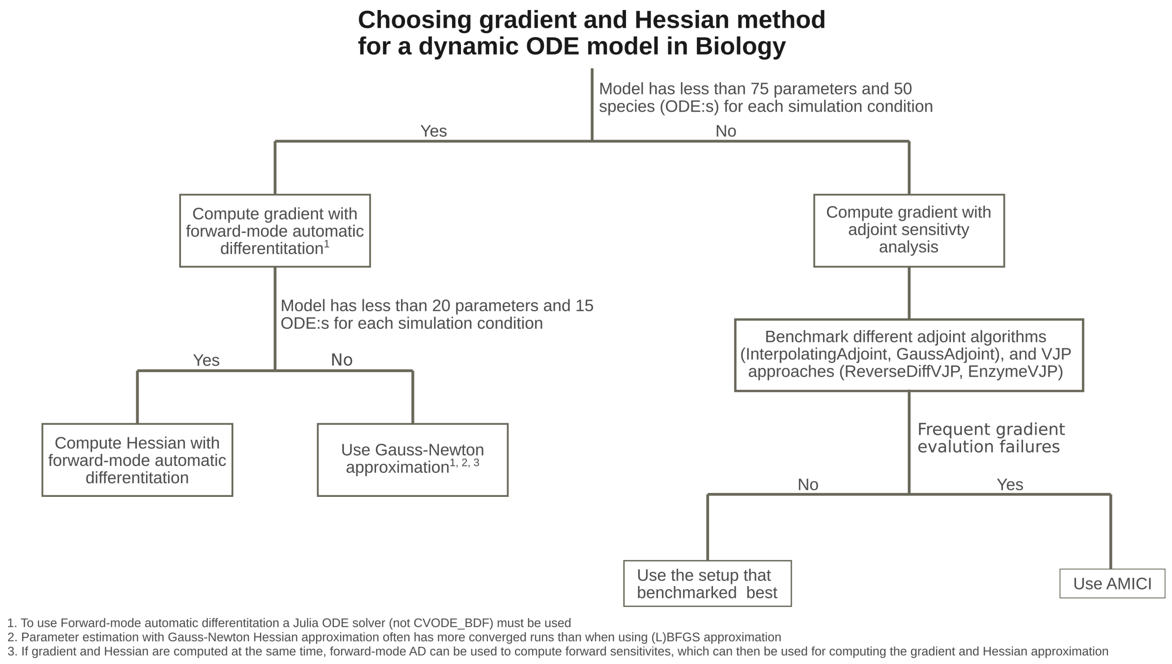

Figure 13: Flowchart for gradient and Hessian method choice for dynamic models in biology. This is a general recommendation based on the benchmarks (Fig. 4, 5, 11), but it should be kept in mind that any problem is unique, and even though the settings here generally work well, they might not be optimal and/or change in the future. For larger models (right part), we recommend benchmarking different methods, as the runtime is substantial in this regime, getting the best performance is extra important.

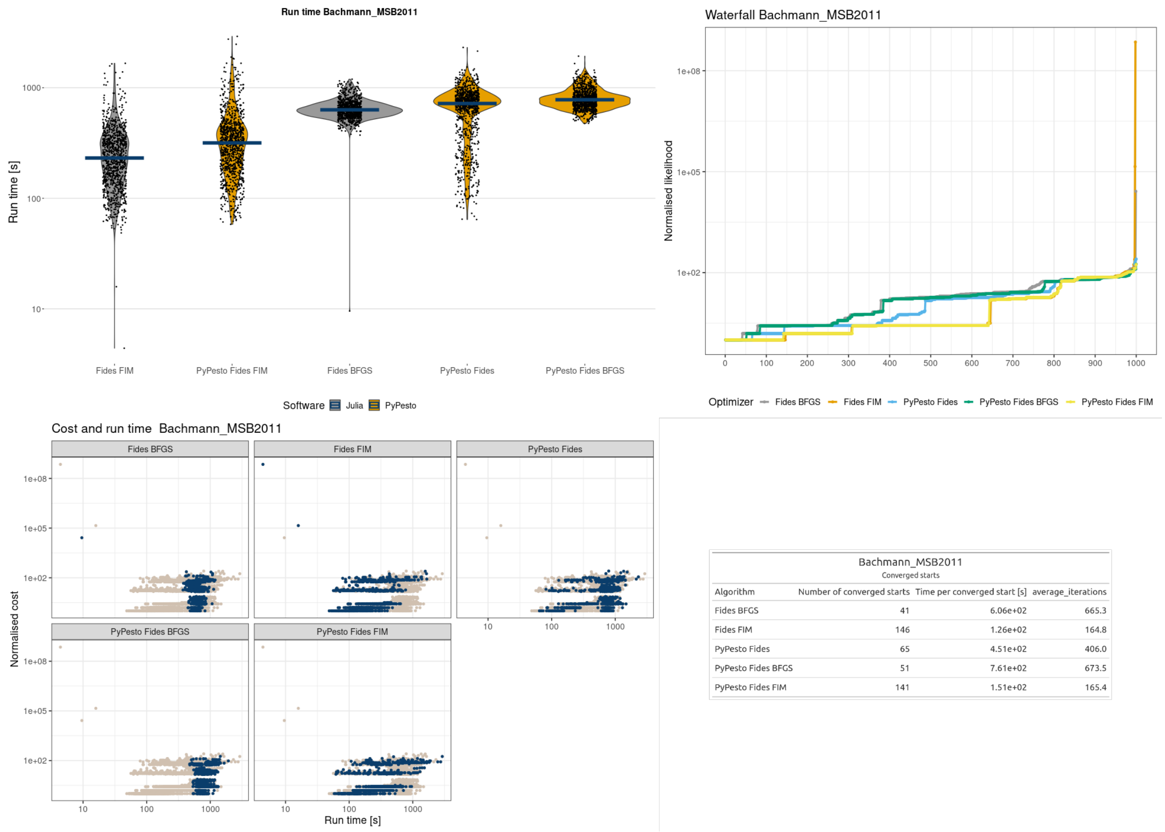

Bachmann_MSB2011
Converged starts

Algorithm	Number of converged starts	Time per converged start [s]	average_iterations
Fides BFGS	41	6.06e+02	665.3
Fides FIM	146	1.26e+02	164.8
PyPesto Fides	65	4.51e+02	406.0
PyPesto Fides BFGS	51	7.61e+02	673.5
PyPesto Fides FIM	141	1.51e+02	165.4

Figure 14: Parameter estimation results for the Bachmann model. **(a)** Run times for each optimization setting sorted based on run time. The line denotes the median. **(b)** Waterfall plot for each optimizer. The y-axis is translated such that the minimum value equals 1. **(c)** Normalized run time versus actual run time for each optimizer option. The blue dots in each panel correspond to the optimizer option in the heading. **(d)** Summary statistics for the optimizers that converged to the best-found optima.

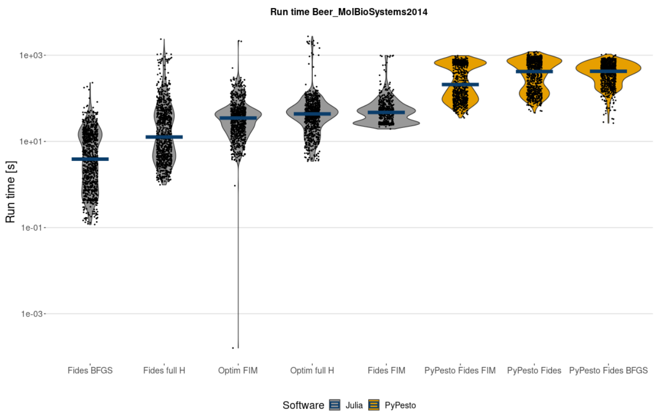

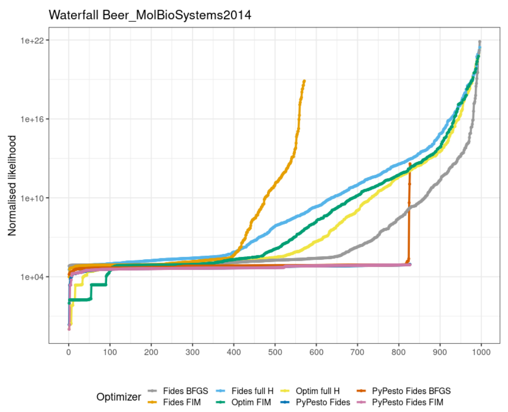

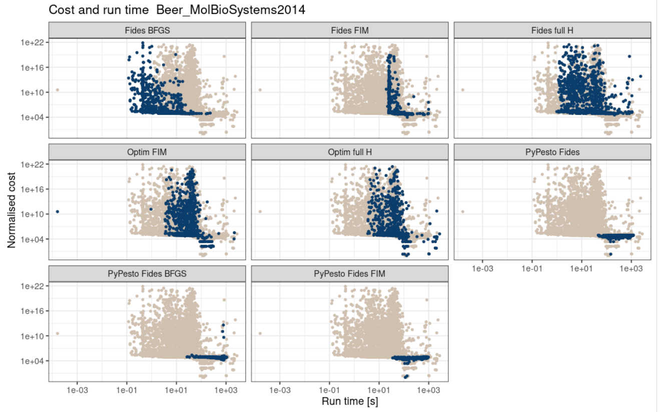

Beer_MolBioSystems2014

Converged starts

Algorithm	Number of converged starts	Time per converged start [s]	average_iterations
Optim full H	1	1.29e+02	124.0
PyPesto Fides FIM	1	1.14e+02	190.0

Figure 15: Parameter estimation results for the Beer model. **(a)**Run times for each optimization setting sorted based on run time. The line denotes the median. **(b)** Waterfall plot for each optimizer. The y-axis is translated such that the minimum value equals 1. **(c)** Normalized run time versus actual run time for each optimizer option. The blue dots in each panel correspond to the optimizer option in the heading. **(d)** Summary statistics for the optimizers that converged to the best-found optima.

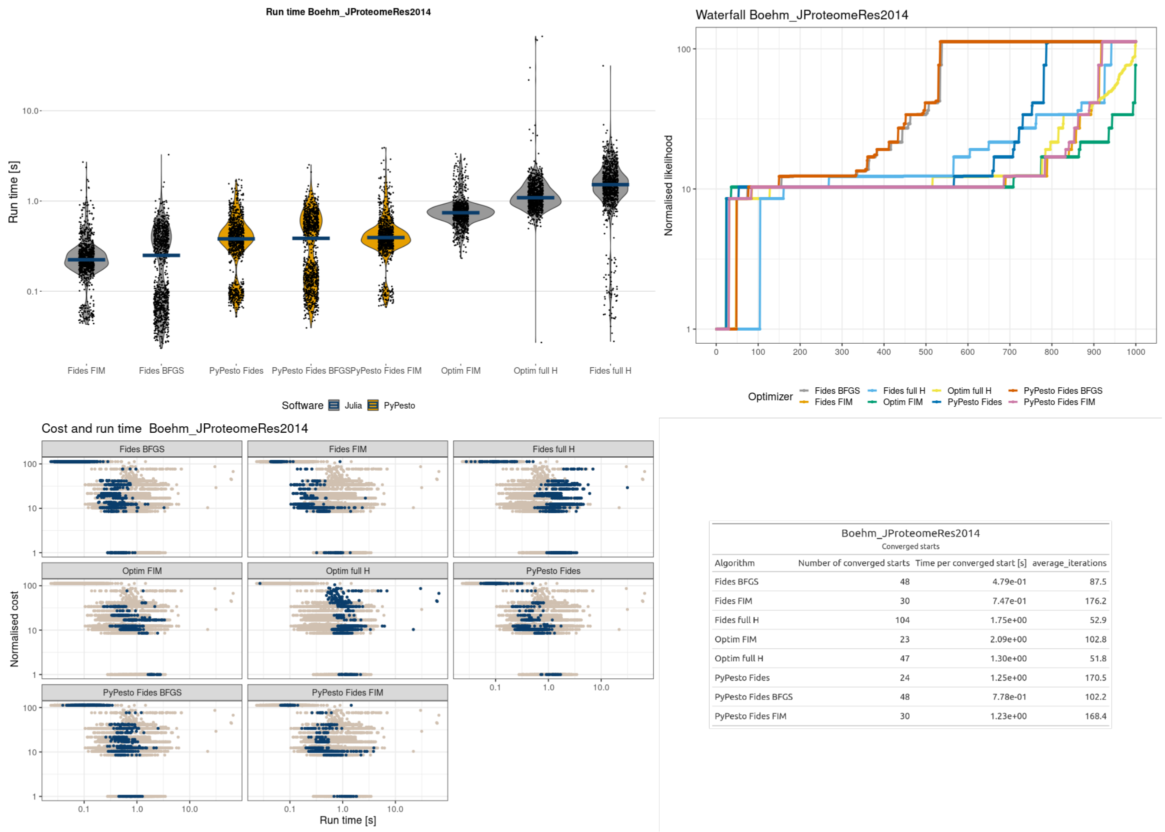

Boehm_JProteomeRes2014

Converged starts

Algorithm	Number of converged starts	Time per converged start [s]	average_iterations
Fides BFGS	48	4.79e-01	87.5
Fides FIM	30	7.47e-01	176.2
Fides full H	104	1.75e+00	52.9
Optim FIM	23	2.09e+00	102.8
Optim full H	47	1.30e+00	51.8
PyPesto Fides	24	1.25e+00	170.5
PyPesto Fides BFGS	48	7.78e-01	102.2
PyPesto Fides FIM	30	1.23e+00	168.4

Figure 16: Parameter estimation results for the Boehm model. **(a)**Run times for each optimization setting are sorted based on run time. The line denotes the median. **(b)** Waterfall plot for each optimizer. The y-axis is translated such that the minimum value equals 1. **(c)** Normalized run time versus actual run time for each optimizer option. The blue dots in each panel correspond to the optimizer option in the heading. **(d)** Summary statistics for the optimizers that converged to the best-found optima.

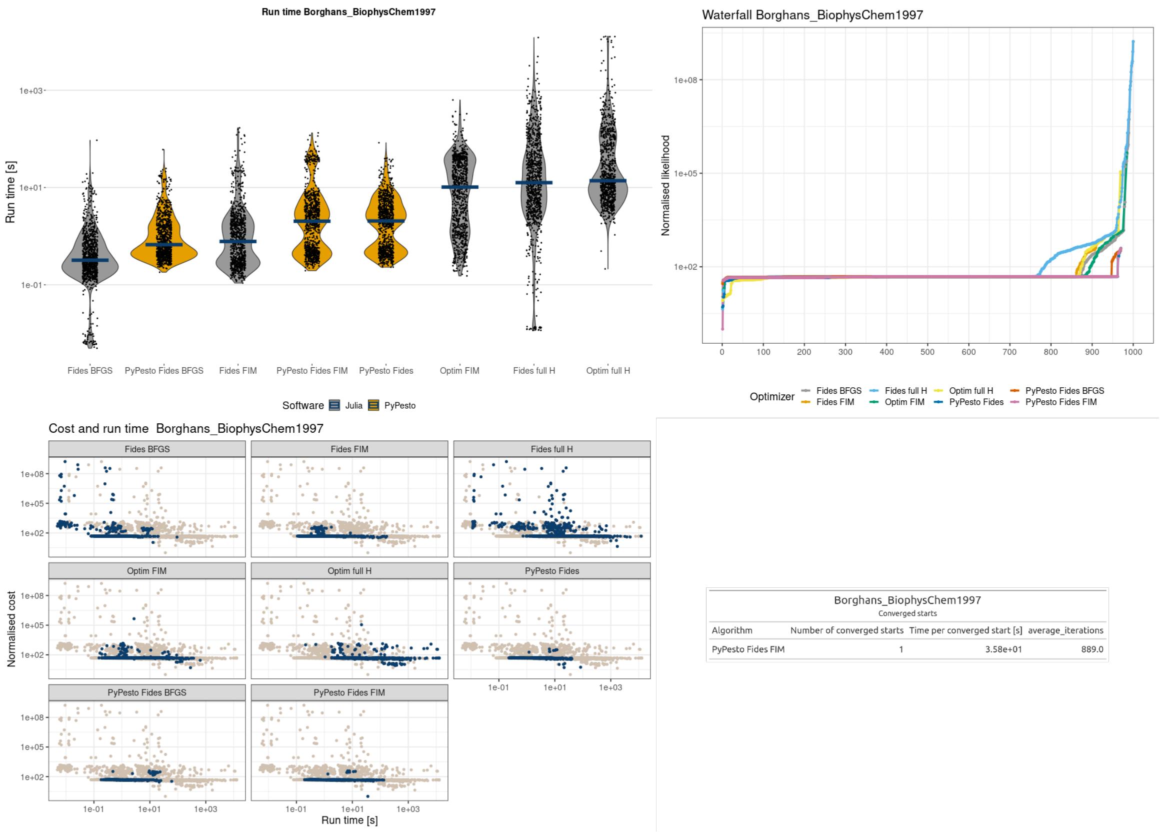

Borghans_BiophysChem1997

Converged starts

Algorithm	Number of converged starts	Time per converged start [s]	average_iterations
PyPesto Fides FIM	1	3.58e+01	889.0

Figure 17: Parameter estimation results for the Borghans model. **(a)** Run times for each optimization setting are sorted based on run time. The line denotes the median. **(b)** Waterfall plot for each optimizer. The y-axis is translated such that the minimum value equals 1. **(c)** Normalized run time versus actual run time for each optimizer option. The blue dots in each panel correspond to the optimizer option in the heading. **(d)** Summary statistics for the optimizers that converged to the best-found optima.

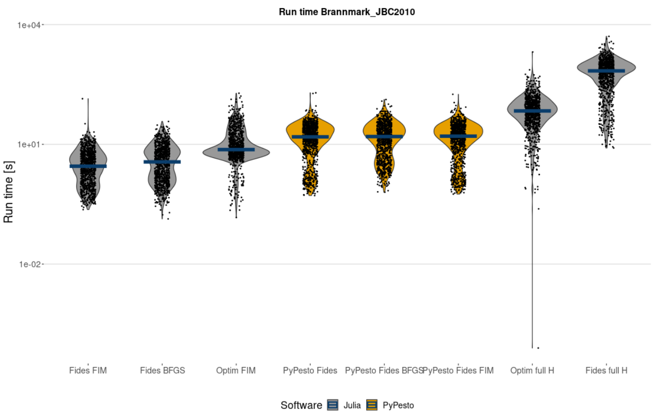

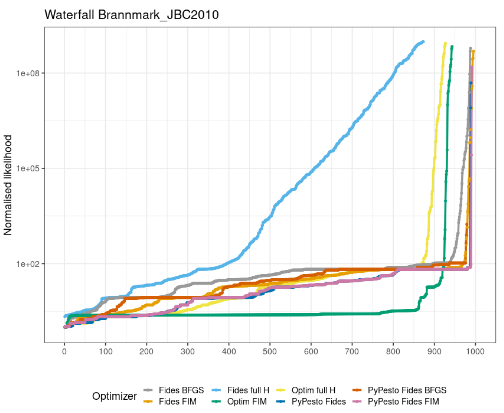

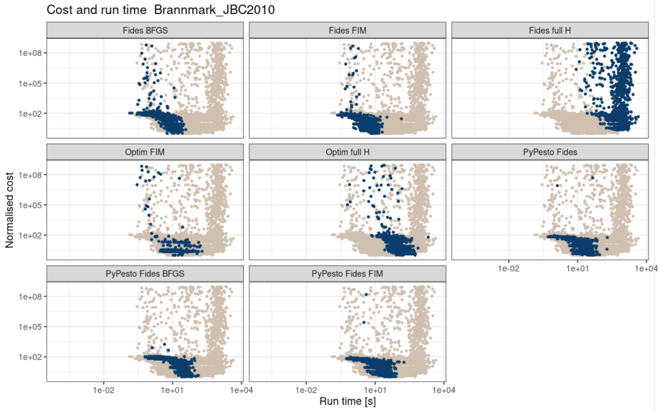

Brannmark_JBC2010

Converged starts

Algorithm	Number of converged starts	Time per converged start [s]	average_iterations
Fides BFGS	5	1.56e+01	547.8
Fides FIM	13	9.70e+00	305.8
Optim FIM	5	1.59e+01	130.8
Optim full H	5	2.11e+02	157.4
PyPesto Fides	17	3.65e+01	414.3
PyPesto Fides BFGS	10	4.80e+01	587.0
PyPesto Fides FIM	12	4.20e+01	485.2

Figure 18: Parameter estimation results for the Brannmark model. **(a)**Run times for each optimization setting are sorted based on run time. The line denotes the median. **(b)** Waterfall plot for each optimizer. The y-axis is translated such that the minimum value equals 1. **(c)** Normalized run time versus actual run time for each optimizer option. The blue dots in each panel correspond to the optimizer option in the heading. **(d)** Summary statistics for the optimizers that converged to the best-found optima.

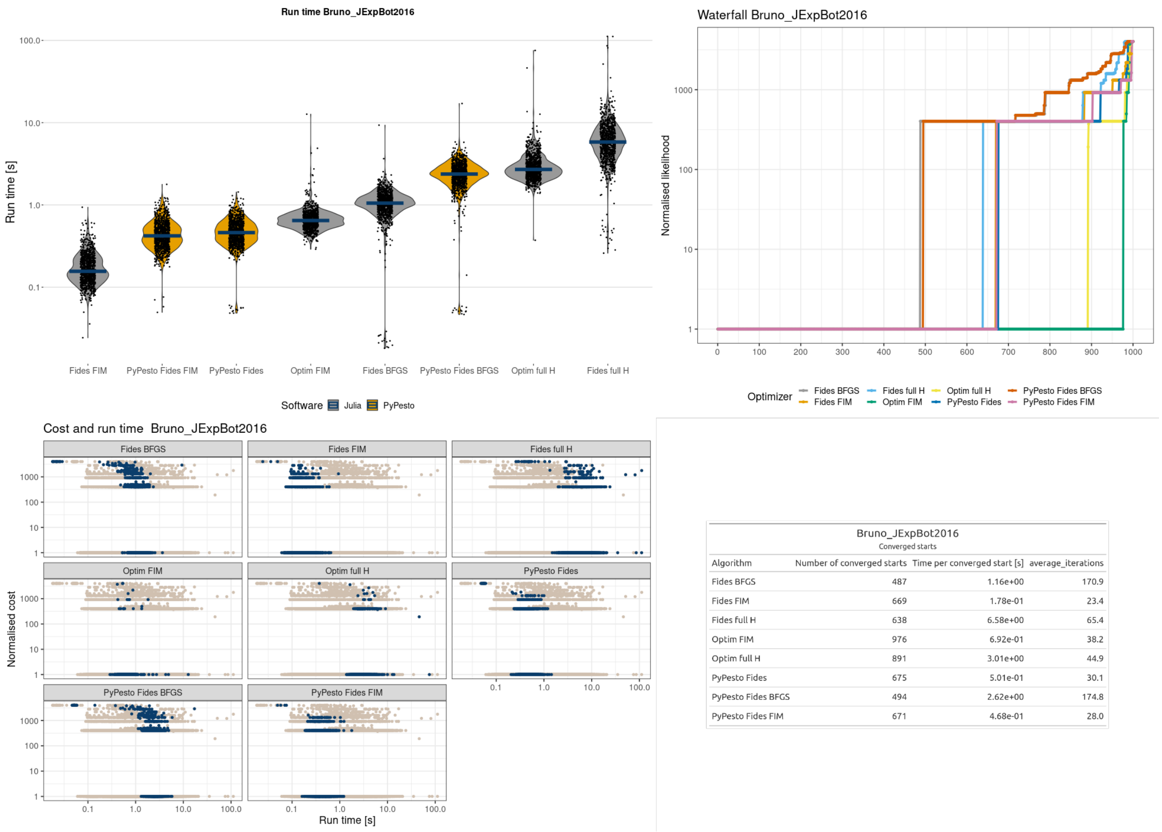

Bruno_JExpBot2016

Converged starts

Algorithm	Number of converged starts	Time per converged start [s]	average_iterations
Fides BFGS	487	1.16e+00	170.9
Fides FIM	669	1.78e-01	23.4
Fides full H	638	6.58e+00	65.4
Optim FIM	976	6.92e-01	38.2
Optim full H	891	3.01e+00	44.9
PyPesto Fides	675	5.01e-01	30.1
PyPesto Fides BFGS	494	2.62e+00	174.8
PyPesto Fides FIM	671	4.68e-01	28.0

Figure 19: Parameter estimation results for the Bruno model. **(a)**Run times for each optimization setting are sorted based on run time. The line denotes the median. **(b)** Waterfall plot for each optimizer. The y-axis is translated such that the minimum value equals 1. **(c)** Normalized run time versus actual run time for each optimizer option. The blue dots in each panel correspond to the optimizer option in the heading. **(d)** Summary statistics for the optimizers that converged to the best-found optima.

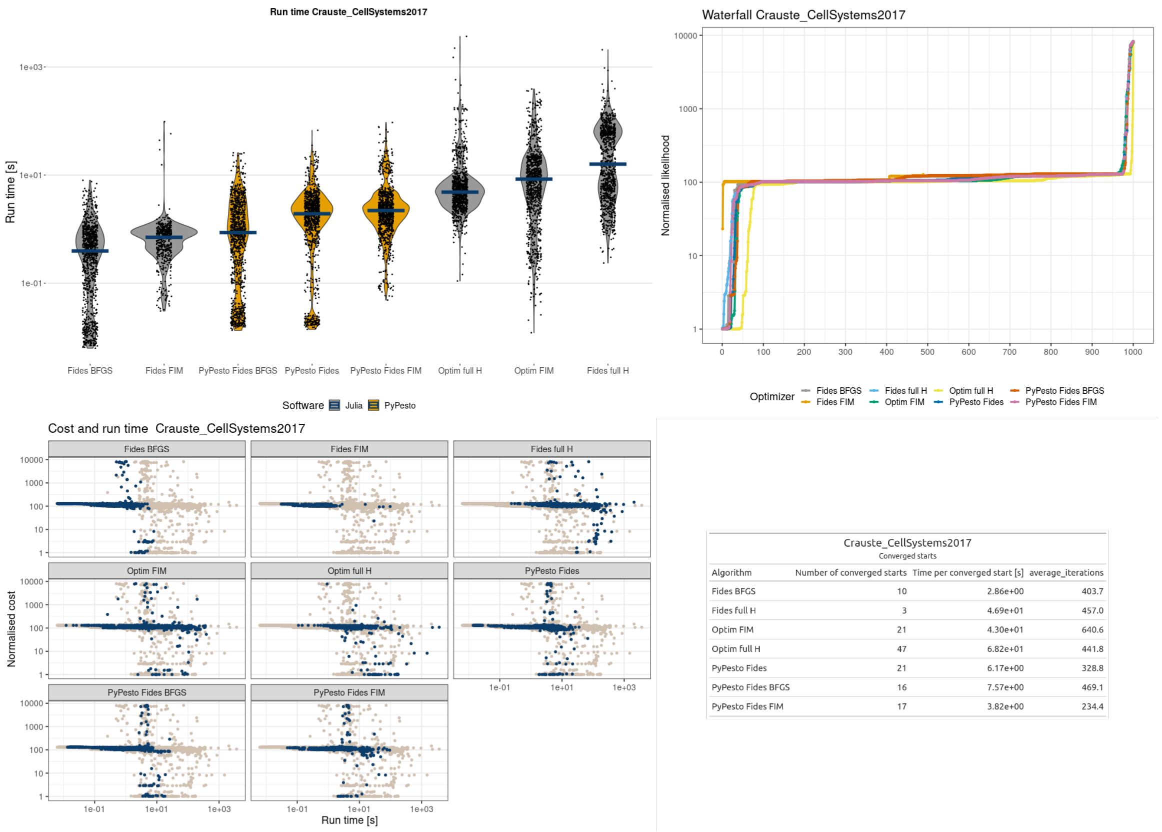

Crauste_CellSystems2017

Converged starts

Algorithm	Number of converged starts	Time per converged start [s]	average_iterations
Fides BFGS	10	2.86e+00	403.7
Fides full H	3	4.69e+01	457.0
Optim FIM	21	4.30e+01	640.6
Optim full H	47	6.82e+01	441.8
PyPesto Fides	21	6.17e+00	328.8
PyPesto Fides BFGS	16	7.57e+00	469.1
PyPesto Fides FIM	17	3.82e+00	234.4

Figure 20: Parameter estimation results for the Crauste model. **(a)**Run times for each optimization setting are sorted based on run time. The line denotes the median. **(b)** Waterfall plot for each optimizer. The y-axis is translated such that the minimum value equals 1. **(c)** Normalized run time versus actual run time for each optimizer option. The blue dots in each panel correspond to the optimizer option in the heading. **(d)** Summary statistics for the optimizers that converged to the best-found optima.

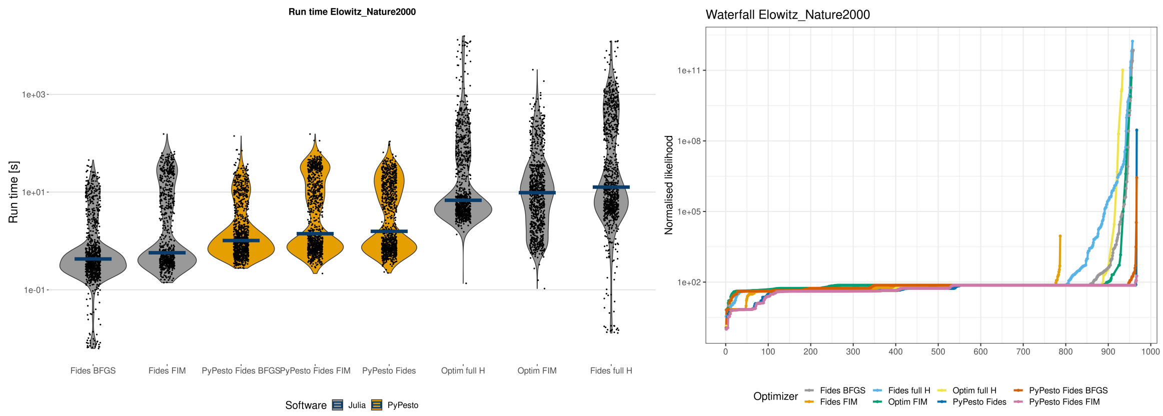

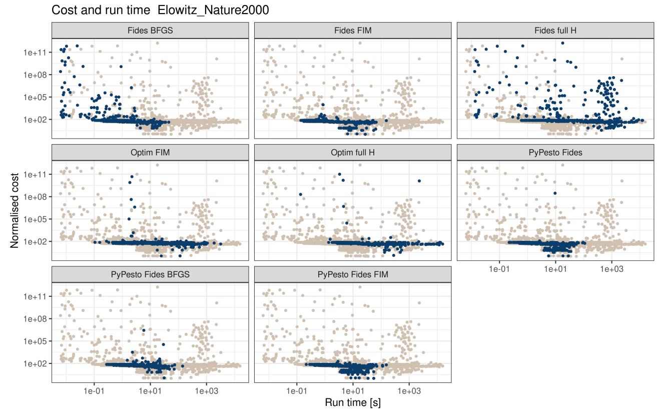

Elowitz_Nature2000

Converged starts

Algorithm	Number of converged starts	Time per converged start [s]	average_iterations
Fides FIM	1	2.74e+01	482.0
PyPesto Fides FIM	2	4.46e+01	477.5

Figure 21: Parameter estimation results for the Elowitz model. **(a)**Run times for each optimization setting are sorted based on run time. The line denotes the median. **(b)** Waterfall plot for each optimizer. The y-axis is translated such that the minimum value equals 1. **(c)** Normalized run time versus actual run time for each optimizer option. The blue dots in each panel correspond to the optimizer option in the heading. **(d)** Summary statistics for the optimizers that converged to the best-found optima.

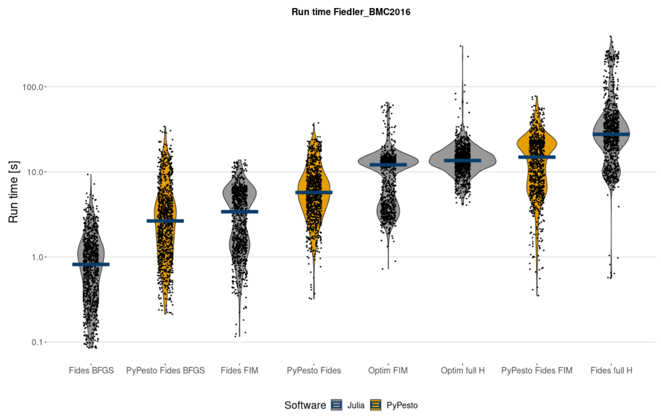

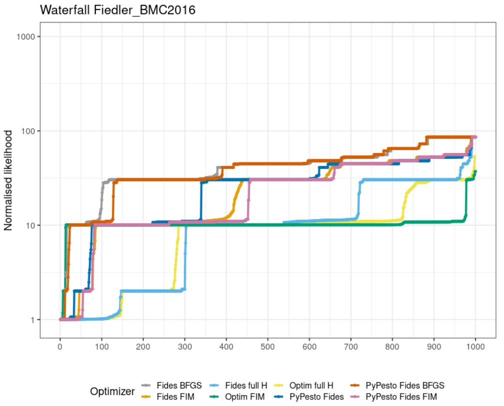

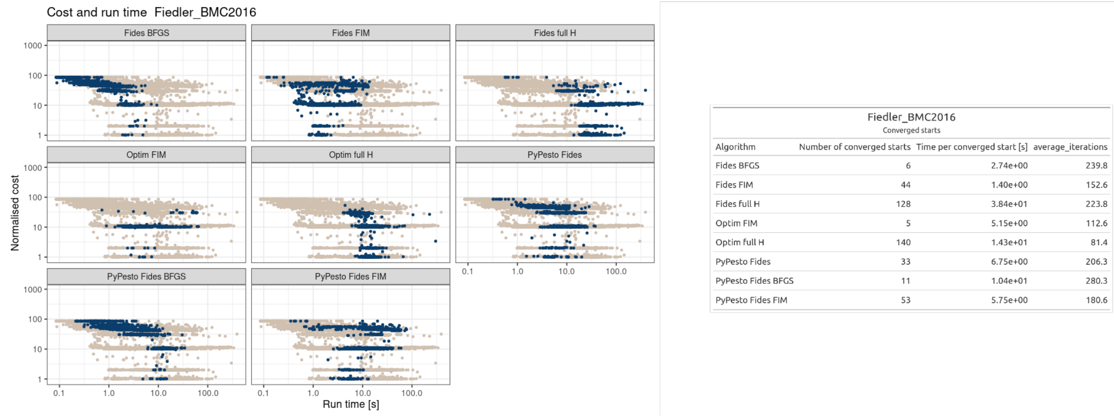

Fiedler_BMC2016

Converged starts

Algorithm	Number of converged starts	Time per converged start [s]	average_iterations
Fides BFGS	6	2.74e+00	239.8
Fides FIM	44	1.40e+00	152.6
Fides full H	128	3.84e+01	223.8
Optim FIM	5	5.15e+00	112.6
Optim full H	140	1.43e+01	81.4
PyPesto Fides	33	6.75e+00	206.3
PyPesto Fides BFGS	11	1.04e+01	280.3
PyPesto Fides FIM	53	5.75e+00	180.6

Figure 22: Parameter estimation results for the Fiedler model. **(a)**Run times for each optimization setting are sorted based on run time. The line denotes the median. **(b)** Waterfall plot for each optimizer. The y-axis is translated such that the minimum value equals 1. **(c)** Normalized run time versus actual run time for each optimizer option. The blue dots in each panel correspond to the optimizer option in the heading. **(d)** Summary statistics for the optimizers that converged to the best-found optima.

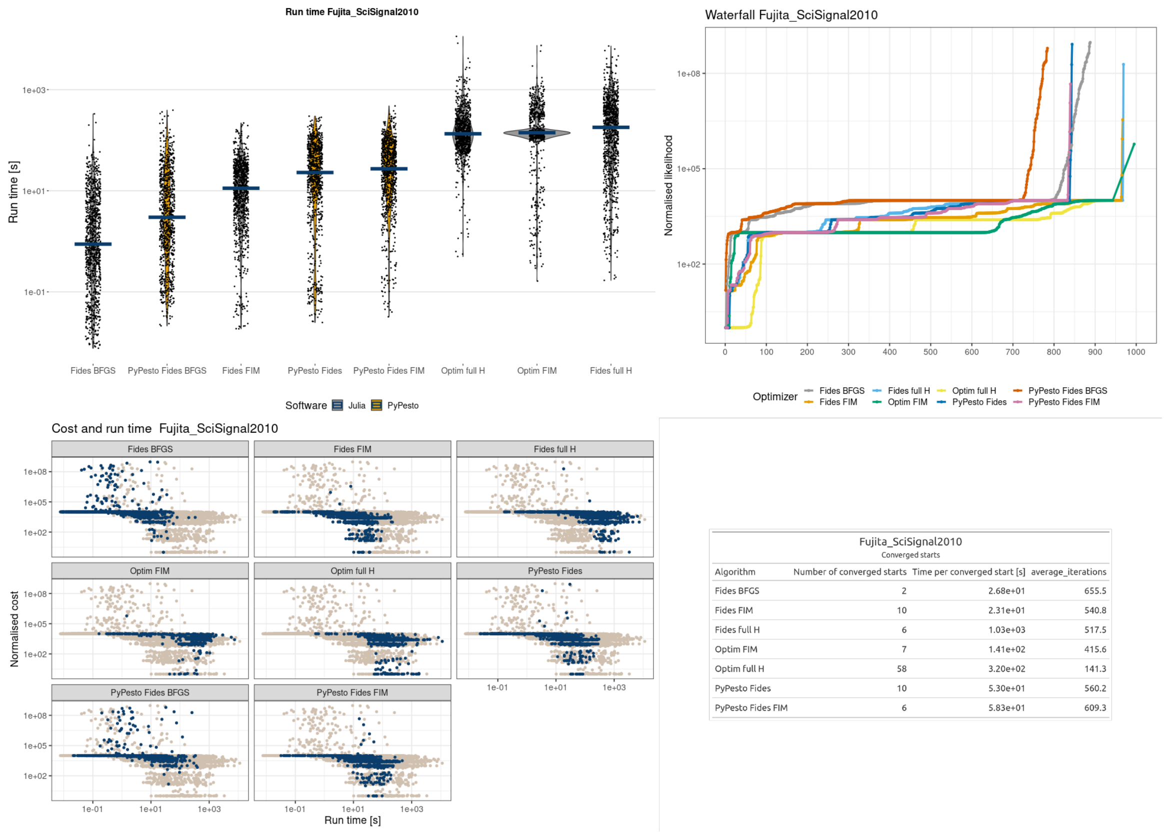

Fujita_SciSignal2010

Converged starts

Algorithm	Number of converged starts	Time per converged start [s]	average_iterations
Fides BFGS	2	2.68e+01	655.5
Fides FIM	10	2.31e+01	540.8
Fides full H	6	1.03e+03	517.5
Optim FIM	7	1.41e+02	415.6
Optim full H	58	3.20e+02	141.3
PyPesto Fides	10	5.30e+01	560.2
PyPesto Fides FIM	6	5.83e+01	609.3

Figure 23: Parameter estimation results for the Fujita model. (a) Run times for each optimization setting sorted based on run time. The line denotes the median. **(b)** Waterfall plot for each optimizer. The y-axis is translated such that the minimum value equals 1. **(c)** Normalized run time versus actual run time for each optimizer option. The blue dots in each panel correspond to the optimizer option in the heading. **(d)** Summary statistics for the optimizers that converged to the best-found optima.

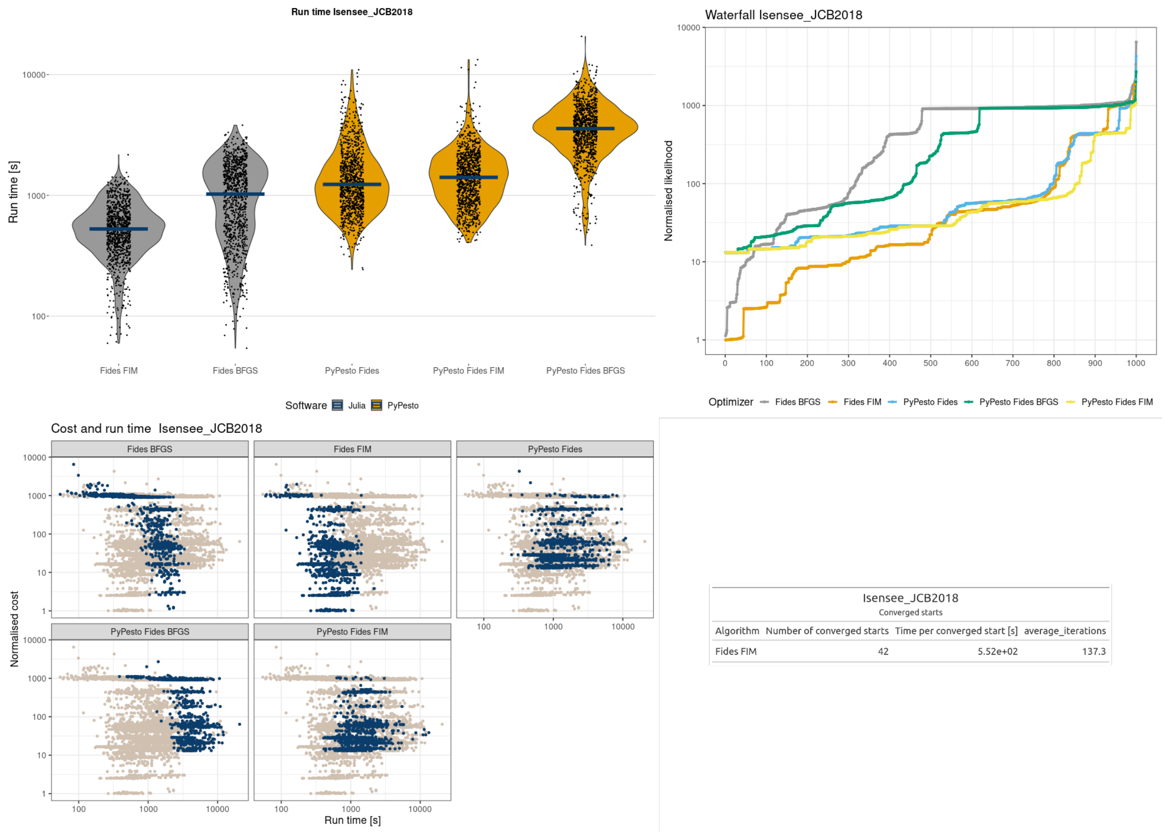

Algorithm	Number of converged starts	Time per converged start [s]	average_iterations
Fides FIM	42	5.52e+02	137.3

Figure 24: Parameter estimation results for the Isensee model. **(a)**Run times for each optimization setting are sorted based on run time. The line denotes the median. **(b)** Waterfall plot for each optimizer. The y-axis is translated such that the minimum value equals 1. **(c)** Normalized run time versus actual run time for each optimizer option. The blue dots in each panel correspond to the optimizer option in the heading. **(d)** Summary statistics for the optimizers that converged to the best-found optima.

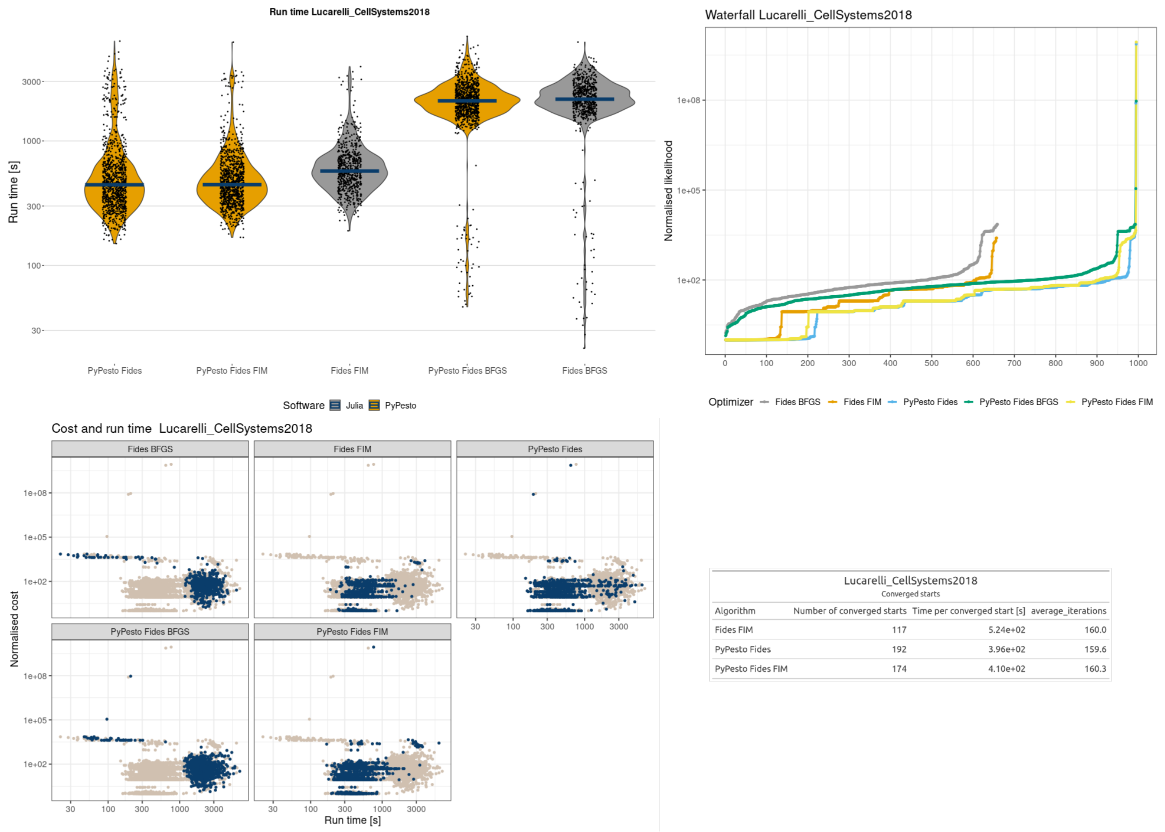

Lucarelli_CellSystems2018
Converged starts

Algorithm	Number of converged starts	Time per converged start [s]	average_iterations
Fides FIM	117	5.24e+02	160.0
PyPesto Fides	192	3.96e+02	159.6
PyPesto Fides FIM	174	4.10e+02	160.3

Figure 25: Parameter estimation results for the Lucarelli model. **(a)**Run times for each optimization setting are sorted based on run time. The line denotes the median. **(b)** Waterfall plot for each optimizer. The y-axis is translated such that the minimum value equals 1. **(c)** Normalized run time versus actual run time for each optimizer option. The blue dots in each panel correspond to the optimizer option in the heading. **(d)** Summary statistics for the optimizers that converged to the best-found optima.

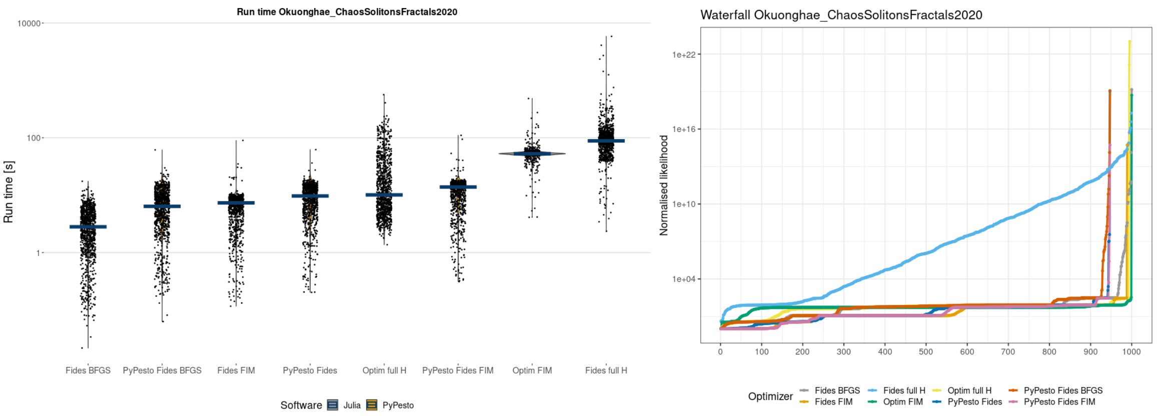

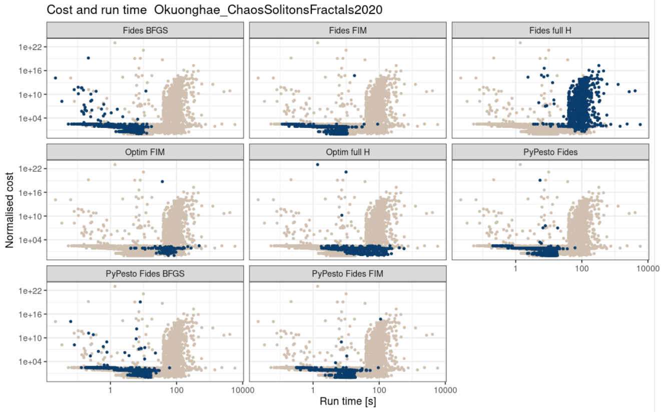

Okuonghae_ChaosSolitonsFractals2020

Converged starts

Algorithm	Number of converged starts	Time per converged start [s]	average_iterations
Fides BFGS	1	7.03e+00	841.0
Fides FIM	109	6.92e+00	742.1
Optim FIM	2	7.04e+01	807.5
Optim full H	1	4.44e+01	189.0
PyPesto Fides	40	1.26e+01	741.5
PyPesto Fides BFGS	3	1.49e+01	890.7
PyPesto Fides FIM	118	1.24e+01	745.8

Figure 26: Parameter estimation results for the Okuonghae model. (a) Run times for each optimization setting are sorted based on run time. The line denotes the median. **(b)** Waterfall plot for each optimizer. The y-axis is translated such that the minimum value equals 1. **(c)** Normalized run time versus actual run time for each optimizer option. The blue dots in each panel correspond to the optimizer option in the heading. **(d)** Summary statistics for the optimizers that converged to the best-found optima.

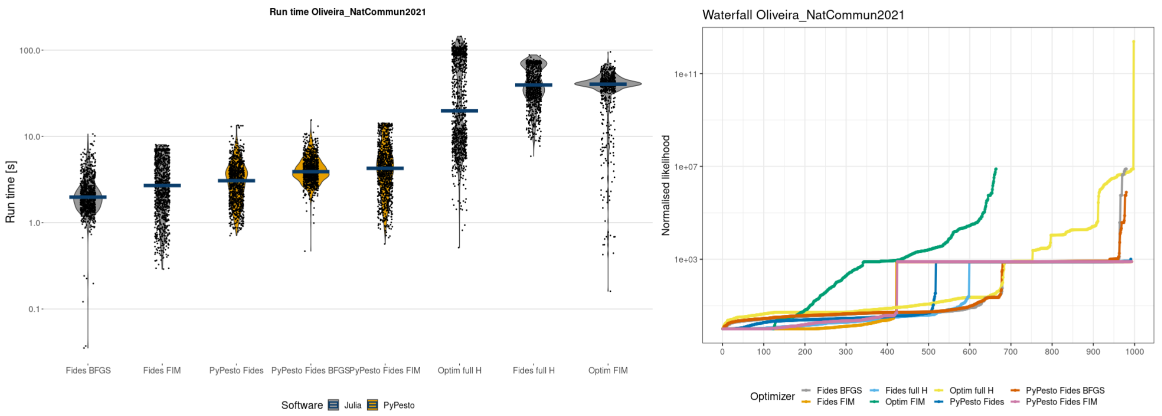

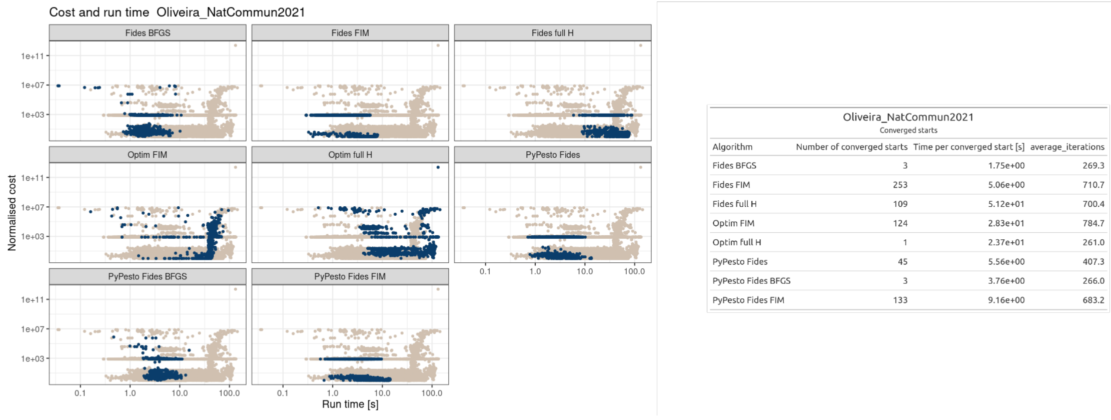

Algorithm	Number of converged starts	Time per converged start [s]	average_iterations
Fides BFGS	3	1.75e+00	269.3
Fides FIM	253	5.06e+00	710.7
Fides full H	109	5.12e+01	700.4
Optim FIM	124	2.83e+01	784.7
Optim full H	1	2.37e+01	261.0
PyPesto Fides	45	5.56e+00	407.3
PyPesto Fides BFGS	3	3.76e+00	266.0
PyPesto Fides FIM	133	9.16e+00	683.2

Oliveira_NatCommun2021 — Converged starts

Figure 27: Parameter estimation results for the Oliveira model. **(a)**Run times for each optimization setting are sorted based on run time. The line denotes the median. **(b)** Waterfall plot for each optimizer. The y-axis is translated such that the minimum value equals 1. **(c)** Normalized run time versus actual run time for each optimizer option. The blue dots in each panel correspond to the optimizer option in the heading. **(d)** Summary statistics for the optimizers that converged to the best-found optima.

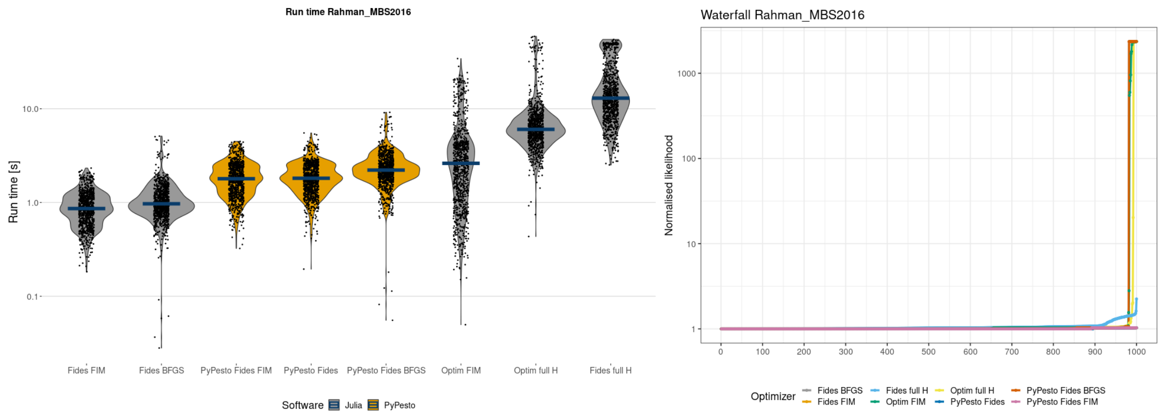

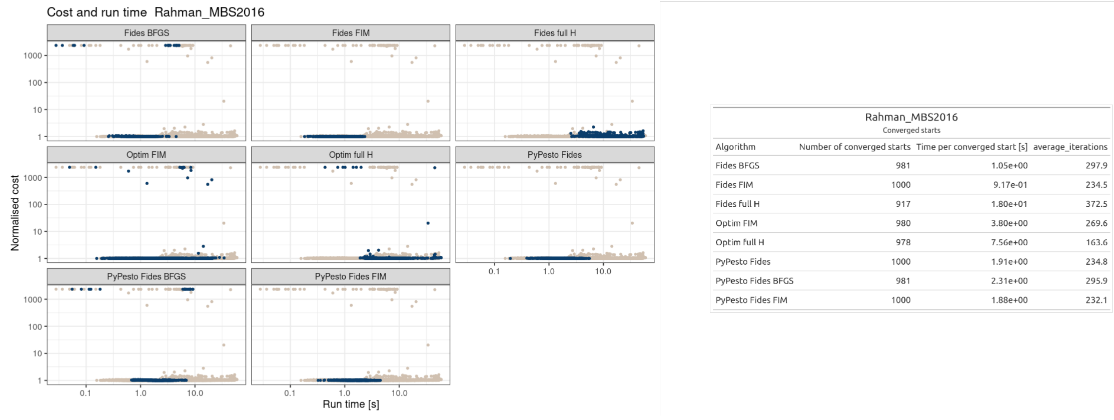

Rahman_MBS2016

Converged starts

Algorithm	Number of converged starts	Time per converged start [s]	average_iterations
Fides BFGS	981	1.05e+00	297.9
Fides FIM	1000	9.17e-01	234.5
Fides full H	917	1.80e+01	372.5
Optim FIM	980	3.80e+00	269.6
Optim full H	978	7.56e+00	163.6
PyPesto Fides	1000	1.91e+00	234.8
PyPesto Fides BFGS	981	2.31e+00	295.9
PyPesto Fides FIM	1000	1.88e+00	232.1

Figure 28: Parameter estimation results for the Rahman model. **(a)**Run times for each optimization setting are sorted based on run time. The line denotes the median. **(b)** Waterfall plot for each optimizer. The y-axis is translated such that the minimum value equals 1. **(c)** Normalized run time versus actual run time for each optimizer option. The blue dots in each panel correspond to the optimizer option in the heading. **(d)** Summary statistics for the optimizers that converged to the best-found optima.

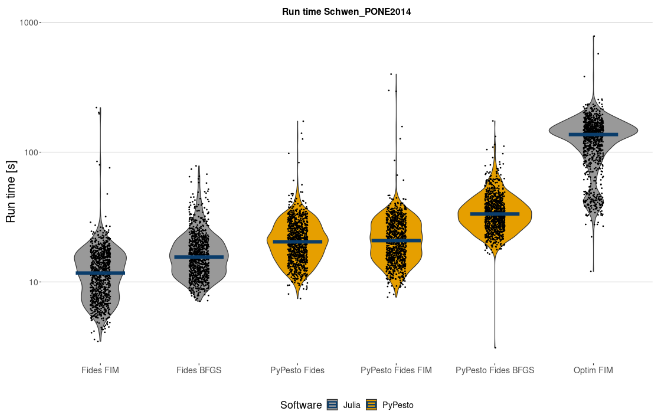

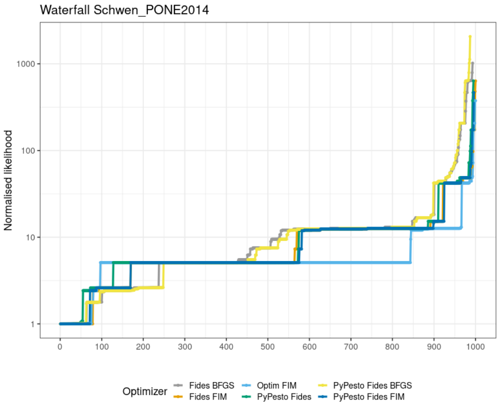

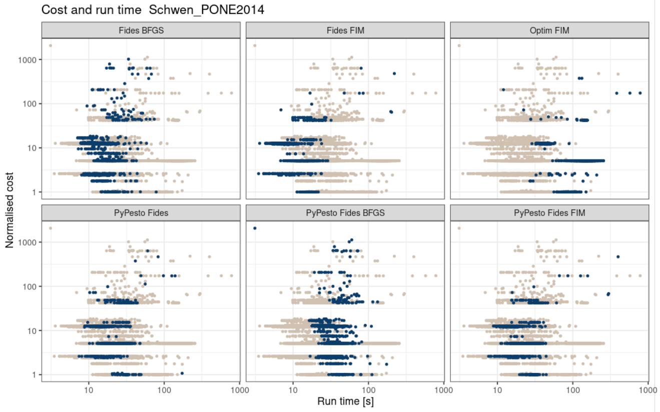

Schwen_PONE2014<br>Converged starts			
Algorithm	Number of converged starts	Time per converged start [s]	average_iterations
Fides BFGS	63	2.30e+01	275.2
Fides FIM	78	1.65e+01	165.4
Optim FIM	75	8.94e+01	179.7
PyPesto Fides	54	3.38e+01	187.1
PyPesto Fides BFGS	62	4.72e+01	278.4
PyPesto Fides FIM	71	2.94e+01	163.5

Figure 29: Parameter estimation results for the Sneyd model. **(a)**Run times for each optimization setting are sorted based on run time. The line denotes the median. **(b)** Waterfall plot for each optimizer. The y-axis is translated such that the minimum value equals 1. **(c)** Normalized run time versus actual run time for each optimizer option. The blue dots in each panel correspond to the optimizer option in the heading. **(d)** Summary statistics for the optimizers that converged to the best-found optima.

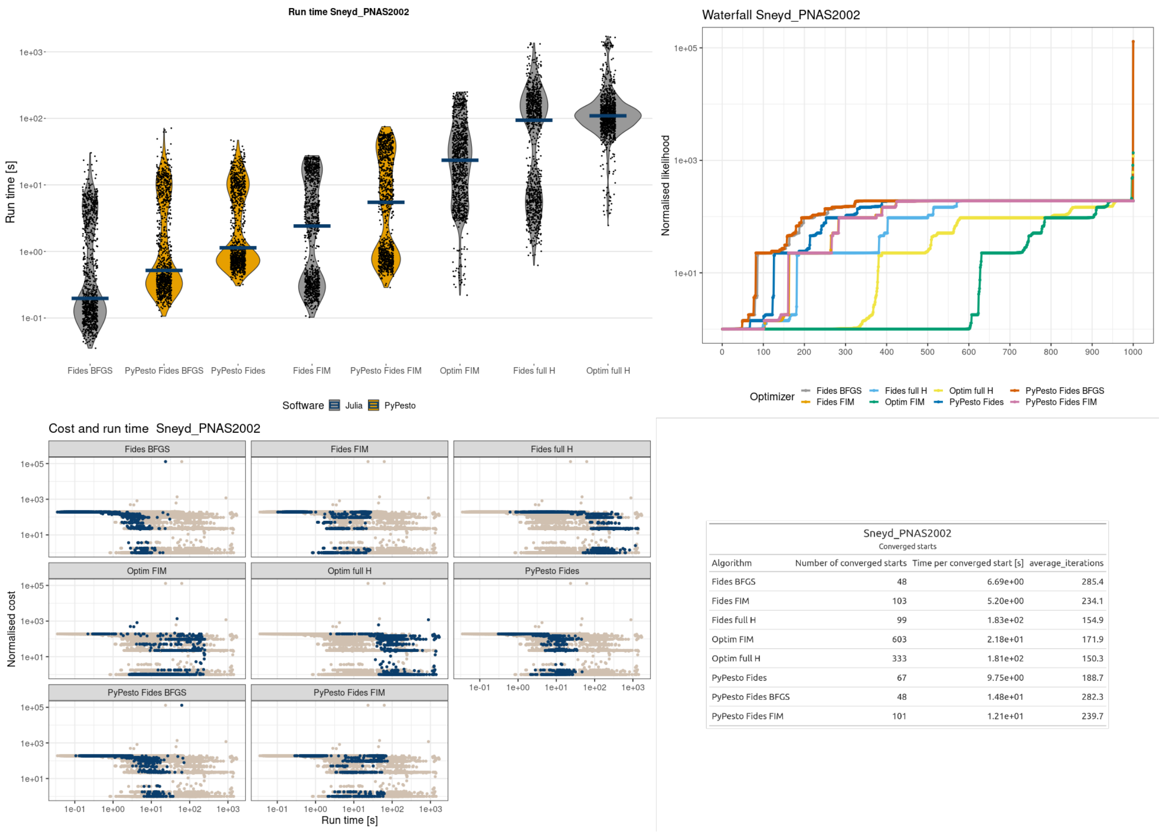

Sneyd_PNAS2002<br>Converged starts			
Algorithm	Number of converged starts	Time per converged start [s]	average_iterations
Fides BFGS	48	6.69e+00	285.4
Fides FIM	103	5.20e+00	234.1
Fides full H	99	1.83e+02	154.9
Optim FIM	603	2.18e+01	171.9
Optim full H	333	1.81e+02	150.3
PyPesto Fides	67	9.75e+00	188.7
PyPesto Fides BFGS	48	1.48e+01	282.3
PyPesto Fides FIM	101	1.21e+01	239.7

Figure 30: Parameter estimation results for the Sneyd model. **(a)**Run times for each optimization setting are sorted based on run time. The line denotes the median. **(b)** Waterfall plot for each optimizer. The y-axis is translated such that the minimum value equals 1. **(c)** Normalized run time versus actual run time for each optimizer option. The blue dots in each panel correspond to the optimizer option in the heading. **(d)** Summary statistics for the optimizers that converged to the best-found optima.

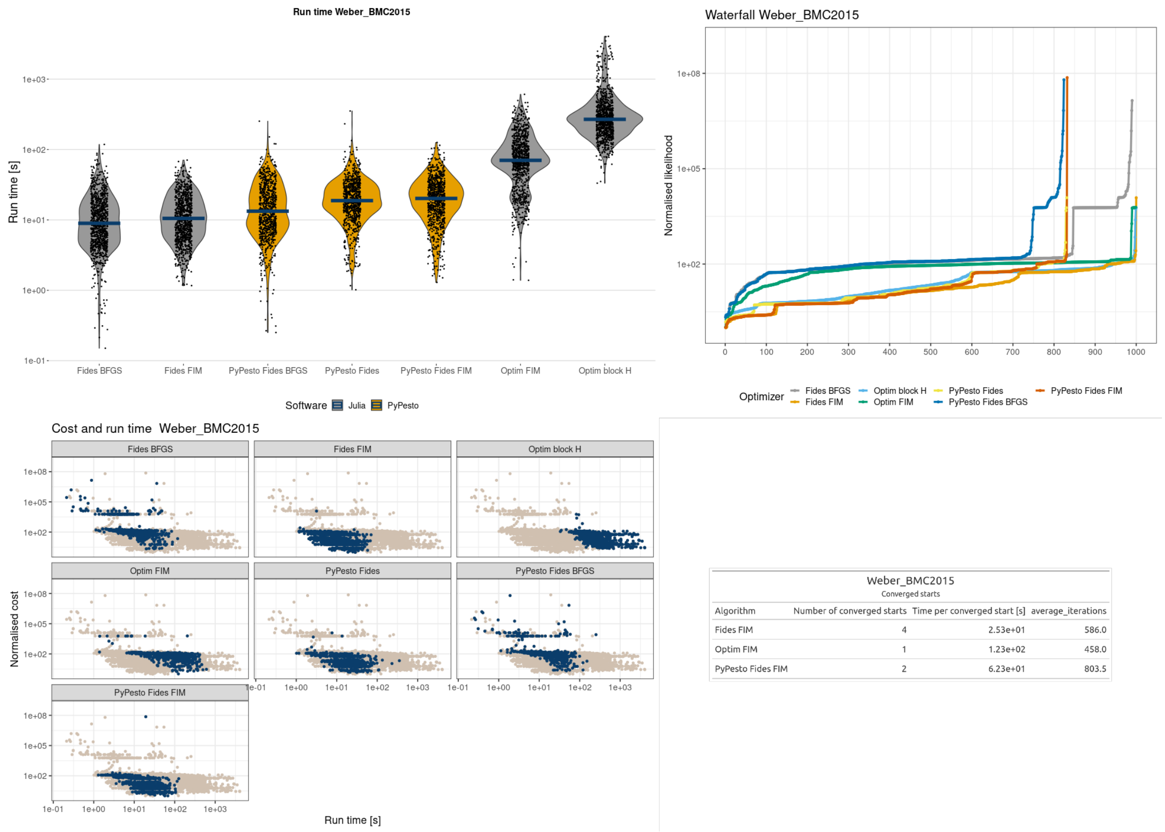

Weber_BMC2015
Converged starts

Algorithm	Number of converged starts	Time per converged start [s]	average_iterations
Fides FIM	4	2.53e+01	586.0
Optim FIM	1	1.23e+02	458.0
PyPesto Fides FIM	2	6.23e+01	803.5

Figure 31: Parameter estimation results for the Weber model. (a) Run times for each optimization setting are sorted based on run time. The line denotes the median. **(b)** Waterfall plot for each optimizer. The y-axis is translated such that the minimum value equals 1. **(c)** Normalized run time versus actual run time for each optimizer option. The blue dots in each panel correspond to the optimizer option in the heading. **(d)** Summary statistics for the optimizers that converged to the best-found optima.

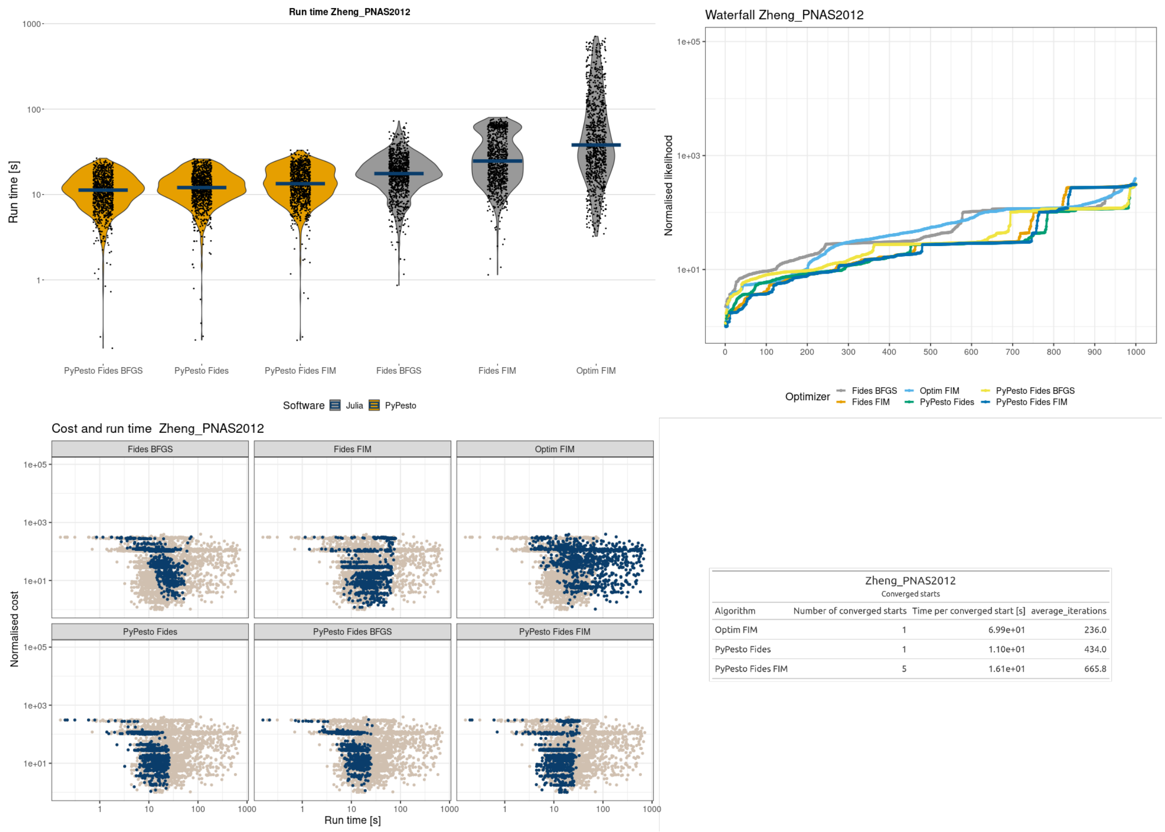

Algorithm	Number of converged starts	Time per converged start [s]	average_iterations
Optim FIM	1	6.99e+01	236.0
PyPesto Fides	1	1.10e+01	434.0
PyPesto Fides FIM	5	1.61e+01	665.8

(Table title: Zheng_PNAS2012 — Converged starts)

Figure 32: Parameter estimation results for the Zheng model. **(a)**Run times for each optimization setting are sorted based on run time. The line denotes the median. **(b)** Waterfall plot for each optimizer. The y-axis is translated such that the minimum value equals 1. **(c)** Normalized run time versus actual run time for each optimizer option. The blue dots in each panel correspond to the optimizer option in the heading. **(d)** Summary statistics for the optimizers that converged to the best-found optima.

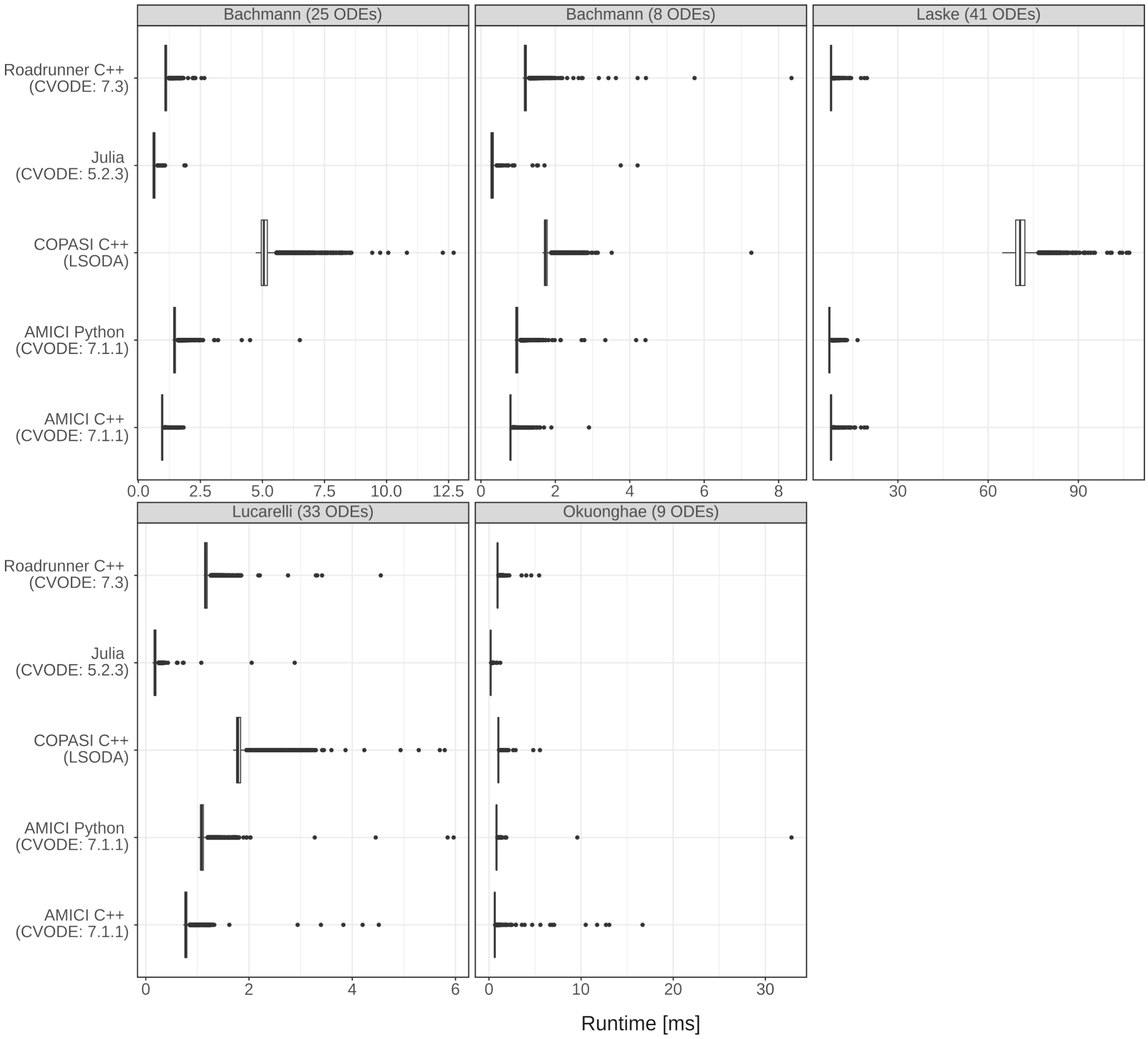

Figure 33: ODE solver time for 5 benchmark ODE models of varying size. Each model was simulated 10^5 times using the parameter values and initial conditions specified in the corresponding SBML files from the PEtab benchmark collection repository [35]. Differences in CVODE version arise from the packages wrapping different versions of the Sundials library.
